# Altered mGluR5-Homer scaffolds and corticostriatal connectivity in a *Shank3* complete knockout model of autism

**DOI:** 10.1038/ncomms11459

**Published:** 2016-05-10

**Authors:** Xiaoming Wang, Alexandra L. Bey, Brittany M. Katz, Alexandra Badea, Namsoo Kim, Lisa K. David, Lara J. Duffney, Sunil Kumar, Stephen D. Mague, Samuel W. Hulbert, Nisha Dutta, Volodya Hayrapetyan, Chunxiu Yu, Erin Gaidis, Shengli Zhao, Jin-Dong Ding, Qiong Xu, Leeyup Chung, Ramona M. Rodriguiz, Fan Wang, Richard J. Weinberg, William C. Wetsel, Kafui Dzirasa, Henry Yin, Yong-hui Jiang

**Affiliations:** 1 Department of Pediatrics, Duke University, Durham, North Carolina 27710, USA; 2 Department of Neurobiology, Duke University, Durham, North Carolina 27710, USA; 3 Department of Psychiatry and Behavioral Sciences, Duke University, Durham, North Carolina 27710, USA; 4 Department of Radiology, Duke University, Durham, North Carolina 27710, USA; 5 Department of Psychology and Neuroscience, Duke University, Durham, North Carolina 27710, USA; 6 Department of Cell Biology, Duke University, Durham, North Carolina 27710, USA; 7 Department of Ophthalmology, Duke University, Durham, North Carolina 27710, USA; 8 Department of Child Health Care, The Children's Hospital of Fudan University, 399 Wanyuan Road, Shanghai 201102, China; 9 Department of Cell Biology and Physiology, The University of North Carolina at Chapel Hill, North Carolina 27599, USA; 10 Duke Institute for Brain Sciences, Duke University, Durham, North Carolina 27710, USA; 11 University Program in Genetics and Genomics, Duke University, Durham, North Carolina 27710, USA

## Abstract

Human neuroimaging studies suggest that aberrant neural connectivity underlies behavioural deficits in autism spectrum disorders (ASDs), but the molecular and neural circuit mechanisms underlying ASDs remain elusive. Here, we describe a complete knockout mouse model of the autism-associated *Shank3* gene, with a deletion of exons 4–22 (Δe4–22). Both mGluR5-Homer scaffolds and mGluR5-mediated signalling are selectively altered in striatal neurons. These changes are associated with perturbed function at striatal synapses, abnormal brain morphology, aberrant structural connectivity and ASD-like behaviour. *In vivo* recording reveals that the cortico-striatal-thalamic circuit is tonically hyperactive in mutants, but becomes hypoactive during social behaviour. Manipulation of mGluR5 activity attenuates excessive grooming and instrumental learning differentially, and rescues impaired striatal synaptic plasticity in Δe4–22^−/−^ mice. These findings show that deficiency of Shank3 can impair mGluR5-Homer scaffolding, resulting in cortico-striatal circuit abnormalities that underlie deficits in learning and ASD-like behaviours. These data suggest causal links between genetic, molecular, and circuit mechanisms underlying the pathophysiology of ASDs.

## Abstract (web summary)

SHANK3 mutations have been linked to autism spectrum disorders, although the underlying mechanisms remain unclear. Here, the authors generate a complete knockout Shank3 mouse model, identifying ASD-like behaviours associated with impaired mGluR5-Homer scaffolding and abnormal brain connectivity.

## Graphical abstract

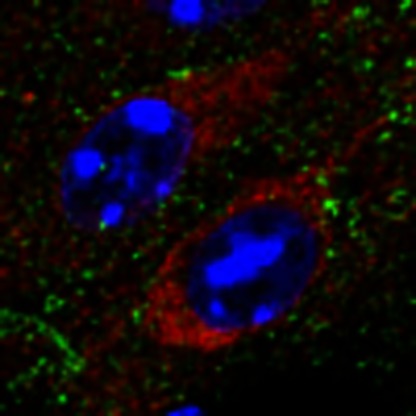

Despite significant progress in identifying genetic defects in autism spectrum disorders (ASDs), the molecular and neural circuit mechanisms that underlie the behavioural impairments remain poorly defined. Genetic studies have consistently identified mutations in genes implicated in synaptic development and function^1^,^2^, although no clear consensus has emerged regarding the specific synapse types or brain regions whose dysfunction underlies ASDs. Recent studies suggest that the pathophysiology of ASDs involves not only aberrant synaptic connections but also defective development of neural networks and abnormal neural synchronization^3^,^4^. Neuroimaging investigations indicate that ASDs are associated with perturbed neural connectivity^5^; however, its exact nature remains uncertain^6^,^7^. Early studies identified reduced functional connectivity^8^, whereas recent reports implicate hyper-connectivity in multiple brain regions^9^,^10^. Further limiting their interpretability, these studies were conducted primarily in high-functioning ASD patients for whom etiologies are mostly unknown.

Mouse models can provide unique insights into the basic biological mechanisms underlying ASDs, but the development of these models is challenging because the biological basis for the majority of ASDs remains unknown^11^. Moreover, most animal models lack strong construct validity supported by human genetic studies^12^. *SHANK3(PROSAP2)*-related mutations represent a unique opportunity to address this challenge^13^. Genetic defects of *SHANK3* are one of the best replicated findings in autism genetics^14^,^15^,^16^. Point mutations typically lead to limited disruption of isoform-specific expression of *SHANK3*, due to multiple intragenic promoters and alternatively spliced coding exons within the gene^17^. However, the vast majority of *SHANK3* mutations found in ASDs are deletions of the entire gene. Most patients carrying deletions of the entire *SHANK3* gene in 22q13.3 deletion or Phelan-McDermid syndrome (PMS) have the diagnosis of ASDs^16^,^18^,^19^.

Eleven lines of *Shank3* isoform-specific mutant mice, with deletions of different exons or point mutations [Δe4–7, Δe4–9 (two lines), Δe9, Δe11, Δe13–16, Δe21 (two lines) e21^InsG3680^ (two lines) and e21^R1117X^], have been reported^20^,^21^,^22^,^23^,^24^,^25^,^26^,^27^,^28^. These *Shank3* mice show variable molecular, synaptic and behavioural phenotypes, likely because different sets of *Shank3* isoforms were disrupted in each line. While these data support heterogeneity in the phenotypes of *Shank3* mutations, most of them lack construct validity because the same exonic deletions have not been reported in humans.

Accordingly, we generated *Shank3* complete knockout mice, by deleting the protein-coding exons 4–22 (Δe4–22). Here we present results from molecular, ultrastructural and electrophysiological analyses, high-resolution magnetic resonance histology (MRH), diffusion tensor imaging (DTI) (structural connectomics), *in vivo* multi-circuit mapping (functional connectomics), behavioural testing and pharmacological analyses of the Δe4–22 mice. Together, these data support the significance of these mice as a particularly suitable model for *SHANK3*-related human neurodevelopmental and neuropsychiatric disorders.

## Results

### Generation of *Shank3* complete knockout mice

**Figure 1:**
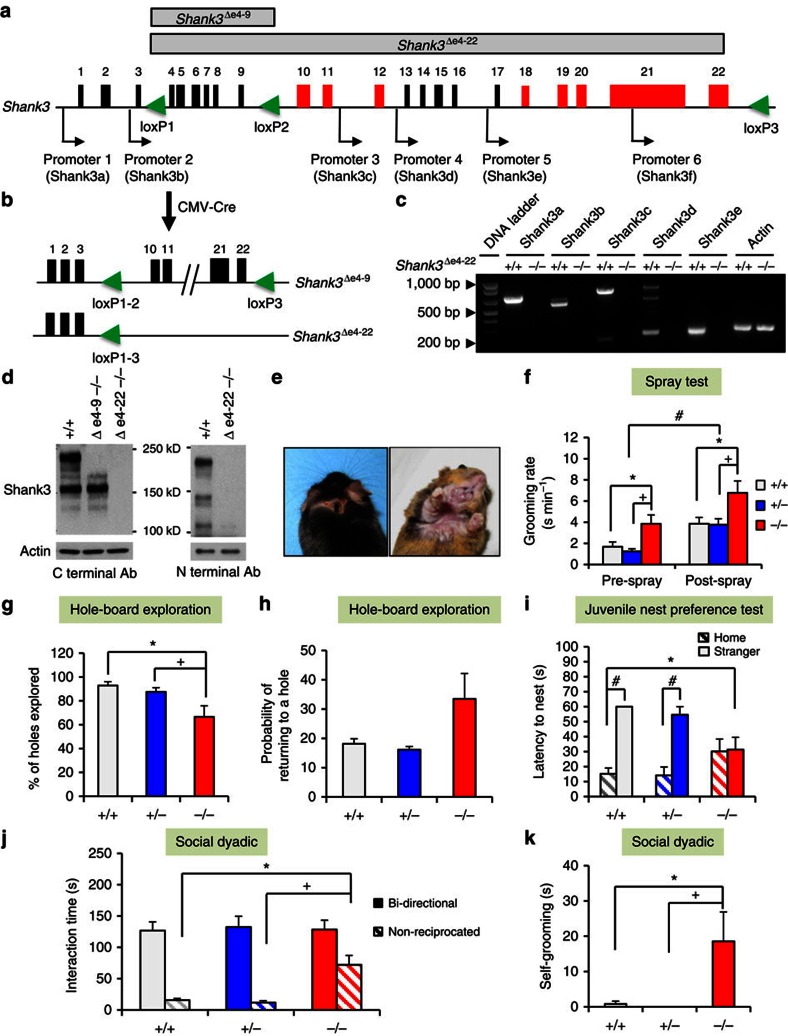
Generation of *Shank3* complete knockout mice and their ASD-like behaviours. (**a**) Schematic design for *Shank3* complete knockout mice using a Cre-*loxP* strategy. Alternatively spliced exons are in red and promoters are indicated by arrows. *loxP* sites are green triangles. (**b**) e4–22^floxed^ mice were crossed with CMV-Cre mice to generate deletion of Δe4–9 or Δe4–22, respectively. (**c**) All mRNA isoforms of *Shank3* were deleted in Δe4–22^−/−^ (−/−) mice relative to Δe4–22^+/+^ (+/+) mice, as shown by RT–PCR. (**d**) Western blot shows that all Shank3 protein bands are absent in −/− brain, using Shank3 C- and N-terminal antibodies. The experiments were repeated three times. (**e**) Skin lesions were observed in >50% of −/− mice, but not in +/+ or Δe4–22^+/−^ (+/−) mice (*χ*^2^_(2,*N*=89)_=38.4, *P*<0.001). (**f**) −/− mice spent significantly more time in self-grooming (RMANOVA: genotype F_(2,46)_=5.68, *P*<0.01), relative to +/+ and +/− mice (*ps*<0.01), *n*=15–18/genotype. (**g**,**h**) Hole-board exploration. (**g**) On the hole-board, −/− mice explored fewer holes (*p*s<0.05) than the other genotypes (F_(2,21)_=5.64, *P*<0.02) (**h**) with the −/− mice showing a trend for increased probability of re-investigating holes (F_(2,21)_=2.99, *P*<0.08); *n*=7–8/genotype. (**i**) −/− pups (P15) failed to demonstrate a preference for their home over a stranger's nest, whereas +/+ and −/− littermates preferred and rapidly entered (*p*s<0.001) their home nest (RMANOVA: nest-choice × genotype F_(2,25)_=4.38, *P*<0.03); *n*=8–10/genotype. (**j**,**k**) Responses in the social dyadic test. (**j**) No genotype differences were detected in bidirectional contact in the social dyadic test. However, the duration of non-reciprocated interaction was prolonged (*ps*<0.001) in −/− mice (F_(2,37)_=11.30, *P*<0.001); *n*=10–15/genotype. (**k**) During the social dyadic test, −/− mice engaged in more self-grooming (F_(2,37)_=4.99, *P*<0.02) than the other genotypes (*p*s<0.02); *n*=10–15/genotype. For all panels, *P<0.05 from +/+; ^+^*P*<0.05 from +/−; ^#^*P*<0.05, within genotype for *post hoc* comparisons. All data are expressed as means±s.e.m. RT–PCR, PCR with reverse transcription.

We and others have reported several isoform-specific *Shank3* mutant mice (Supplementary Fig. 1a). To disrupt all murine *Shank3* isoforms, we adopted a two-step gene targeting and a Cre/*loxP* strategy to flox exons 4–22 (e4–22^floxed^); these exons span 58 kb and include the coding sequences for all Shank3 protein isoforms (Fig. 1a and Supplementary Fig. 1b–e). E4–22^floxed^ mice were bred with CMV-Cre mice to generate mice with deletions of exons 4–22 (Δe4–22) (Fig. 1b). Loss of all known *Shank3* mRNA and protein isoforms in Δe4–22^−/−^ mice was confirmed (Fig. 1c,d and Supplementary Fig. 1f).

After backcrossing to the C57BL/6J strain for more than eight generations, 10 cohorts of Δe4–22 mice were used for behavioural testing (Supplementary Table 1). Δe4–22 mice were viable, without any gross developmental defects, although ear opening and paw position developmental milestones in Δe4–22^−/−^ pups on postnatal day 4 (P4) were delayed (Supplementary Table 2). The body weights of Δe4–22^−/−^ mice were similar to the other genotypes regardless of sex (Supplementary Fig. 1g), and we did not observe spontaneous seizures in these mice. No genotype differences were found in preference for sniffing a social stimulus (Supplementary Table 2). Both genotypes demonstrated habituation-dishabituation to olfactory stimuli; however, responses were less robust for Δe4–22^−/−^ mice (Supplementary Fig. 1h).

### Δe4–22^−/−^ mice display core behavioural features of ASDs

More than half of Δe4–22^−/−^ mice (16/30) developed skin lesions by 5½ months of age (range: 4–7 months) across three cohorts; no such lesions were seen in Δe4–22^+/−^ (0/30) or Δe4–22^+/+^ (0/29) mice. Lesions were observed around the eyes, on the ears, back of the head, and under the chin of mice on both C57BL/6J and mixed C57BL/6J-129R1 backgrounds (Fig. 1e). These lesions appeared to be caused by excessive self-grooming (Supplementary Movie 1). To examine this propensity in Δe4–22^−/−^ mice that had not developed lesions, we evaluated grooming in the home cage before and after misting with water. Although all mice increased grooming after spraying, the Δe4–22^−/−^ mice engaged in significantly higher rates of grooming, relative to Δe4–22^+/−^ and Δe4–22^+/+^ animals (Fig. 1f).

To examine another aspect of repetitive behaviour, mice were tested on the hole-board. While Δe4–22^−/−^ and Δe4–22^+/−^ mice engaged in fewer nose-pokes than Δe4–22^+/+^ mice, these differences were not significant (Supplementary Table 2). However, Δe4–22^−/−^ mice engaged significantly fewer of the 16 holes (Fig. 1g) than the other genotypes; they also had a greater tendency to re-investigate the same hole (Fig. 1h). Collectively, we find that Δe4–22^−/−^ mice display more repetitive behaviours and they sample their environment in a more restricted pattern than Δe4–22^+/−^ and Δe4–22^+/+^ mice.

To evaluate social preferences in neonates, we examined Δe4–22 juveniles (P15) in a nest-homing test. In a one-way test, the latency to approach a sample of the home nest did not differ among the genotypes (Supplementary Table 2). However, when given a choice between the home nest and an unfamiliar nest sample from a developmentally matched litter's nest, the Δe4–22^−/−^mice failed to show a preference for home nests, unlike Δe4–22^+/−^ and Δe4–22^+/+^ mice (Fig. 1i). In a further examination of social behaviour, we monitored the formation of dominance hierarchies over 6 days where three unfamiliar adults of the same genotype were placed into each cage. Since no sex differences were found (Supplementary Table 2), the data were collapsed across sex. From the number and direction of agonistic behaviours observed within each triad, a dominance ranking was calculated for each mouse. All Δe4–22^+/+^ mice established a hierarchy rank by day 1 and this remained stable through day 6. In contrast, 75% of hierarchies of Δe4–22^−/−^ mice were unstable; either dominance was not maintained across days, or no dominant animal could be identified in the triad (*χ*^2^_(1, *N*=30)_=19.3, *P*<0.001).

Mice were next evaluated for sociability. Similar levels of preference for the novel social stimuli in the social affiliation and social preference tests were displayed by the three genotypes (Supplementary Fig. 2a); no genotype differences were discerned in the duration or numbers of contacts with the non-social and social stimuli across all test phases (Supplementary Table 2). To evaluate social behaviour in a novel environment more critically, we conducted social dyadic tests. In pairings of Δe4–22 males with age-matched C3H males, the time spent in and the numbers of bidirectional interactions did not differ among the genotypes; the mice only displayed mild social investigations characterized by approach behaviours and sniffing of the head, face, anogenital and shoulder areas of the partner mouse (Fig. 1j and Supplementary Table 2). However, Δe4–22^−/−^ mice engaged in longer and greater numbers of non-reciprocated behaviours (where the *Shank3* mouse initiated the social interaction, but the C3H partner did not reciprocate and disengaged its partner by ignoring, leaving, or turning away from the target mouse) than the other genotypes (Fig. 1j and Supplementary Table 2). Moreover, Δe4–22^−/−^ animals engaged in more self-grooming during the social test than did Δe4–22^+/+^ or Δe4–22^+/−^ mice (Fig. 1k). Thus the Δe4–22^−/−^ mice have normal levels of social interest, but they persist in unsuccessful efforts to engage the social partner, and they engage in non-social behaviours that include repetitive self-grooming behaviours during social encounters.

**Figure 2:**
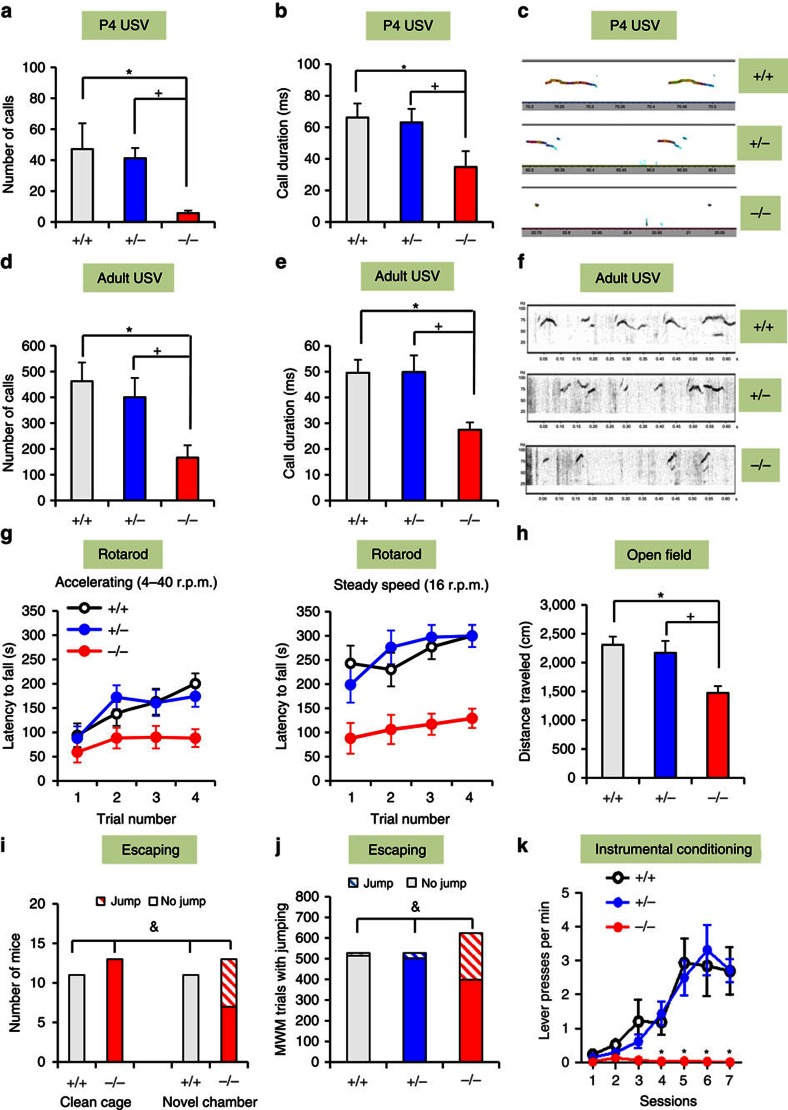
Δe4–22^−/−^ mice display additional ASDs-related and comorbid behaviours. (**a**–**f**) Abnormal USV communication. P4 Δe4–22^−/−^ (−/−) pups emitted significantly fewer USVs (**a**) (F_(2,32)_=7.29, *P*<0.003) and of shorter duration (**b**) (F_(2,29)_=4.59, *P*<0.02), than the other genotypes (*p*s<0.01). (**c**) Representative spectrographs of P4 USVs; *n*=8–13/genotype. Upon exposure to females, adult −/− males emitted fewer USVs (**d**) (F_(2,38)_=6.15, *P*<0.01) that were of significantly shorter duration (**e**) (F_(2,34)_=7.12, *P*<0.01) than the other genotypes (*p*s<0.02). (**f**) Representative spectrographs of adult USVs; *n*=10–18/genotype. (**g**,**h**) Impaired motor performance. (**g**) On the accelerating rotarod (left) −/− mice had shorter latencies to fall (RMANOVA: genotype effect F_(2,27)_=5.33, *P*<0.02) than the other genotypes (*p*s<0.01). On the steady-speed rotarod (right), −/− mice also fell sooner (RMANOVA: genotype effect F_(2,27)_=15.86, *P*<0.001) than the other genotypes (*p*s<0.005); *n*=9–12/genotype. (**h**) Hypoactivity in the open field. −/− mice had reduced locomotion (F_(2,23)_=6.04, *P*<0.01) relative to the other genotypes (*p*s<0.001); *n*=6–10/genotype. (**i**,**j**) Escape behaviours in different environments. (**i**) No mice escaped from new home cages. However, six out of 13 −/− mice escaped from a novel environment (*χ*^2^_(1, N=24)_=6.77, *P*<0.01); *n*=11–13/genotype. (**j**) In the MWM, −/− mice escaped from the hidden platform on significantly more trials than the other genotypes (*χ*^2^_(2, *N*=1,680)_=314.01, *P*<0.001); *n*=11–13/genotype. (**k**) Impaired instrumental learning. −/− mice had difficulty learning to press a lever for food reward (RMANOVA: genotype effect F_(2,102)_=4.7, *P*<0.05, genotype × session F_(12, 102)_=3.0, *P*<0.001) relative to the other genotypes (*p*s<0.001); *n*=4–8/genotype. For all panels, *P<0.05 from +/+, ^+^*P*<0.05 from +/− for *post hoc* comparisons; ^&^*P*<0.05 for *χ*^2^ analyses. All data are expressed as means±s.e.m. MWM, Morris water maze.

Ultrasonic vocalizations (USVs) were assessed in both pups and adults, as communication impairments represent a major feature of *SHANK3*-related disorders and ASDs. The P4 Δe4–22^−/−^ pups emitted significantly fewer USVs that were of shorter duration than Δe4–22^+/−^ and Δe4–22^+/+^ pups (Fig. 2a–c). Although the frequencies and amplitudes of calls from Δe4–22^−/−^ mice were lower than the other two genotypes (Supplementary Fig. 2b,c), all USVs were within a range appropriate for pup distress calls^29^. Upon exposure to oestrous females, Δe4–22^−/−^ adult males emitted fewer calls, and their calls were significantly shorter in duration and reduced in amplitude relative to the other genotypes (Fig. 2d–f and Supplementary Table 2). By comparison, no genotype differences were observed in the peak frequency of adult USVs (Supplementary Table 2).

### Δe4–22^−/−^ comorbidities phenocopy *SHANK3* patients

Motor performance in Δe4–22^−/−^ mice was deficient on both the accelerating and steady-speed rotarod tasks (Fig. 2g), despite normal grip strength (Supplementary Table 2). In their home cages, we observed that Δe4–22^−/−^ mice were hypoactive. This response was confirmed in the open field, where they showed reduced locomotion (Fig. 2h) and a tendency towards decreased rearing (Supplementary Table 2). Δe4–22^−/−^ mice also displayed reduced locomotion and spent less time in the centre of the open field compared with the other genotypes (Supplementary Fig. 2d and Supplementary Table 2). In the light–dark emergence test, Δe4–22^−/−^ mice delayed their entry into the lighted chamber (Supplementary Fig. 2e) and made fewer transitions between chambers than the other genotypes (Supplementary Fig. 2f). Collectively, these results suggest that Δe4–22^−/−^ mice present with anxiety-like behaviours.

While habituating mice to arenas for the novel object recognition memory test, many Δe4–22^−/−^ mice tried to escape from the test chamber. Since we did not observe escape responses in their home cages, we hypothesized that novelty was inducing this behaviour. When mice were placed individually into clean home cages, no escape behaviour was observed (Fig. 2i). However, when the mice were transferred to a novel larger arena, one-half of the Δe4–22^−/−^ mice escaped; no Δe4–22^+/+^ mice engaged in this response. Similarly, during testing in the Morris water maze, we found that Δe4–22^−/−^ mice leapt from the hidden platform and away from the experimenter on more trials than the other genotypes (Fig. 2j). This behaviour may be similar to the enhanced reactivity to novel environments reported in *SHANK3*-related ASD patients^15^,^18^,^19^.

Cognitive performance was examined using several different paradigms. Pre-attentive function was unchanged when analysed by prepulse inhibition, but startle reactivity was reduced in Δe4–22^−/−^ compared with the Δe4–22^+/+^ mice (Supplementary Table 2). Hippocampal function was evaluated with the Morris water maze. Δe4–22^−/−^ mice showed normal acquisition performance (Supplementary Fig. 2g), but displayed slower reversal learning when the hidden platform was moved from the northeast to the southwest quadrant (Supplementary Fig. 2h). During probe trials, Δe4–22^−/−^ mice were impaired in locating the target quadrant, compared with the other genotypes (Supplementary Fig. 2i–k). In the visible platform task, swim time did not differ among genotypes (Supplementary Table 2), indicating that sensory and motor function and motivation were intact. In fear conditioning, no significant genotype differences were noted for cued conditioning, although Δe4–22^−/−^ mice showed a small enhancement in freezing during contextual testing (Supplementary Table 2). Since Shank3 is enriched in the striatum (Supplementary Fig. 6a), Δe4–22 mice were examined in a striatal-dependent instrumental learning task^30^. Each bar-press was reinforced with a food pellet across seven sessions. The Δe4–22^−/−^ mice were unable to learn this task, showing a dramatic deficit after the second training session (Fig. 2k). In summary, we find that fear learning is intact in Δe4–22^−/−^ mice, whereas hippocampal spatial memory is mildly perturbed, and striatal learning is severely impaired.

### Altered functional connectivity in a frontostriatal circuit

**Figure 3:**
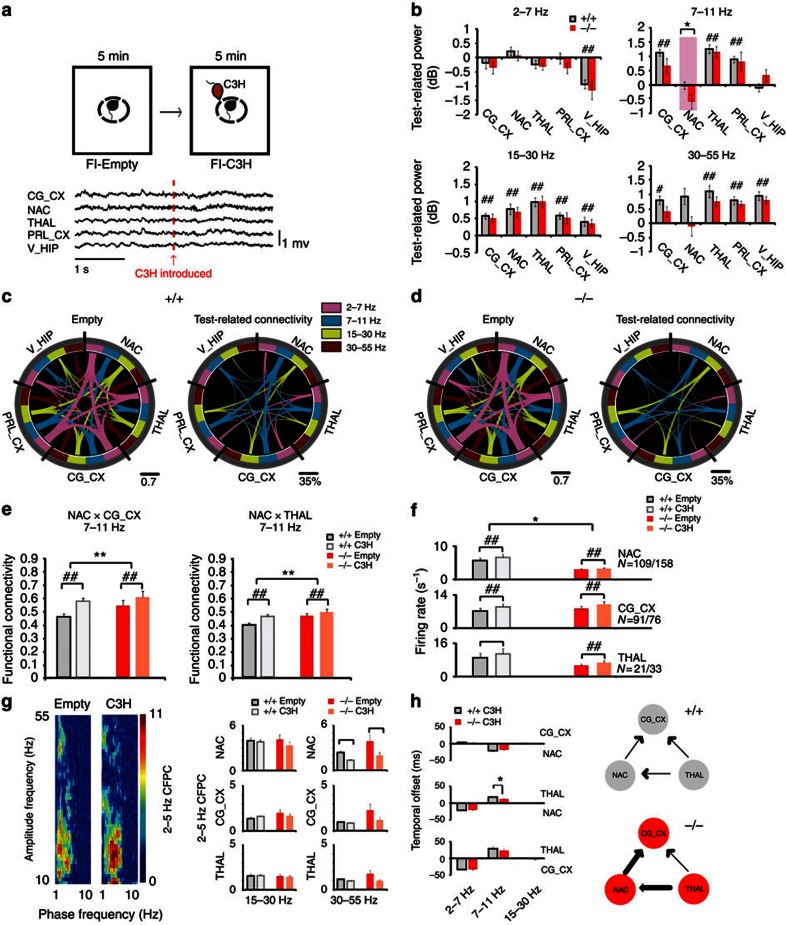
Aberrant functional neural connectivity in Δe4–22 mice. (**a**) Schematic representation of the forced social interaction test (top). A 4 s trace of LFP activity in Δe4–22 mice, recorded before and following introduction of the C3H social stimulus mouse (arrow at bottom). (**b**) Test-related changes in oscillatory power in Δe4–22^−/−^ (−/−) and Δe4–22^+/+^ (+/+) littermates. (Mixed model ANOVA genotype × test–condition interaction (see Supplementary Table 3)). *P< 0.05, +/+ versus −/− mice, ^#^*P*<0.05, comparisons within genotype. (**c**,**d**) Diagrams showing functional connectivity across the recorded brain areas before and after introduction of the social stimulus mouse in +/+ (**c**) and −/− (**d**) mice. The thickness of the connecting lines corresponds to the coherence between each pair of brain areas in the specified frequency band. The image on the right shows the change in coherence observed across the network following introduction of the social stimulus mouse. Only coherence measures that demonstrated significant test–condition interactions are shown. (**e**) Significant genotype by test–condition interaction identified in the social-related network using mixed model ANOVA. **P<0.05, for significant genotype effect, +/+ versus −/− mice (FDR-corrected); ^##^*P*< 0.01, within genotype comparison (FDR-corrected) (see Supplementary Table 3). (**f**) Effects of genotype and test–conditions—in unit firing rates (mixed model ANOVA, genotype × test–condition interaction (see Supplementary Table 3)). *P< 0.05, for significant genotype effect, +/+ versus −/− mice; ^##^*P*<0.05, for test–condition effects within genotype. (**g**) LFP phase-amplitude coupling (CFPC) in +/+ and −/− mice. LFP-β (15–30 Hz) and -γ (30–55 Hz) oscillatory activities were modulated by the phase of locally recorded 2–5 Hz oscillations. An example of CFPC across a single NAC channel is shown during the forced interaction test (left). Effects of genotype and test–conditions in CFPC (right) (mixed model ANOVA, genotype × test–condition interaction (see Supplementary Table 3)). ^##^*P*<0.05, for test–condition effects within genotype. (**h**) Directional network communication during social interaction. A temporal delay between thalamic to striatal oscillatory activity was diminished in the −/− animals. *P<0.05, −/− versus +/+ (FDR-corrected rank sum test). The thickness of a line in the panel to the right denotes the degree of functional connectivity. For all panels, *n*=11–14/genotype. All data are expressed as means±s.e.m.

Alterations in brain oscillatory function and global network connectivity have been proposed to mediate ASDs^7^,^31^. To test whether *Shank3* disruption was sufficient to alter oscillatory activity across limbic networks, Δe4–22^+/+^ and Δe4–22^−/−^ mice were implanted with microwire arrays into the prelimbic cortex (PRL_CX), cingulate cortex (CG_CX), medial dorsal nucleus of thalamus (THAL), ventral hippocampus (V_HIP), and nucleus accumbens (NAC). The NAC was targeted for *in vivo* analysis instead of the dorsolateral striatum (DLS) because the NAC is the projection target of the CG_CX, and this latter brain region is heavily involved in social behaviours^32^. Following surgical recovery, local field potentials (LFP) and single-unit activities were recorded before and after exposure to a novel mouse as a test for social responsiveness (Fig. 3a).

Significant changes in oscillatory power were observed across many brain areas upon introduction of a social stimulus to both genotypes. Lower NAC oscillatory power in the 7–11 Hz band was observed in Δe4–22^−/−^ mice, both before and after exposure to the social stimulus (Fig. 3b and Supplementary Table 3). Exposure to the novel mouse increased coherence (reflecting increased functional connectivity) throughout the limbic network in Δe4–22^+/+^ controls (Fig. 3c and Supplementary Table 4), especially in the 7–11 Hz band, representing a network previously proposed to mediate behavioural deficits in ASDs^31^. In contrast, Δe4–22^−/−^ mice displayed diminished functional activation (that is, a smaller task-induced increase in coherence) in the 7–11 Hz network across the cingulate cortico-striatal-thalamic axis (Fig. 3d,e). Other elements of the 7–11 Hz network (the hippocampal-striatal and cingulate cortico-thalamic circuits) may also have been affected, though these differences did not reach statistical significance (Supplementary Table 3). Likely contributing to these changes, NAC-dependent functional connectivity before the introduction of the novel mouse tended to be higher in Δe4–22^−/−^ than in Δe4–22^+/+^ mice. We conclude that *Shank3* deficiency results in hyperactivation of this social neural network under basal conditions, whereas the gain induced by the social stimulus is diminished in Δe4–22^−/−^ mice.

Aside from abnormal network activity, Δe4–22^−/−^ mice also displayed lower unit firing rates in NAC both before and after introduction of the social stimulus (Fig. 3f). No genotype differences were observed in cross-frequency phase coupling or phase lock of unit responses (two measures of local connectivity) within the cortical, striatal and thalamic microcircuits (Fig. 3g and Supplementary Fig. 3). We quantified directional oscillatory interactions during the social stimulus presentation, as a measure of information transfer across the cortico-striatal-thalamic circuit. In both Δe4–22^−/−^ and Δe4–22^+/+^ mice, oscillatory synchrony was directed from thalamus to cortex and striatum in the 7–11 Hz frequency band; however, the temporal delay between thalamic to striatal oscillatory activity was diminished in the Δe4–22^−/−^ animals (Fig. 3h).

### NAC firing deficit is due to local *Shank3* disruption

The NAC firing deficit observed in the Δe4–22^−/−^ mice could result from developmental influences of *Shank3* disruption on NAC function, changes in the function of NAC inputs from other brain regions (for example, hippocampus), or a direct change in the firing properties of NAC independent of development. To examine directly these possibilities, we used e4–22^floxed^ mice (Fig. 1a). We bilaterally infected the NAC of these e4–22^floxed^ mice with an AAV10-Cre virus to selectively knockdown Shank3 in the NAC; an AAV10-GFP virus served as the control (Supplementary Fig. 4a). Four weeks after injections, these mice were surgically implanted with recording electrodes in NAC and unit activity was recorded during the baseline period of the social stimulus probe test. The reduced Shank3 expression, locations of viral infection (Supplementary Fig. 4b) and NAC electrode placement (Supplementary Fig. 4c) were verified *post mortem*. We found that the mice treated with AAV10-Cre exhibited significantly lower firing rates in the NAC than AAV10-GFP controls (Supplementary Fig. 4d). Thus, these results provide direct evidence that selective deficiency of *Shank3* in the NAC is sufficient to reduce the firing rate of medium spiny neurons (MSNs), recapitulating the phenotype observed in the Δe4–22^−/−^ mutants.

### Altered brain morphometry and structural connectivity

**Figure 4:**
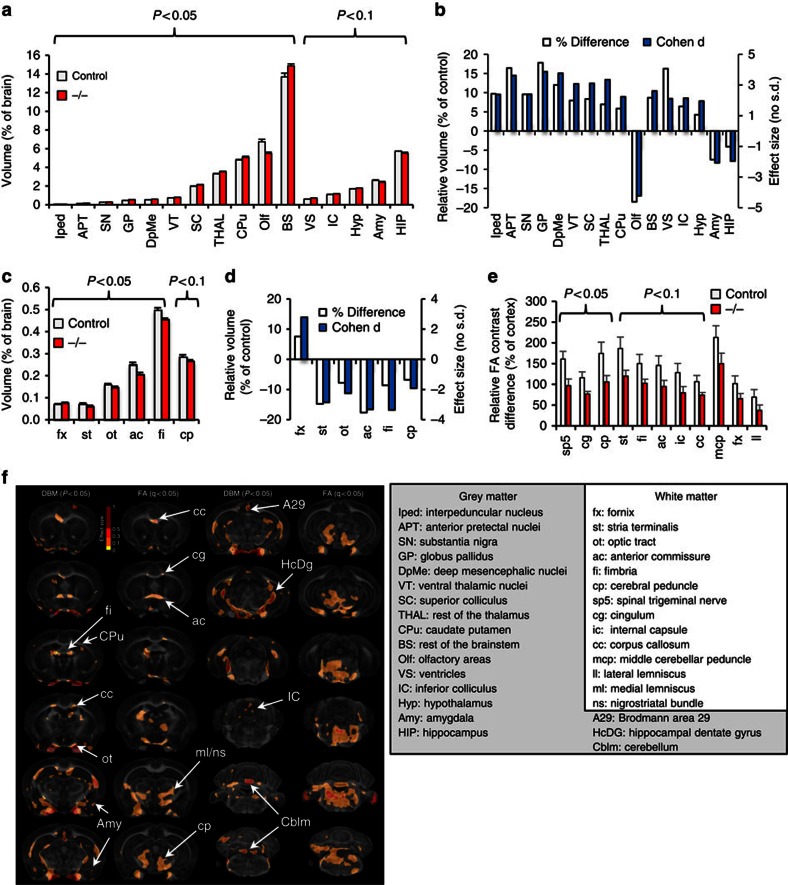
Abnormalities in brain structure in Δe4–22^−/−^ mice. (**a**,**b**) Volume changes in grey matter of Δe4–22 mice. (**a**) Significant volume differences in several brain regions (normalized to total brain volume) were observed in Δe4–22^−/−^ (−/−) mice, relative to controls. *t*-test, *n*=7 mice/genotype. (**b**) These differences are also apparent when the data are expressed as relative volume (shown as percentage of control) or Cohen's d changes, where positive values represent enlargement and negative values represent reduction in −/− mice. (**c**,**d**) Volume changes were observed in white matter tracts. *t*-test, *n*=7 mice/genotype. (**e**) Changes in diffusion tensor imaging (DTI) parameters, computed relative to the cortex fractional anisotropy (FA), 100*(ROI FA-cortex FA)/cortex FA. Reduced FA values were observed in white matter tracts in −/− mice relative to controls, accompanied by a 20% average reduction in FA values. *t*-test, *n*=6 mice/genotype. (**f**, left) Statistical parametric maps based on Jacobians and FA pin-point localized changes (arrows) in volume and microstructural organization in −/− mice. While the patterns are distinct, the anterior commissure (ac) and corpus callosum (cc) emerge as common biomarkers that change in both volume and FA. (right) Full names for abbreviations in (**a**–**f**). All data are expressed as means±s.e.m. ROI, regions of interest.

To probe anatomic changes that might underlie the observed impairments in behaviour and functional connectivity, we imaged fixed brains of seven adult Δe4–22^−/−^ and seven C57BL/6J controls, using high-field MRH and a high-resolution (43 μm) DTI protocol^33^,^34^. Quantitative analysis revealed slightly larger brains in Δe4–22^−/−^ mice (472.5±19.7 mm^3^; M±s.e.m.) than in controls (459.7±15.7 mm^3^); however, this did not reach statistical significance. After normalizing regional to total brain volume, multiple grey matter structures were found to differ between Δe4–22^−/−^ and control mice (Fig. 4a,b and Supplementary Table 5). The basal ganglia (globus pallidus, 17.8%; substantia nigra, 9.6%; and caudate putamen, 5.9%) and thalamus (ventral thalamic nuclei, 8.7%; rest of the thalamus, 7.0%) were enlarged, while the olfactory areas (−18.5%), hippocampus (−4.1%), and amygdala (−7.7%) were smaller in the Δe4–22^−/−^ mice. With the exception of the fornix, all the other five white matter tracts had reduced volumes relative to controls (Fig. 4c,d).

To gain insight into white matter integrity, we performed DTI, comparing fractional anisotropy (FA) contrast between white matter tracts and the cortex in both genotypes. Qualitative evaluation of FA images showed reduced intensity in white matter relative to grey matter in Δe4–22−/− mice, compared with controls (Supplementary Fig. 5a). Significant reductions were identified for the spinal trigeminal tract, cingulum, and the cerebral peduncle (Fig. 4e and Supplementary Fig. 5b). We further examined how changes in FA contrast (normalized to cortex) correlated across various fibre tracts throughout the brain (Supplementary Fig. 5c). Significant correlations were found for white matter tracts in Δe4–22^−/−^ mice (48 versus 32 correlational pairs in controls).

Voxel-based morphometry confirmed the regional results and revealed additional areas of volume loss in the Δe4–22^−/−^ mice. The effect size is shown as a texture map overlaid on the average template for the control group (Fig. 4f). Corrected *P* values using all brain voxels exceeded the 5% false discovery rate threshold (FDR). Areas exhibiting significant FA reductions (after FDR correction) included the genu of the corpus callosum and cingulum, the anterior commissure, fimbria, optic tract, areas around the medial lemniscus and nigrostriatal bundles, as well as the cerebral peduncle, cerebellum and brainstem. We detected a unilateral change in the internal capsule, implying an asymmetric change in white matter resulting from *Shank3* deficiency.

### Dysfunctional striatal synapses

**Figure 5:**
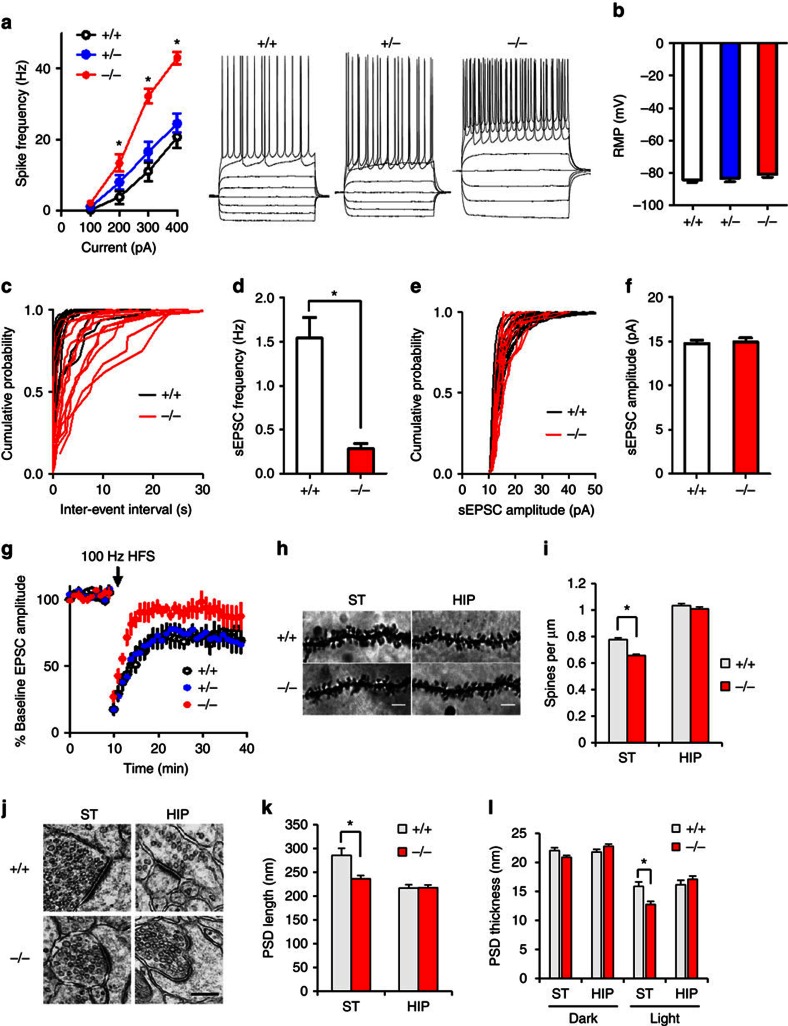
Electrophysiological characterization of striatal medium spiny neurons in Δe4–22 mice. (**a**) Spike frequency in response to current injection. Δe4–22^−/−^ (−/−) neurons showed enhanced excitability with 200–400 pA injected current (*P<0.05) compared with Δe4–22^+/−^ (+/−) and Δe4–22^+/+^ (+/+) neurons (F_(3, 267)_=242.8, *P*<0.0001); *n*=28–32 neurons/genotype. (**b**) No genotype differences in RMPs were observed; *n*=28–32/genotype. (**c**,**d**) The frequency of sEPSCs was significantly reduced in −/− neurons; *P<0.001, *t*-test, *n*=13–14/genotype. (**e**,**f**) The sEPSC amplitude was not altered in −/− neurons; *n*=13–14/genotype. (**g**) HFS-induced LTD was impaired in −/− neurons; (planned comparison, *t*-test, *P*<0.05 for mice +/+, *P*<0.01 for +/−, and *p*>0.05 for −/− mice; *n*=8–12/genotype). (**h**) Sample images of Golgi-impregnated neurons in striatum (ST) and CA1 area of hippocampus (HIP) from −/− and +/+ mice. Scale bar: 5 μm. (**i**) A decrease in spine density was found in striatum of −/− mice. *P<0.001, *t*-test, *n*=97 branches from 50 cells of three mice/genotype. (**j**–**l**) Altered PSD ultrastructure in −/− mice. (**j**) Representative images for EM from striatum and CA1 hippocampus. Scale bar, 0.2 μm. (**k**) The PSD length was significantly decreased in striatum. (**l**) The PSD is thinner in striatum, especially its cytoplasmic ‘light' portion. *P<0.005, *t*-test. *n*=79 synapses from 4 +/+ mice and *n*=139 synapses from 6 −/− mice for striatum; *n*=120 synapses from 6 +/+ mice and *n*=148 synapses from 6 −/− mice for hippocampus. All data are expressed as means±s.e.m. RMP, resting membrane potential.

Our functional and structural connectivity results suggest that striatal circuitry may be dysfunctional in Δe4–22^−/−^ mice. Accordingly, synaptic function was examined in acute striatal slices. We used whole-cell patch-clamp recordings to assess excitatory synaptic transmission in the DLS, which receives strong projections from sensorimotor cortical regions and has been implicated in habit formation and compulsive behaviour^35^. First, we characterized the intrinsic excitability of MSNs by measuring spike frequency in response to depolarizing steps of current injection. Δe4–22^−/−^ MSNs showed enhanced excitability, compared with Δe4–22^+/−^ and Δe4–22^+/+^ neurons (Fig. 5a), while no genotype difference was observed for resting membrane potential (Fig. 5b). Δe4–22^−/−^ mice displayed a marked reduction in the frequency of spontaneous excitatory postsynaptic currents (sEPSCs) (Fig. 5c,d), consistent with decreased spine density and number of glutamatergic synapses on MSNs in these mice. In contrast, the mean sEPSC amplitude was not affected by *Shank3* deficiency (Fig. 5e,f). We used high-frequency stimulation (HFS) -induced long-term depression (LTD) to probe synaptic plasticity. LTD was significantly reduced in Δe4–22^−/−^ mice, compared with the Δe4–22^+/+^ and Δe4–22^+/−^ mice (Fig. 5g). These results indicate that multiple aspects of synaptic function are impaired in Δe4–22^−/−^ striatal neurons.

### Changes in synaptic organization in Δe4–22^−/−^ mice

These abnormalities in synaptic function in Δe4–22^−/−^ striatum led us to investigate possible underlying morphological changes in synapses. Golgi staining revealed that spine density in the striatum of Δe4–22^−/−^ mice was reduced, but not in the hippocampus (Fig. 5h,i). Electron microscopic examination showed that the postsynaptic density (PSD) was significantly shorter and thinner in striatal synapses of Δe4–22^−/−^ compared with Δe4–22^+/+^ mice (Fig. 5j–l). There was a similar trend for smaller PSD structures in hippocampal and cortical synapses (data not shown for cortical synapses).

**Figure 6:**
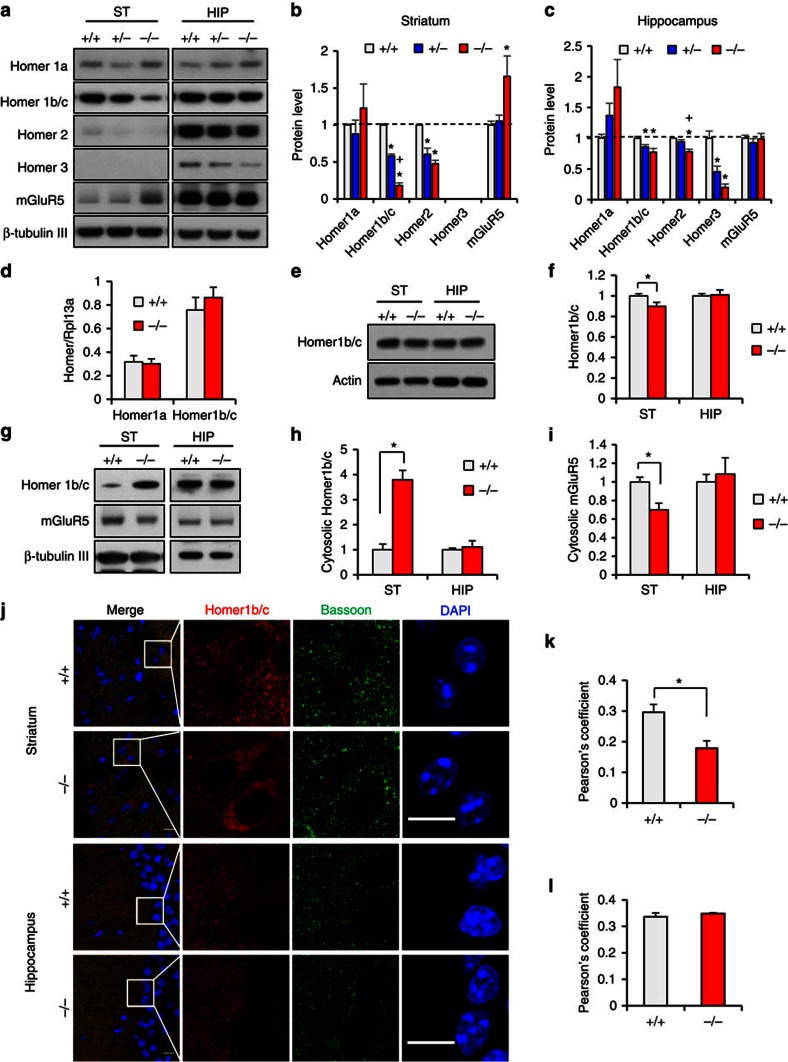
Abnormal Homer and mGluR5 distribution in Δe4–22^−/−^ striatum. (**a**) Altered mGluR5-Homer scaffolds in the PSD fractions from striatum (ST) and hippocampus (HIP) of Δe4–22^−/−^ (−/−) mice. Note a marked decrease of Homer1b/c and increased mGluR5 in −/− striatum. (**b**,**c**) Quantification of Homer and mGluR5 protein in striatum (**b**) and hippocampus (**c**). *P<0.05, −/− versus +/+; ^+^*P*<0.05, −/− versus +/−; one-way ANOVA with Tukey's *post hoc* test; *n*=4/genotype for Homer1b/c, Homer2 and Homer3; *n*=9 for Homer1a; *n*=10 for mGluR5. (**d**) Homer1a and Homer 1b/c mRNA expression was similar in +/+ and −/− mice. *n*=3/genotype. (**e**,**f**) Total Homer1b/c protein was mildly reduced in −/− ST (90% of +/+); *P<0.05, *t*-test, *n*=7/genotype. (**g**) Altered mGluR5 and Homer1b/c in the cytosolic fractions from ST and HIP of −/− mice. (**h**,**i**) Quantification of (**g**) showing increased Homer1b/c and decreased mGluR5 in the cytosol of the striatum of −/− mice; *P<0.01, *t*-test, *n*=4–6/genotype. (**j**–**l**) Immunostaining shows that Homer 1b/c accumulates in the somata of striatal neurons of −/− brain slices (**j**). Co-localization analysis using Pearson's coefficient revealed a decreased correlation between Homer1b/c and Bassoon in −/− striatum (**k**), but not in hippocampus (**l**). *P<0.01, *t*-test, *n*=5–6 /genotype). Scale bar, 10 μm. Experiments for western blots were repeated at least three times. All data are expressed as means±s.e.m.

To analyze the molecular changes that may contribute to dysfunctional synapses, we examined synaptic proteins in PSD fractions from Δe4–22 striata and hippocampi (Supplementary Fig. 6b). Pan-SAPAP and SAPAP3 levels were decreased only in striatum, while GluN2A was reduced only in hippocampus (Supplementary Fig. 6b). The most prominent changes in Δe4–22^−/−^ mice were for the Homer family proteins (Fig. 6a–c). Homer1b/c was markedly decreased (18% of +/+) in striatum and mildly reduced in hippocampus (77% of +/+) relative to Δe4–22^+/+^ mice. There was no significant change for Homer1a in either brain region. In the Δe4–22^−/−^ mice, Homer2 was significantly decreased in both brain areas (48% of +/+ in striatum; and 78% of +/+ in hippocampus). Homer3 (the least abundant isoform) was undetectable in striatum, but was reduced in the Δe4–22^−/−^ hippocampus (20% of +/+). Unexpectedly, we found that mGluR5 was substantially increased in striatum (165% of +/+), but not in Δe4–22^−/−^ hippocampus. These results indicate region-specific alterations in PSD proteins in Δe4–22^−/−^ brains, especially in the striatum.

### Altered cellular distributions of Homer1b/c and mGluR5

We next examined the possible mechanism by which synaptic Homer1b/c was reduced. The mRNA levels for Homer1a and Homer1b/c in Δe4–22^−/−^ striatum and hippocampus were similar to +/+ controls (Fig. 6d). Although total Homer1b/c protein was slightly decreased in Δe4–22^−/−^ striatum (Fig. 6e,f), this is unlikely to explain the drastic reduction of Homer1b/c in the PSD. Sub-fractionation studies revealed a marked increase of Homer1b/c in the cytosolic fraction from Δe4–22^−/−^ striatum (379% of +/+), while that in the hippocampus was similar to controls (Fig. 6g,h). In contrast, striatal cytosolic mGluR5 was decreased (70% of +/+), with no genotype differences in the hippocampus (Fig. 6i). Homer1b/c was also examined in Δe4–9 mice. The decreased expression of Homer1b/c in the PSD and the cytosolic accumulation of this protein in Δe4–9^−/−^ striatum were less prominent than in Δe4–22^−/−^ mice (Supplementary Fig. 6c,d,f,h).

The increase in cytosolic Homer1b/c in Δe4–22^−/−^ striatum was confirmed by Homer1b/c immunostaining of striatal neurons (Fig. 6j). Similar changes were observed in the NAC but not in the hippocampus or neocortex (Supplementary Fig. 6e,g). Conversely, the co-localization of Homer1b/c with the presynaptic marker Bassoon was significantly decreased in Δe4–22^−/−^ striatum, consistent with decreased Homer1b/c in the PSD (Fig. 6k,l). Homer 1b/c immunostaining completely overlapped with the neuronal marker NeuN (Supplementary Fig. 7g), but only partially overlapped in dopamine D1 receptor (D1R) labelled neurons (defined by D1-td-Tomato) (Supplementary Fig. 7h). Since the striatum is composed primarily of MSNs containing either D1Rs or D2Rs, our findings suggest that mGluR5-Homer scaffolds are likely altered in both D1R- and D2R-positive MSNs.

### Altered mGluR5/ Homer1 organization in striatal synapses

**Figure 7:**
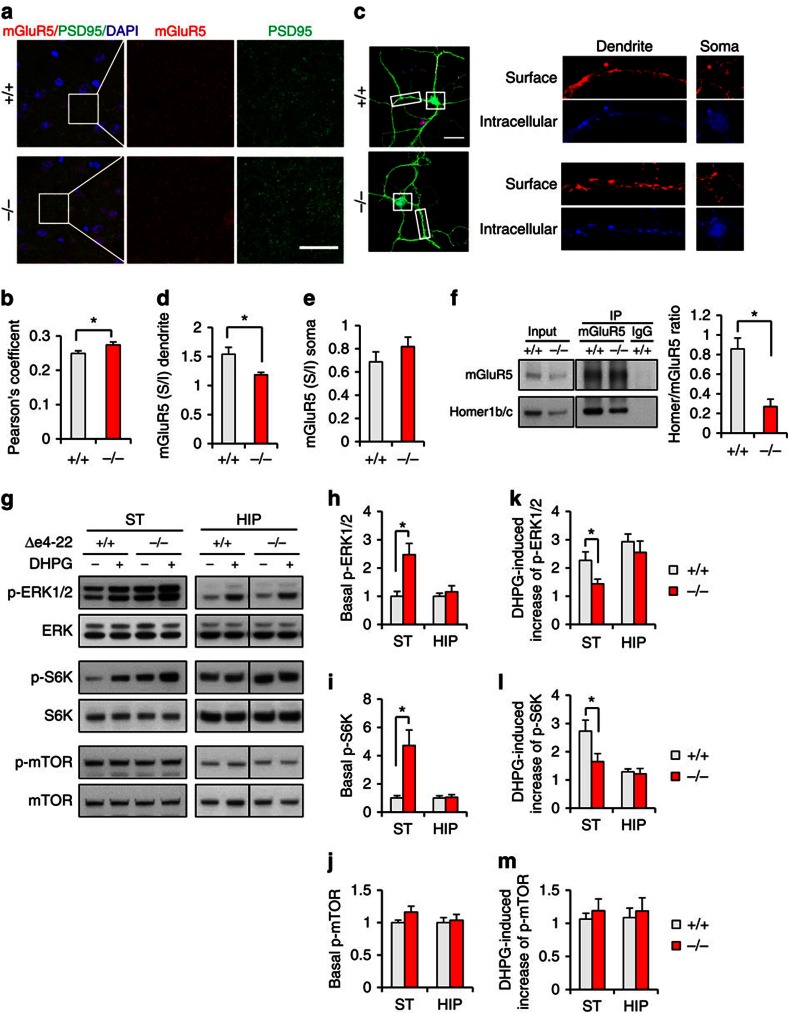
Abnormal accumulation of mGluR5 in PSD and altered mGluR5-Homer1 scaffolds in Δe4–22^−/−^ striatum. (**a**) Immunostaining for mGluR5 (red) and the postsynaptic marker PSD-95 (green) in −/− and +/+ striata. Scale bar, 10 μm. (**b**) Co-localization analysis revealed a higher correlation between mGluR5 and PSD-95 in the striatum of −/− mice; *P<0.05, *t*-test, *n*=24 slices from 3 mice/genotype. (**c**, left) Immunostaining of surface mGluR5 (red), intracellular mGluR5 (blue), and DARPP-32 (green) in dissociated striatal MSNs. (right) Sample images of surface and intracellular mGluR5 immunostaining in dendrites and somata. (**d**,**e**) The ratio of surface/intracellular mGluR5 is decreased in dendrites (**d**), but is not different in somata of MSNs between genotypes; *P<0.01, *t*-test, *n*=14 cells from three mice/genotype. (**f**) Co-immunoprecipitation of mGluR5 and Homer1b/c revealed a decreased association in −/− striatum. *P<0.01, *t*-test, *n*=4 mice/genotype (**g**) Immunoblots show that basal levels of p-ERK1/2 and p-S6K are increased in −/− striatal slices. (**h**–**j**) The basal levels of p-ERK1/2 (**h**) and p-S6K (**i**), but not p-mTOR (**j**) were significantly increased in the striatum (ST) of −/− mice. *P<0.05, two-tailed *t*-test. (**k**–**m**), DHPG had a significant smaller effect on p-ERK1/2 (**k**) and p-S6K (**l**) levels in −/− ST, but the effect on p-mTOR levels (**m**) was similar between genotypes. The net increase of phosphorylation for each kinase was calculated by normalizing DHPG-induced phosphorylation to corresponding basal phosphorylation. *P<0.05, two tailed *t*-test. For ERK, *n*=21 for each genotype in ST, *n*=11 for each genotype in hippocampus (HIP). For S6K, *n*=16 for each genotype in ST, *n*=8 for each genotype in HIP. For mTOR, *n*=19 for each genotype in ST, *n*=8 for each genotype in HIP. Experiments for western blots were repeated at least three times. All data expressed as means±s.e.m.

To corroborate the increased mGluR5 in striatal PSDs, we immune-stained for mGluR5 and PSD-95 in striatal slices. Co-localization studies revealed their increased association in Δe4–22^−/−^ mice (Fig. 7a,b), supporting the biochemical evidence for elevated mGluR5 in synapses. Using an antibody recognizing the extracellular domain of mGluR5, we found that that surface mGluR5s were unchanged in cortical-striatal neuronal co-cultures (Supplementary Fig. 7a–d). However, the ratio of surface to intracellular mGluR5 was reduced in Δe4–22^−/−^ MSN dendrites but not in soma (Fig. 7c–e), suggesting a selectively increased intracellular pool of mGluR5.

The aforementioned mislocalization of Homer1b/c and mGluR5 likely causes disruption of the mGluR5-Homer scaffold, and this is supported by the reduced interaction of Homer1b/c and mGluR5 in Δe4–22^−/−^ striatum (Fig. 7f). To further test if mGluR5-mediated signalling was altered, we examined the phosphorylation state of several kinases in the mGluR5-mediated pathway from Δe4–22^−/−^ striatal and hippocampal slices. (Fig. 7g). Under the baseline condition, levels of p-ERK1/2 and p-S6K were significantly elevated in Δe4–22^−/−^ striatum, but not in hippocampus (Fig. 7h,i). Activation of mGluR5 with the selective group I metabotropic agonist (S)-3,5-dihydroxyphenylglycine (DHPG) enhanced levels of p-ERK1/2 and p-S6K in the striatum in both genotypes. However, the DHPG-induced increase (fold change) of ERK1/2 and S6K phosphorylation was less apparent in Δe4–22^−/−^ striatum (Fig. 7k,l), suggesting a weaker ligand-dependent response for mGluR5. By contrast, there was no genotype difference in p-mTOR level in Δe4–22 striatum and hippocampus before or after DHPG treatment (Fig. 7j,m). Since mGluR5 signalling regulates protein synthesis in hippocampus^36^, we also evaluated protein synthesis in Δe4–22^−/−^ striatum. No genotype difference was observed (Supplementary Fig. 7e,f), though cell type-specific changes cannot be excluded. These results suggest that mGluR5 function may be enhanced at baseline, but the ligand-dependent response of mGluR5 is attenuated in the Δe4–22^−/−^ striatum.

### mGluR5 modulators correct impaired behaviour and LTD

**Figure 8:**
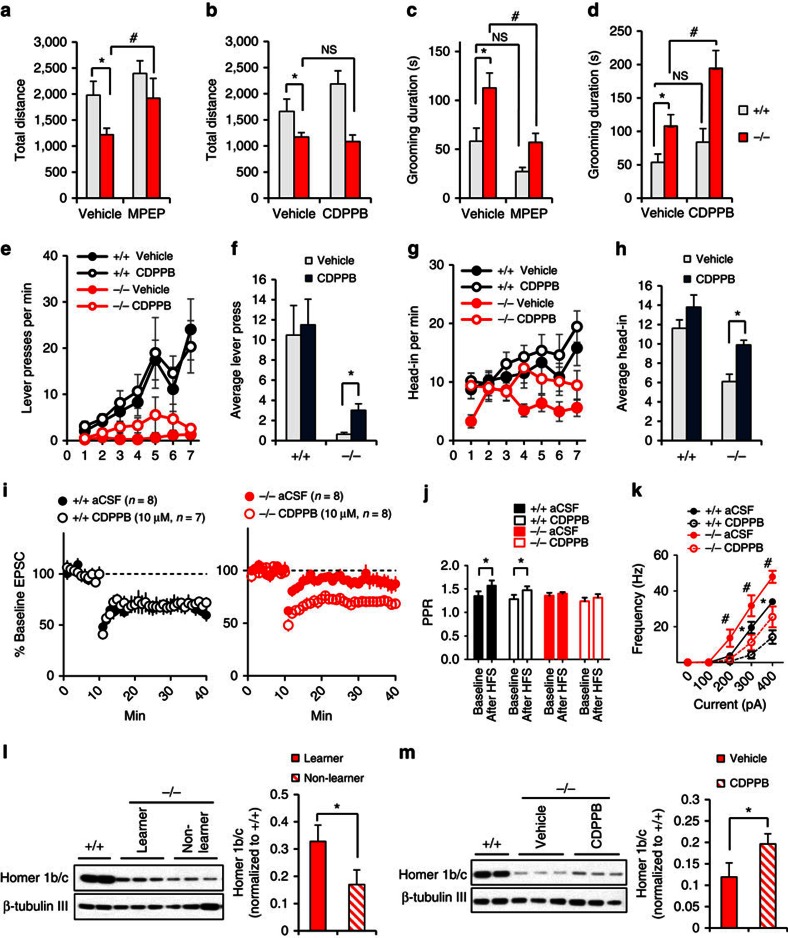
Behavioural phenotypes in Δe4–22 mice are rescued by mGluR5 modulators. (**a**,**b**) Reduced activity in open field in Δe4–22^−/−^ (−/−) mice was rescued by the mGluR5 antagonist MPEP (**a**) but not by the mGluR5 positive allosteric modulator CDPPB (**b**). (**c**,**d**) Increased self-grooming in the −/− mice was attenuated by MPEP (**c**) and was exacerbated by CDPPB (**d**). *P< 0.05, −/− versus +/+; ^#^*P*<0.05, within genotype for treatment. For (**a**,**c**), vehicle: +/+, *n*=10, −/−, *n*=12; MPEP: +/+, *n*=10, −/−, *n*=12. For **b**,**d**, vehicle: +/+, *n*=8, −/−, *n*=12; CDPPB: +/+, *n*=9, −/−, *n*=11. (**e**–**h**) CDPPB enhanced lever pressing (**e**,**f**) and head-entries into the magazine (**g**,**h**) across the 7 days in −/− mice. *P< 0.01, planned comparison: two-tailed *t*-test. vehicle: +/+, *n*=8, −/−, *n*=12; CDPPB: +/+, *n*=9, −/−, *n*=11. (**i**) CDPPB does not alter LTD in MSNs of +/+ mice (left). CDPPB rescues LTD in MSNs of −/− mice (right). (**j**) HFS increased probability of PPR in +/+ mice but not in −/− mice. *P<0.05, paired *t*-test, *n*=7, +/+ CDPPB, *n*=8 for the other groups. (**k**) CDPPB decreased spike frequency in response to current injection in the MSNs of +/+ and −/− mice. *P<0.05, +/+ aCSF versus CDPPB; ^#^*P*<0.05, −/− aCSF versus CDPPB. Bonferroni *post hoc* comparison, *n*=12 each genotype. (**l**) Homer 1b/c protein levels in the striatum are associated with lever press performance. (left) Sample images for western blot. (right) Homer quantification. −/− mice with more than one press/min at last session are defined as ‘learners' and −/− mice with <0.1 press/min are defined as ‘non-learners'. *P<0.05, two-tailed *t*-test. *n*=5 for learners, *n*=7 for non-learners. (**m**) CDPPB increased Homer 1b/c levels in striatum in −/− mice. (left) Sample images for western blot. (right) Homer 1b/c quantification. *P<0.05, two-tailed *t*-test. *n*=9 for vehicle, *n*=8 for CDPPB. All data are expressed as means±s.e.m.

The reduced association of mGluR5 with Homer1b/c suggests that mGluR5-related function may be compromised, whereas the increased mGluR5 in the PSD raises the possibility that mGluR5-mediated functions may be augmented in the Δe4–22^−/−^ striatum. To determine whether these changes may mediate some of the abnormal behaviours in Δe4–22^−/−^ mice, we performed behavioural analysis in mice treated with a mGluR5 antagonist (MPEP) or a positive allosteric modulator of mGluR5 (CDPPB). The hypoactivity in the open field in the Δe4–22^−/−^ mice was normalized with MPEP; this compound also suppressed the increased self-grooming (Fig. 8a,c). In contrast, CDPPB failed to alter the hypoactivity, while it augmented self-grooming in Δe4–22^−/−^ mice (Fig. 8b,d). The inverse effects of MPEP and CDPPB on grooming behaviour support the hypothesis that enhanced mGluR5 function contributes to the elevated self-grooming in Δe4–22^−/−^ mice.

We had found instrumental learning to be profoundly impaired in Δe4–22^−/−^ mice (Fig. 2k). Since there is evidence for reduced mGluR5 function in Δe4–22^−/−^ mice and CDPPB has been reported to rescue impaired learning in *Shank*2 mutant mice^37^, we reasoned that positive allosteric modulation of mGluR5 might restore the impaired instrumental learning. Indeed, we found that CDPPB treatment partially rescued instrumental learning in Δe4–22^−/−^ mice (Fig. 8e–h).

We also tested the effects of CDPPB on synaptic function and neuronal excitability in striatal slices. CDPPB rescued the impaired LTD (Fig. 8i) but it did not change the probability of presynaptic release (PPR) in Δe4–22^−/−^ mice (Fig. 8j). In addition, CDPPB did not change the frequency or amplitude of sEPSCs, and it failed to affect the resting membrane potential (Supplementary Fig. 8a–c). CDPPB also reduced the hyper-excitability in MSNs of Δe4–22^−/−^ mice. However, a similar reduction in excitability was observed in Δe4–22^+/+^ neurons, suggesting that this rescue effect may not be specific (Fig. 8k).

Since Homer1b/c is an important mediator in the mGluR5 signalling pathway, we next examined whether there was any relationship between synaptic Homer1b/c and lever pressing in Δe4–22^−/−^ mice. After 7 days of lever-press training, we found that those mutants with increased numbers of lever presses had significantly higher levels of Homer1b/c in their striatal PSDs than those with lower lever-press rates (Fig. 8l). We also observed that 7 days of treatment with CDPPB resulted in a slight increase of synaptic Homer1b/c in Δe4–22^−/−^ mice (Fig. 8m), consistent with the partial rescue of lever pressing by this compound. These data suggest that the disruption of mGluR5-Homer scaffolds, due to the reduction of Homer1b/c, may contribute to the impaired instrumental learning and striatal plasticity in the Δe4–22^−/−^ mice.

## Discussion

Our complete *Shank3* knockout mouse provides a new genetic model for studying mechanisms underlying ASDs and PMS. Since the most common genetic defect found in human ASDs is deletion of the entire *SHANK3* gene^15^,^18^,^19^, the Δe4–22 mouse has better construct validity for *SHANK3*-related ASDs than previously reported lines of *Shank3* mutant mice^14^,^20^,^21^,^22^,^23^,^24^,^25^,^27^,^28^ (Supplementary Fig. 1a). These mice present with behavioural phenotypes that resemble features of *SHANK3*-related ASDs and PMS^15^,^16^,^18^,^19^ (Supplementary Table 6), such as increased repetitive behaviours, impaired USV communication and aberrant social behaviours, as well as common comorbidities associated with *SHANK3* deficiency. The Δe4–22^+/−^ mice displayed only mild behavioural impairments, in contrast to the severe phenotype in *SHANK3* haploinsufficient patients^15^,^16^,^18^,^19^. The basis for this discrepancy is unknown, but a similar pattern has been seen in other mouse models of human haploinsufficiency^23^,^37^,^38^,^39^.

The Δe4–22^−/−^ mice displayed a spectrum of changes in PSD proteins, synaptic functions and behaviours distinct from previously described lines of *Shank3* mice^23^,^24^,^26^,^27^,^36^,^37^. For example, the isoform-specific Δe4–9 mice^22^ were more impaired in some social tests and in spatial learning and memory, whereas alterations in anxiety/escape behaviours, grooming and striatal Homer1 protein levels were more apparent in Δe4–22^−/−^ mice. The impaired striatal LTD, increased neuronal excitability and impaired striatal-dependent learning in the Δe4–22^−/−^ mice have not been reported in other lines of *Shank3* mutants. Behavioural profiles among different lines of the mutant mice are also distinct. Although excessive grooming has been observed in other lines, skin lesions were reported only in Δe11, Δe13–16, and Δe4–22, but not in Δe4–7, Δe4–9, Δe9 and Δe21 (refs 20, 21, 22, 23, 24, 25, 26, 40), suggesting that the severity of over-grooming is *Shank3* isoform-specific.

The high levels of *Shank3* expression in striatum may explain the prominent striatal defects found in Δe4–22^−/−^ mice^21^. Changes in Homer1b/c and mGluR5 proteins were prominent in striatum, but not in neocortex and hippocampus. Furthermore, Δe4–22^−/−^ striatum had reduced spine density and attenuated PSD structures that are consistent with the deficiency of Shank3 and reduced Homer1 scaffolding proteins in this brain region. The decreased sEPSC frequency in Δe4–22^−/−^ MSNs, along with their reduced spine densities, suggests a loss of excitatory synapses. While the striatum receives glutamatergic inputs from both cortex and thalamus^41^, it is unclear whether both corticostriatal and thalamostriatal synapses are affected by the *Shank3* deletion. We interpret the significantly increased excitability of striatal Δe4–22^−/−^ MSNs as a homoeostatic compensation for reduced glutamatergic transmission, allowing spikes to be generated with less synaptic input. This increased excitability is consistent with our *in vivo* recordings, which show enhanced baseline functional connectivity between the cortex and striatum, yet reduced coupling between these areas during social interaction.

The Δe4–22^−/−^ mice also showed impaired striatal LTD, a form of synaptic plasticity associated with protein synthesis^42^. While this impairment might reflect reduced overall excitatory transmission, it may also be attributed to impaired mGluR5 signalling due to the disrupted mGluR5-Homer1 scaffolds, previously shown to be critical for striatal LTD^43^. This hypothesis is supported by the fact that impaired LTD in Δe4–22^−/−^ mice was completely rescued by CDPPB, a mGluR5 positive allosteric modulator. While CDPPB did not significantly alter the probability of PPR, it elevated levels of Homer1b/c in the Δe4–22^−/−^ PSD, implying that disrupted mGluR5-Homer scaffolds might be a major contributor to the impaired LTD. This deficit in LTD could be linked to the observed deficits in instrumental learning and other behavioural impairments, but not to the increased self-grooming in Δe4–22^−/−^ mice. Importantly, striatal-dependent instrumental learning was nearly abolished. Since instrumental learning involves acquiring new responses based on reward feedback^44^,^45^, our findings suggest a role for Shank3 in reward-related processes in general, including social rewards, potentially relevant to the pathophysiology of ASDs.

Abnormal brain connectivity has been proposed to underlie human autistic behaviours^3^,^4^,^6^, but the evidence is inconsistent due to both limitations in experimental design and heterogeneity of ASD presentation in humans. Mutant mice provide an experimentally tractable model to dissect the role of Shank3 in ASD-like behaviours. The overall brain volume and ventricular size were increased, consistent with the increased head circumference reported in a subset of *SHANK3* patients^19^. The abnormal neural circuit function and behaviours seen in our Δe4–22 mice are reminiscent of the alterations in grey matter content and the integrity of white matter reported in *SHANK3* patients. For example, enlargements in the basal ganglia found in Δe4–22^−/−^ mice correspond to reported enlargement in the neostriatum of human patients^46^. The Δe4–22^−/−^ mice have reduced cingulate cortices and enlarged deep mesencephalic nuclei, which receive inputs from the striatum and have projections to the ventral thalamus^47^. The changes in these structures may underlie the abnormal functional connectivity within the cortico-stiratial-thalamic circuit revealed from our *in vivo* recordings.

Multi-site *in vivo* recordings allowed us to document hyperactivity in the cortico-striatal-thalamic axis in Δe4–22^−/−^ mice. These recordings bear similarities to those in human ASD patients^9^,^10^. The altered pattern of oscillations in the 7–11 Hz frequency band in Δe4–22^−/−^ mice corresponds to the abnormalities at 8–11 Hz reported in human ASDs subjects^31^. As with our mice, hyperactivity in the anterior cingulate-striatal-thalamic circuits has been observed also in human subjects with ASDs^7^. A study of functional connectivity focusing on *SHANK3*-deficient patients may yield important new insights.

The deficiency of Shank3 in Δe4–22 mice caused a redistribution of Homer and mGluR5 in striatal neurons. The drastic changes for Homer1b/c (decrease in the PSD but increase in the soma) are consistent with a putative role of Shank3 in the synaptic trafficking of Homer1b/c, as previously suggested by *in vitro* studies^48^,^49^,^50^. The increased mGluR5 but disrupted mGluR5-Homer scaffolds in the PSD (presumably secondary to the mislocalized Homer1b/c) suggest a bidirectional alteration in mGluR5-mediated functions at synapses. The reduced association of Homer1 and mGluR5 could result in the hypofunction of mGluR5-mediated signalling, whereas the increased PSD-localized mGluR5 could be associated with enhanced mGluR5 function. This hypothesis of bidirectional change is supported by the elevation of basal p-ERK and p-S6K levels with a correspondingly blunted DHPG response. Because of differences in signalling between the striatum and hippocampus, it appears that mGluR5-mediated signalling could be synapse-and cell type-specific. Future experiments will investigate these possibilities. The bidirectional changes in mGluR5 function may contribute to the differential striatal-related behavioural impairments observed. That excessive grooming behaviour is moderated with the mGluR5 antagonist MPEP, but augmented with the positive allosteric modulator CDPPB, suggests that enhanced synaptic mGluR5 function underlies the excessive grooming seen in the Δe4–22^−/−^ mice. Conversely, partial rescue of lever-press by CDPPB raises the possibility that decreased mGluR5 function may contribute to the impaired instrumental learning.

Altered mGluR5 signalling has been documented in a mouse model of fragile X syndrome (FXS)^51^,^52^,^53^,^54^; recent work suggests that decreased association of mGluR5 with Homer1b/c contributes to the pathogenesis of this syndrome^55^,^56^. While our results are reminiscent of the disrupted mGluR5-Homer1 scaffolds in FXS mice, there are also important differences. First, the mGluR5-Homer scaffolds in Δe4–22^−/−^ mice are disrupted by reduced synaptic Homer1b/c, while there is no evidence that Homer1 protein is reduced in FXS mice^56^; moreover, Homer1a-mediated function is enhanced in FXS mice^55^. Second, altered striatal mGluR5 function in Δe4–22^−/−^ mice may be bidirectional, with no apparent evidence of altered protein synthesis, whereas in the FXS mouse both mGluR5 signalling and protein synthesis at hippocampal synapses are enhanced^52^,^53^. Third, the Δe4–22^−/−^mice show reduced striatal LTD, while FXS mice have enhanced hippocampal LTD^54^. These differences suggest that mGluR5 dysfunction may lead to distinct phenotypes in different brain regions and/or different genetic backgrounds. Phenotypic diversity has also been observed in other mouse models bearing mGluR5 dysfunction such as mouse models for TSC^57^,^58^ and human 16p11.2 deletion^59^. Nevertheless, the mGluR5 dysfunction shared in these models suggests that the mGluR5-Homer scaffold may represent a convergent molecular pathway underlying ASDs pathophysiology.

**Figure 9:**
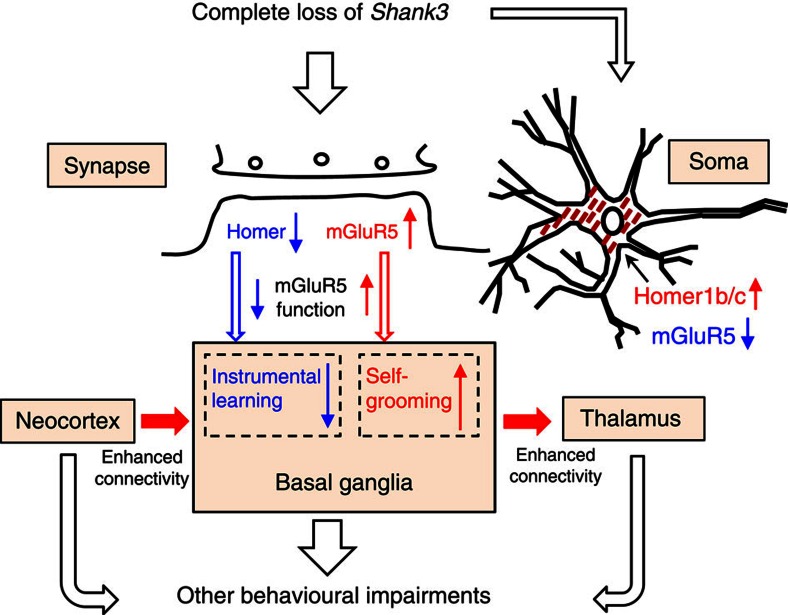
A proposed model of the impairments in *Shank3* completely deficient mice. In Δe4–22^−/−^ (−/−) mice, the deficiency in *Shank3* resulted in an abnormal redistribution of Homer1b/c and mGluR5 in synapses and soma of MSNs. Abnormal Homer1b/c trafficking in striatum leads to a reduction at synapses and accumulation in the soma. The decreased mGluR5-Homer association due to the reduced Homer1b/c at synapses perturbs mGluR5-mediated functions, and this contributes to the impaired instrumental learning and striatal synaptic plasticity. By comparison, the increased mGluR5 at the synapses may lead to enhanced mGluR5 signalling which contributes to the augmented self-grooming, and possibly the augmented functional connectivity in the cortical-striatal-thalamic axis. The behavioural phenotypes found in the *Shank3* completely deficient mice may reflect a complex interaction among changes at molecular, synaptic and neural circuit levels.

In summary, the present work suggests that many of the observed abnormalities in Δe4–22^−/−^ may be explained by disrupted mGluR5-Homer scaffolds and altered mGluR5 signalling in the striatum (Fig. 9). The deficiency of Shank3 protein leads to redistribution of Homer1b/c and mGluR5 protein at the synapse and soma. Our results suggest this could be a pathophysiological mechanism for ASDs in humans. While our findings indicate that these processes could be linked to distinct behavioural phenotypes and synaptic dysfunction, further studies will be required to establish exact mechanistic links among these molecular changes, behavioural impairments and neural circuitry dysfunction in Δe4–22^−/−^ mice.

## Methods

### Generation of *Shank3* mice with deletion of exons 4–22 (Δe4–22)

The targeting constructs were prepared using a previously described recombineering method^60^.The 129/SvEv BAC clone (bMQ457K21) covering the *Shank3* gene was first identified *in silico* using the Ensembl mouse genome browser (www.ensembl. org) and the clone was obtained from Geneservice Ltd, UK^61^. *Shank3*Δe4−22 mice were generated by a two-step targeting strategy using the Cre-*loxP* system. The first (5′) construct inserted the *loxP1* and *loxP2* sites flanking exons 4–9 with a neomycin cassette. The second (3′) construct inserted the *loxP3* at a 3′-site 5 kb-downstream of exon 22 with a puromycin cassette (Fig. 1a and Supplementary Fig. 1b). See details in Supplementary Methods. All experiments were conducted with protocols approved by the Institutional Animal Care and Use Committee at Duke University.

### Preparation of crude PSD and cytosolic fractions

Isolation of crude PSDs was performed using a previously described protocol^62^ with some modifications (see details in Supplementary Methods).

### Quantitative immunoblot and co-immunoprecipitation analysis

See details in Supplementary Methods. Images for immunoblot have been cropped for presentation. Full-size images are presented in Supplementary Fig. 9.

### Immunocytochemistry and confocal microscopy

Eight-week-old mice were transcardially perfused with 4% paraformaldehyde in 1 × PBS (pH 7.4). The brains were dissected and post-fixed in the same fixative overnight. The brains were then cryoprotected by submerging them in 30% sucrose for 48 h. After embedding into optimal cutting temperature compound (O.C.T.) on dry ice, brains were sectioned on a cryostat at 40 μm thickness. Slices with brain regions of interest were used for further staining with corresponding antibodies. See details in Supplementary Methods.

### Corticostriatal Co-cultured neurons and mGluR5 surface immunostaining

Corticostriatal co-cultures were prepared from P1 littermate Δe4–22^+/+^ and Δe4–22^−/−^ mice according to a method described previously^63^ (see details in Supplementary Methods).

### Antibodies

See details in Supplementary Methods

### Brain slices preparation and DHPG treatment

Hippocampal and striatal slices were prepared from 4-week-old mice, anaesthetized with isoflurane. Brains were dissected rapidly and cut coronally at 400 μm in dissection buffer containing the following (in mM): 75 sucrose; 87 NaCl; 2.5 KCl; 1.25 NaH_2_PO_4_; 26 NaHCO_3_; 10 glucose; 7 MgCl_2_; and 0.5 CaCl_2_. Slices were allowed to recover at 32 °C for 1 h in a submersion chamber containing oxygenated artificial cerebrospinal fluid (aCSF) consisting of the following (in mM): 124 NaCl; 3 KCl; 1.25 NaH_2_PO_4_; 26 NaHCO_3_; 20 glucose; 1 MgCl_2_; and 2 CaCl_2_. For DHPG treatment, slices were transferred into six-well plates with or without 100 μM DHPG for 5 min. After treatment, slices were transferred into a 1.5 ml tube and frozen on dry ice for further western blot analysis. Protein synthesis in the striatal slices was evaluated by measuring puromycin incorporation into newly synthesized proteins using a puromycin antibody as described^52^.

### Golgi staining

Golgi staining was performed using 8-week-old mice as described^22^. See details in Supplementary Methods.

### Electron microscopy

Electron microscopy was performed using 8-week-old mice as described^22^. See details in Supplementary Methods.

### Mouse cohorts for the behavioural tests

Mice were tested in 10 cohorts of mixed sex Δe4–22^+/+^, Δe4–22^+/−^ and Δe4–22^−/−^ littermates beginning at 2 mos of age (except for neonatal studies as described below) (Supplementary Table 1). Many of the methods described below (some behavioural anaylses are described in Supplementary Methods) have been reported previously by our group, allowing us to determine appropriate numbers of mice per cohort to assess significant differences^22^,^64^. Since some mice developed lesions across testing, they were euthanized according to Duke IACUC policy and thus were excluded from subsequent tests. All experimenters handling animals or scoring behavioural data were blinded to genotype throughout the studies. Mice in cohorts 1–6, 9,10 were housed on a 14:10 h light:dark cycle with most testing occurring during the light cycle. Mice in cohorts 7 and 8 were housed on a 12:12 light:dark cycle. Mice were housed 4–5 per cage unless otherwise specified.

### Spray test

Individual animals were acclimated to clean home cages for 5 min before filming (MediaRecorder2, Noldus Information Technologies, Leesburg, VA, USA). Mice were filmed for 5 min and then lightly misted with tap water from a spray bottle. Filming continued for an additional 10 min. Grooming behaviour was scored using TopScan software (CleverSys, Reston, VA, USA) and verified by a blinded observer. Because of the differing length of pre- and post-spray responses, the data were reported as seconds spent in self-grooming per min observation.

### Hole-board test

Mice were placed individually into a 42 × 42 × 30 cm open field and allowed free exploration of a hole-board apparatus (42 × 42 × 3 cm) (AccuScan Instruments, Columbus, OH, USA) as described^22^. The hole-board was made of white plexiglass with 16 equally spaced holes (3 cm in diameter) arranged in four rows. Animals were filmed with a digital video camera and hand-scored by a blinded observer for the numbers of nose-pokes and the location of each nose-poke.

### Juvenile nest choice

This test was conducted on postnatal day 15, when pups had opened their eyes and were capable of thermoregulation and goal-directed locomotor behaviour^65^. Testing occurred in two phases. For the first phase, 2 cm^2^ nesting material was removed from the home-cage nest and placed at one end of a clean and empty mouse cage. Testing began when a single pup was placed at the end of the cage opposite to the nesting material, with the head of the pup facing away from the nest. The latency of the pup to turn and enter the home nest was scored. If the pup did not find the home nest after 60 s, the latency was scored as 60 s. At the end of the test, pups were quickly removed and placed into a clean 8 oz. holding cup. The testing cage was cleaned of any urine or boli, and an equal amount of stranger nesting materials was placed in the test cage opposite that of the home-cage material. For the second test, the pup was returned to the testing cage and placed between the two nesting samples and facing the side of the cage. Behaviour was scored for 60 s and the latency to make the first choice (familiar home-cage nest or stranger nest) was recorded. All tests were video-recorded and later scored using the Noldus Observer software (Noldus).

### Social dyadic test

Male mice were housed individually for >14 days before testing^22^,^66^. All testing was performed under red-light illumination (<5 lux) 2–6 h after onset of the dark cycle. Plexiglass test chambers (48 × 26 × 20 cm) were cleaned between each test with LabSan 256CPQ solution (Sanitation Strategies LLC, Williamston, MI) and refilled with 1/8” cob bedding (Andersen Inc., Maumee, OH, USA). *Shank3* males were paired to non-familiar C3H/HeJ males (Jackson Labs, Stock No. 000659) of the same age and approximate weight. Each *Shank3* mouse and its C3H partner were placed at opposite ends of test chamber and were separated by a solid partition. After 5 min, the barrier was removed and the animals were allowed to interact freely. All tests were filmed and the videos were scored by observers blinded to the genotype of the animals using the Observer XT 9 software (Noldus Information Technologies, Leesburg, VA). The ethogram for behavioural scoring consisted of 24 behaviours^67^ and were collapsed into two categories of behaviour: the total time spent in bidirectional social interaction, consisting of one mouse engaging the other and the other animal reciprocating the social behaviour; and total time spent in non-reciprocated interaction, wherein the *Shank3* mouse's responses were not reciprocated by the C3H partner. All animals were scored also for self-grooming. Only one animal engaged in fighting necessitating early termination of the experiment; this animal was excluded from analysis.

### Pup ultrasonic vocalizations

The USVs of neonates were examined during brief maternal separation on postnatal day 4. USVs from individually-isolated pups were recorded using an externally polarized condenser microphone with a frequency range of 10–200 kHz that was attached 15–20 cm above the floor of an isolation chamber. The microphone was connected to the Avisoft-UltrasoundGate recording software (Avisoft Bioacoustics, Berlin, Germany) and the pup-emitted calls were recorded to WAV sound files using parameters optimized for mice^22^. Pups were individually placed in the sound-proof chambers and calls were recorded for 60 s. WAV files for each pup were converted to spectrograms and analysed with automated whistle tracking parameters by the Avisoft SASLab Pro software (Avisoft Bioacoustics).

### Adult ultrasonic vocalizations

The Δe4–22 males were given 10–14 days of reproductive experience with a female partner before recordings vocalizations. USVs were examined by exposing adult male Δe4–22 mice to a novel, 8–12 week-old C57BL/6J female (Jackson Labs). Recordings were performed as described for pups except the microphone was suspended 25 cm above the centre of the test chamber floor. Test mice were acclimated to the chambers for 10–15 min. Next, a female was introduced and USVs were recorded for 5 min. Ultrasonic calls were recorded as described for pups. To avoid possible white-noise artifacts, frequencies outside the 25–120 kHz range were truncated and not included in the analyses. To avoid other ultrasonic artifacts, sounds <2 ms in duration were also excluded from the analyses^68^. Mice which did not call were excluded from subsequent analyses of call duration, peak amplitude and peak frequency.

### Open-field activity

Spontaneous activity in the open field was conducted over 1 h in an automated Omnitech Digiscan apparatus (AccuScan Instruments, Columbus, OH, USA). Accuscan software scored the total distance travelled, vertical activity (beam-breaks), and time spent in the centre zone.

### Light–dark emergence test

Mice were placed into the darkened side (∼2 lux) of a two-chambered apparatus (Med-Associates, St Albans, VT) and given 5 min to freely explore the darkened and lighted (∼750 lux) chambers. Infrared diodes within the test chamber tracked the location and activity of the animal throughout testing. The scored behaviours comprised the latency to enter into the lighted chamber, and the numbers of crossings between the lighted and darkened chambers.

### Reactivity in a novel environment

A cohort of Δ4–22^−/−^ mice engaged in frequent escape behaviours from the chambers during testing for novel object recognition memory. To determine whether they had enhanced reactivity to a novel environment, testing was conducted in three phases over 10 min each: observation in the home cage, observation in a new cage, and observation in the novel object recognition memory chamber (41 × 18 × 30 cm, opaque plexiglass). A blinded observer recorded escape behaviours.

### Rotarod performance

Rotarod (Med-Associates) performance was tested as described^69^. On day 1 the rod accelerated from 4 to 40 r.p.m. over 5 min, and mice were given four successive 5 min trials with an inter-trial interval of 30 min. Trials were terminated when the mouse fell from the rod or at 300 s. On day 2 the rod was maintained at a steady speed of 16 r.p.m., and four trials were conducted in the same manner as on day 1.

### Continuous reinforcement training

Mice were placed on a food deprivation schedule to reduce their body weights to ∼85% of normal. Water was available at all times in the home cages. Training took place in Med-Associates operant chambers housed within a light-resistant and sound-attenuating chamber. The chamber was equipped with a food magazine that delivered 14 mg dustless precision food pellets (Bio-Serv, Flemington, NJ, USA) and with a retractable lever located on the left side of the magazine. A computer with the Med-PC-IV program was used to control the equipment and record behaviour. Lever-press training consisted of 7 days of continuous reinforcement (each press earned one food pellet). Each session began with illumination of the house light and insertion of the lever into the chamber, and terminated with extinguishing the house light and retraction of the lever after 60 min or after 100 earned pellets (whichever came first). For CDPPB treatment, the pellet dispenser was replaced with a liquid dipper which delivered a drop of sweetened, condensed milk for each lever press.

### Drug treatments

The mGluR5 antagonist MPEP (M5345) and the mGluR5 positive allosteric modulator CDPPB (SML0235) were purchased from Sigma-Aldrich (St Louis, MO, USA). MPEP was dissolved in 0.9% normal saline; CDPPB was dissolved in 0.5% methylcellulose. MPEP (20 mg kg^−1^) or CDPPB (10 mg kg^−1^) were administered by intraperitoneal injection 30 min before behavioural testing.

### Magnetic resonance histology

Brains of Δe4–22^−/−^ mice (*N*=7) and control C57BL/6J (*N*=7, aged 60–74 days) were actively stained, using a transcardiac perfusion protocol; after flushing out the blood with PBS, the tissue was fixed using a 10% formalin solution with 5 mM gadoteridol (ProHance, Bracco Diagnostics Inc.). Specimens were stored in formalin overnight then rehydrated in a PBS+ProHance (1:200) solution until imaging. Brain specimens were imaged within the cranium to avoid distortions and damage, using high field (9.4T) MRH, with a DTI protocol consisting of 1 baseline (*b*=0) and diffusion in six directions (non-collinear diffusion gradient vector directions [1, 1, 0], [1, 0, 1], [0, 1, 1], [−1, 1, 0], [1, 0, −1] and [0, −1, 1]), using *b* values of 1,495 s mm^−2^, with TE-11.82 ms, TR=100 ms, 512 × 256 × 256 matrix, 22 × 11 × 11 mm field of view, diffusion pulse amplitude 1,000 ms m^−1^, separation 5.76 ms, width 1.3 ms, at isotropic resolution of 43 μm. Images were processed to construct tensors and DTI parametric maps using the Diffusion Toolkit (http://trackvis.org/dtk/). Image segmentation and analysis were described in Supplementary Methods.

### Whole-cell patch-clamp recording from brain slices

Acute striatal brain slices were prepared from adult mice (2–5 mos) using a Vibratome (see detail in Supplementary Methods). Recordings were made from MSNs in the DLS, identified visually with the aid of differential interference contrast-enhanced visual guidance. For voltage-clamp recordings, cells were clamped at −70 mV throughout the experiments. Synaptic currents were recorded with an Axopatch 1D amplifier (Axon Instruments), filtered at 5 kHz, and digitized at 10 kHz. EPSC amplitudes were examined using peak detection software in pCLAMP10 (Molecular devices). Slices were maintained at a temperature between 28 and 30 °C and were stable within ±1 °C during the experiment.

For evoked EPSCs, test stimuli were delivered via a Master-8 stimulator (A.M.P.I., Jerusalem, Israel) at a frequency of 0.05 Hz through a bipolar twisted tungsten wire placed into the striatum. The duration and intensity of the stimulation was adjusted so that the size of the evoked EPSCs was 200–500 pA. We analysed only recordings with series resistance <20 MΩ. The series resistance was not compensated, and if it changed more than 20% during the recording, the cell was not included in the analysis.

For current injection experiments, the recordings were performed in current clamp mode. Each step was 100 pA with a duration of 1,000 ms, and the number of spikes for each depolarizing step was counted. EPSC amplitudes were examined using peak detection software in pCLAMP10 (Molecular Devices, Sunnyvale, CA, USA). The HFS protocol used to induce LTD was paired with depolarization of the postsynaptic cell to 0 mV and it consisted of four 1 s, 100 Hz trains delivered every 10 s (ref. 42). For the effects of CDPPB on sEPSCs and LTD, 10 μm (final concentration) of CDPPB was added in aCSF external solution for whole-cell patch clamp recording.

### Multi-circuit *in vivo* recording

Three- to four- mos old Δe4–22^−/−^ mice and their Δe4–22^+/+^ littermate controls were anaesthetized with ketamine (100 mg kg^−1^) and xylazine (10 mg kg^−1^), placed in a stereotaxic device, and metal ground screws were secured to the skull above the cerebellum and at the anterior cranium. A total of 32 tungsten microwires were arranged in bundle arrays of 4–12 wires (each wire separated by at least 250 μm), and implanted as follows (listed as AP, ML, DV; all coordinates are measured in mm from bregma, except DV which is from dura).: V_HIP: −3.4, 3, 3.25; THAL: −1.5, −0.35, 2.88; NAC: 1.25, 1.15, 3.9; PRL_CX: 1.7, −0.25, 1.25; CG_CX: 1.98, −0.25, 0.75.

Neurophysiological experiments were initiated following a 3-week recovery and were conducted during the light cycle. Full details of the procedures for electrode construction and surgical implantation have been previously described^70^. Neuronal activity was sampled at 30 kHz, high-pass filtered at 250 Hz, sorted online, and stored using the Cerebus acquisition system (Blackrock Microsystems Inc., Salt Lake City, UT, USA). Neuronal data were referenced online against a wire within the same brain area that did not exhibit a signal-to-noise ratio greater than 3:1. At the end of the recording, cells were sorted again using an offline sorting algorithm (Plexon Inc., TX) to confirm the quality of the isolated cells. LFPs were low-pass filtered at 250 Hz and stored at 1,000 Hz. A second 0.5 Hz high-pass filter was applied before neurophysiological analysis. All neurophysiological recordings were referenced to a ground wire connected to both ground screws. Wires tested from the two screws were iso-electric, demonstrating that ground loops were not introduced by this design.

### LFP oscillatory power and cross-area coherence

See details in Supplementary Methods.

### LFP phase analysis

See details in Supplementary Methods.

### Forced social interaction test

Test mice were habituated under a wire cage (Galaxy Utility Cup, www.kitchen-plus.com) for 30 min in their home-cage. The wire cage was then transferred to a plexiglass chamber (dimensions: 40 cm × 23 cm × 23 cm). Following an additional 5 min habituation period, neurophysiological activity was recorded for 10 min. An unfamiliar C3H/HeJ mouse (Jackson Labs) was introduced into the arena 5 min after the beginning of the 10 min neurophysiological recording.

### General statistical analyses

The data were analysed with SPSS 21 (SPSS Inc., Chicago, IL, USA) or Microsoft Excel and expressed as means±s.e.m. Simple comparisons between *Shank3*Δe4–22^+/+^ and *Shank3*Δe4–22^−/−^ mice without regards to sex were conducted with independent *t*-tests. In cases where comparisons among the 3 genotypes were made or were related to test conditions, analysis of variance (ANOVA) tests were used. When comparisons between genotypes were made for within-subject measurements across different phases of the same test (for example, test days, locations within a test arena, or different intensities of stimuli), the data were analysed with repeated measures ANOVA (RMANOVA). When comparisons between genotypes of different groups (that is, sex, age or treatment condition) were made, two- or three-way ANOVA were used. For one-way ANOVAs, Fisher's LSD method was used for the *post hoc* comparisons; for RMANOVAs and multi-factor ANOVAs, a Bonferroni-correction for multiple comparisons was applied. Statistical significance was defined as *P*<0.05. Statistical analyses of *in vivo* data is described in Supplementary Methods. Detailed statistics for all the results can be seen in Supplementary Data 1.

## Additional information

**How to cite this article:** Wang, X. *et al*. Altered mGluR5-Homer scaffolds and corticostriatal connectivity in a *Shank3* complete knockout model of autism. *Nat. Commun.* 7:11459 doi: 10.1038/ncomms11459 (2016).

## Supplementary Material

Supplementary Information Supplementary Figures 1-9, Supplementary Tables 1-6, Supplementary Methods and Supplementary References.

Supplementary Data 1 Statistics for all results.

Supplementary Movie 1 Grooming of Shank3 mutant mice.
