# Supplementary material for: Altered mGluR5-Homer scaffolds and corticostriatal connectivity in a Shank3 complete knockout model of autism
Source: Nat Commun. 2016 May 10;7:11459. doi: 10.1038/ncomms11459 (PMC4866051; doi:10.1038/ncomms11459)
Supplement: Supplementary Information — Supplementary Figures 1-9, Supplementary Tables 1-6, Supplementary Methods and Supplementary References. [file ncomms11459-s1.pdf]

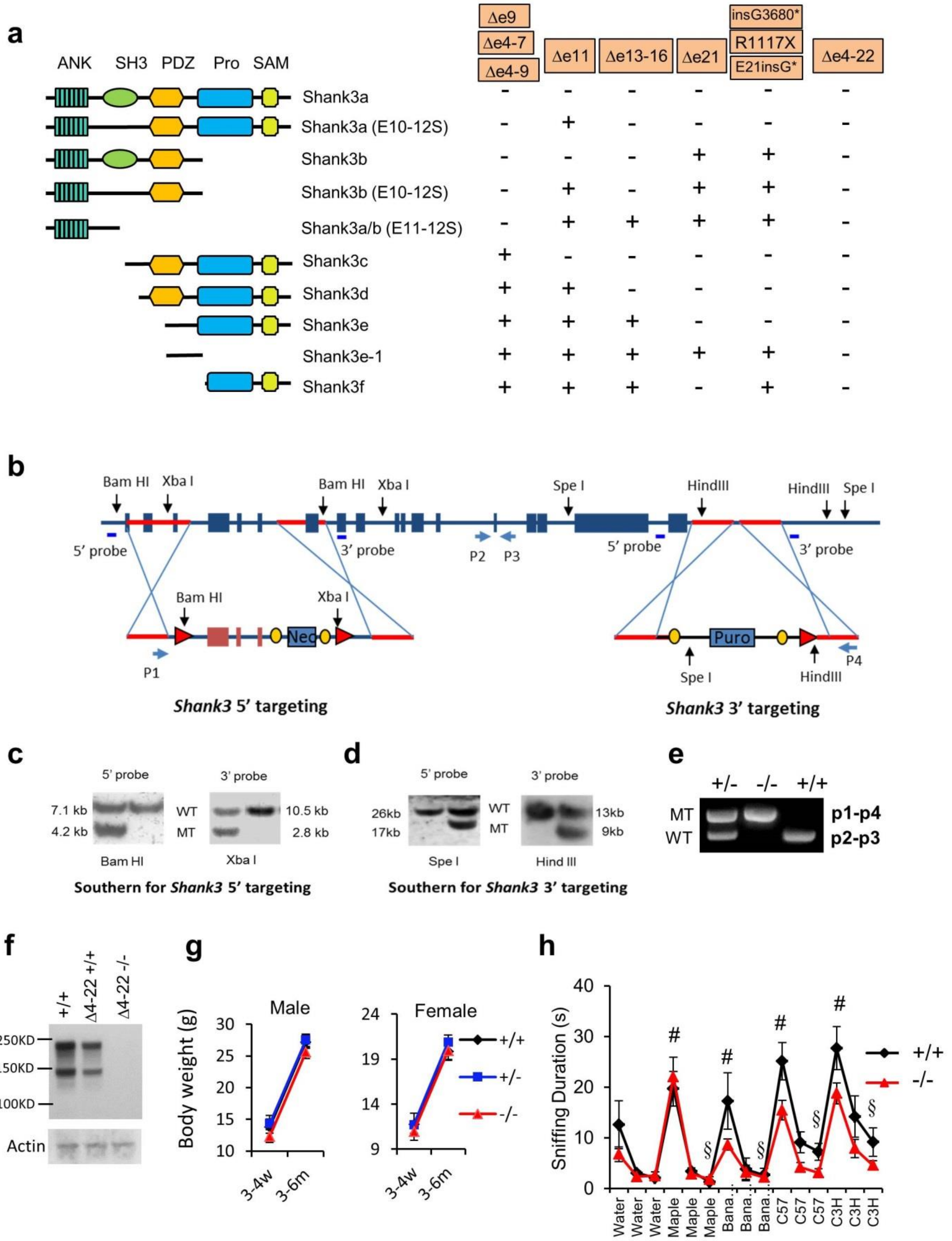
a
ANK SH3 PDZ Pro SAM
Δe9
Δe4-7
Δe4-9
Δe11
Δe13-16
Δe21
insG3680*
R1117X
E21insG*
Δe4-22
Shank3a - - - - - -
Shank3a (E10-12S) - + - - - -
Shank3b - - - + + -
Shank3b (E10-12S) - + - + + -
Shank3a/b (E11-12S) - + + + + -
Shank3c + - - - - -
Shank3d + + - - - -
Shank3e + + + - - -
Shank3e-1 + + + + + -
Shank3f + + + - + -
b
Bam HI
Xba I
Spe I
HindIII
5' probe
3' probe
P1
P2
P3
P4
Neo
Puro
Shank3 5' targeting
Shank3 3' targeting
c
5' probe
3' probe
7.1 kb
4.2 kb
WT
MT
10.5 kb
2.8 kb
Bam HI
Xba I
Southern for Shank3 5' targeting
d
5' probe
3' probe
26kb
17kb
WT
MT
13kb
9kb
Spe I
Hind III
Southern for Shank3 3' targeting
e
+/- -/- +/+
MT
WT
p1-p4
p2-p3
f
+/+
Δ4-22 +/+
Δ4-22 -/-
250KD
150KD
100KD
Actin
g
Male
Female
Body weight (g)
3-4w
3-6m
+/+
+/-
-/-
h
Sniffing Duration (s)
Water
Maple
Bana.
C57
C3H
#
§
+/+
-/-

Supplementary Figure 1. Generation of *Shank3* Δe4-22 mutant mice. (a) Pattern of *Shank3* isoforms disrupted in different lines of *Shank3* mutant mice[1, 2, 3, 4, 5, 6, 7, 8, 9](*mutation of exon 21 insG[9] and insG3680[8] are same mutation). The "-" indicates that the isoform is disrupted and the "+" indicates the isoform remains intact. **(b)** Generation of the *Shank3* completely deficient mice by a two-step targeting strategy using Cre/*loxP* system. The first (5') construct had *loxP1* and *loxP2* sites flanking exons 4-9. The second (3') construct had a *loxP3* site inserted 3' downstream (5 kb) of exon 22. These constructs were sequentially introduced into 129R1-derived ES cells and germ-line transmission for the floxed alleles was obtained by injection into C57BL/6J embryos. The positions of the genomic Southern blot probes and PCR primers for genotyping are indicated. **(c)** DNA genomic Southern blot confirmation of the insertion of the *loxP1-2* sites using the 5' and 3' probes, respectively. WT, wild-type allele; MT, mutant allele. (**d**) Genomic Southern blot confirmation of the insertion of the *loxP3* site using the 5' and 3' probes, respectively. (**e**) PCR genotyping of Δe4-22 mice using PCR. The P1-P4 primer pair produced a 600 bp band identifying the Δe4-22 allele, while the P2-P3 primer pair amplified the 200 bp product from the wild-type allele. **(f)** Western blot revealed an absence of all Shank3 protein isoforms in -/- whole brain lysate using the C-terminal Shank3 antibody (Santa Cruz, sc-30193). Experiments for western blots were repeated at least three times. **(g)** Body weights of Δe4-22 mice at weaning and 3-6 months of age. Both male and female Δe4-22$^{-/-}$ (-/-) mice were not significantly different from those of Δe4-22$^{+/+}$ (+/+), Δe4-22$^{+/-}$ (+/-) littermates at weaning or 3-6 months of age. n=9-15 mice/genotype & sex. (**h**) Normal olfactory habituation-dishabituation. While all mice sampled the stimuli, -/- mice sampled less than +/+ littermates [RMANOVA, effect of genotype $F_{(1,15)} = 5.16$, $p=0.038$] and both genotypes showed significant habituation-dishabituation [effect of trial $F_{(14,210)} = 24.27$, $p<0.001$; trial x genotype not significant.] $^{\S}p<0.05$, within genotype for habituation (1st vs. 3rd trial of same odor); $^{\#}p<0.05$, within genotype for dishabituation (3rd vs. 1st trial of new odor). All data are expressed as means ± SEM.

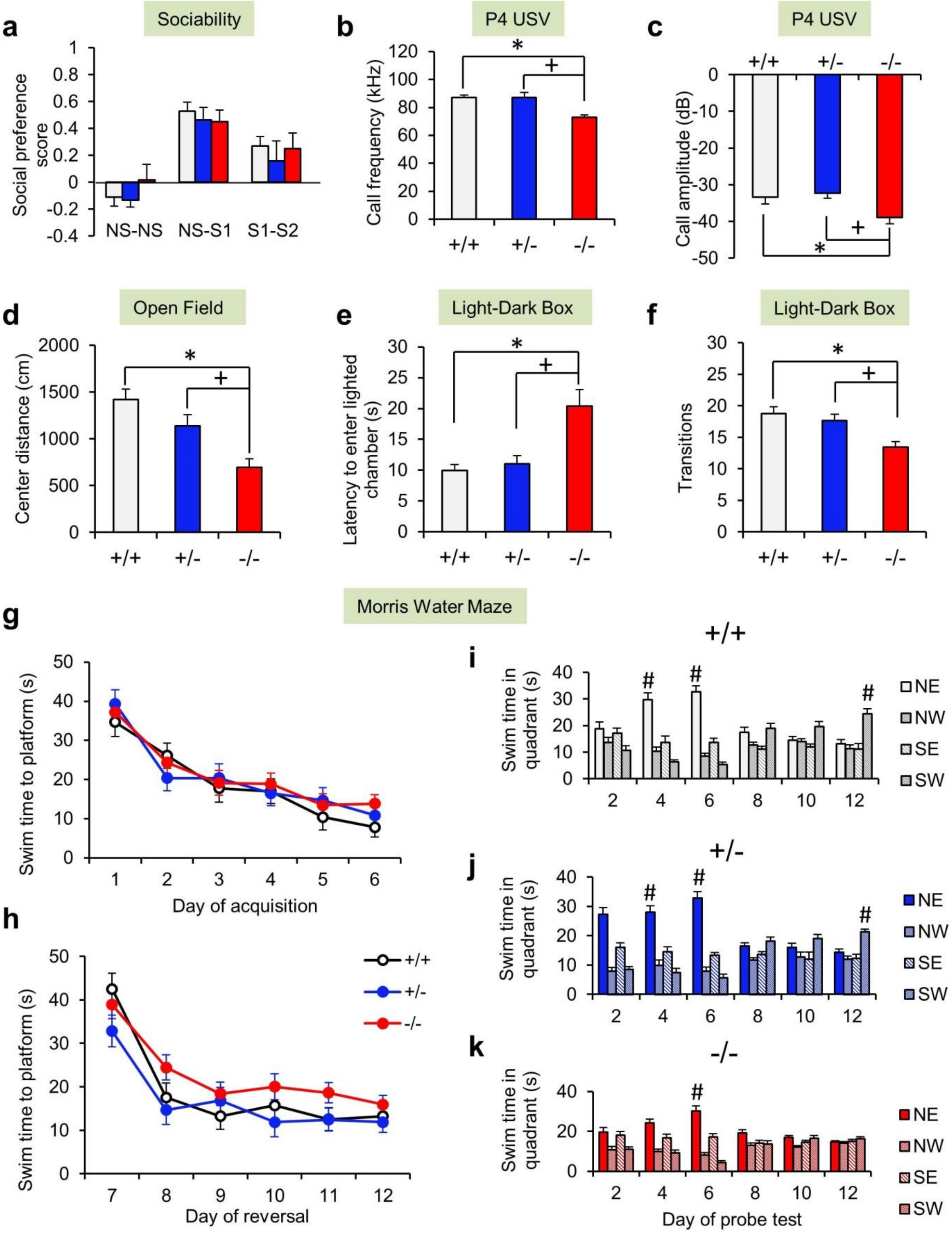

a
Sociability
Social preference score
NS-NS
NS-S1
S1-S2
b
P4 USV
Call frequency (kHz)
+/+
+/-
-/-
*
+
c
P4 USV
Call amplitude (dB)
+/+
+/-
-/-
*
+
d
Open Field
Center distance (cm)
+/+
+/-
-/-
*
+
e
Light-Dark Box
Latency to enter lighted chamber (s)
+/+
+/-
-/-
*
+
f
Light-Dark Box
Transitions
+/+
+/-
-/-
*
+
Morris Water Maze
g
Swim time to platform (s)
Day of acquisition
h
Swim time to platform (s)
Day of reversal
+/+
+/-
-/-
i
+/+
Swim time in quadrant (s)
#
NE
NW
SE
SW
j
+/-
Swim time in quadrant (s)
#
NE
NW
SE
SW
k
-/-
Swim time in quadrant (s)
#
NE
NW
SE
SW
Day of probe test

Supplementary Figure 2. ***Shank3*** **Δe4-22 mice display ASD-related and comorbid behaviors.** **(a)** Normal social affiliation and social preference in *Shank3* Δe4-22 mice. No genotype differences were evident for preferences between the identical non-social (NS-NS) objects [Δe4-$22^{+/+}$ (+/+), Δe4-$22^{+/-}$ (+/-), and Δe4-$22^{-/-}$ (-/-)]. In the NS-S1 pairing, all three genotypes demonstrated a preference for the novel social partner. When presented with familiar and novel social stimuli (S1-S2), all mice preferred the novel social partner; n=9 mice/genotype. (**b-c)** Altered pup USV spectral properties**.** On postnatal day 4 the frequencies (kHz) of USVs emitted during isolation were lower (b) [ANOVA: $F_{(2,29)}=6.63$, $p<0.005$] and the amplitude (dB) of calls was attenuated in -/- pups (c) [ANOVA: $F_{(2,29)}=4.41$, $p<0.03$] relative to the other genotypes (*ps*<0.05); n=8-13 mice/genotype. **(d-f)** Anxiety-like behaviors across different tests. (d) -/- mice travelled less distance in the center of the open field than the other genotypes (*p*s<0.01) [ANOVA: $F_{(2,23)}=9.67$, $p<0.001$]; n=6-10 mice/genotype. In the light-dark box, -/- mice had prolonged latencies to emerge from the darkened into the lighted chamber (e) [ANOVA: $F_{(2,60)}=9.68$, $p<0.001$] and they made fewer transitions between chambers (f) [ANOVA: $F_{(2,60)}=9.15$, $p<0.001$] than the other genotypes (*p*s<0.02); n=20-21 mice/genotype. **(g-k)** Normal spatial learning with delayed reversal learning for -/- mice. Swim times were similar for all 3 genotypes during acquisition in the Morris water maze (g). When the hidden platform was moved to a new location (h), swim times were increased for -/- mice [RMANOVA, effect of genotype $F_{(2,30)}=3.46$, $p<0.05$], but individual daily comparisons relative to the other genotypes were not significant; n=10-13 mice/genotype. **(i-k)** Probe tests were conducted during acquisition (days 2, 4 and 6) and reversal (days 8, 10 and 12), measuring swim times in each quadrant for +/+ (i), +/- (j), and -/- mice (k). During acquisition, all mice preferred swimming in the NE quadrant. At reversal, only -/- mice (*p*s<0.05) failed to develop a significant preference for the SW quadrant [RMANOVA within subject contrasts (quadratic, quadratic): day x zone x genotype interaction: $F_{(12,180)}=5.79$, $p<0.01$]; n=10-13 mice/genotype. For all tests: $*p<0.05$, -/- versus +/+; $^{+}p<0.05$, -/- versus +/-; $^{\#}p<0.05$, within genotype for target quadrant compared to other quadrants. All data are expressed as means ± SEM.

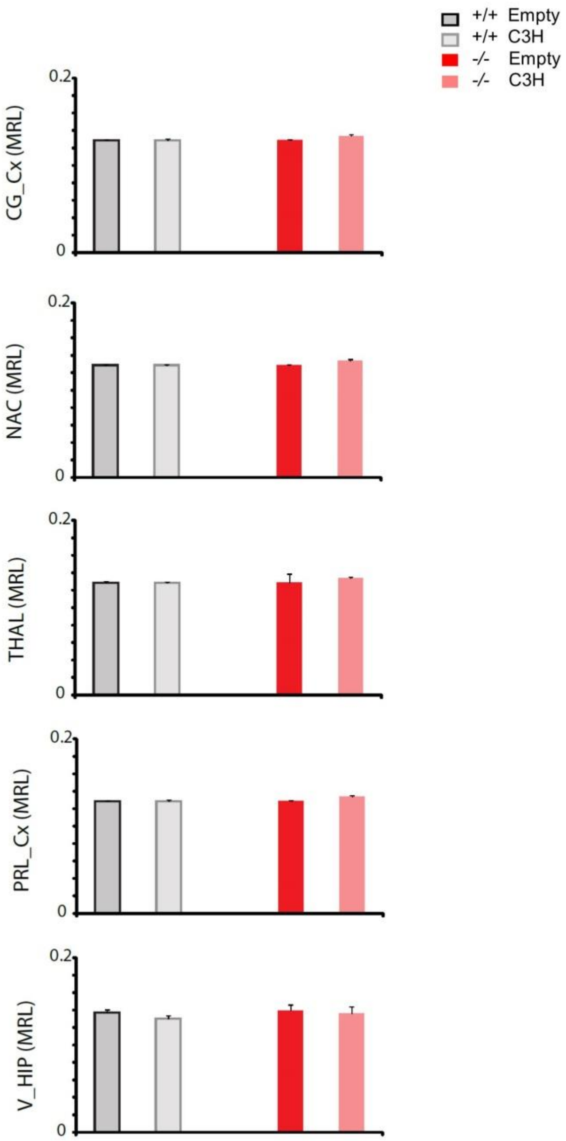

Supplementary Figure 3. Local connectivity is not altered in Δe4-22$^{-/-}$ mice. Single units were recorded and mean resultant length (MRL) were calculated from CG_CX (n = 89 and 62), NAC (n = 105 and 149), THAL (n = 21 and 25), PRL_CX (n = 51 and 26), and V_HIP (n = 15 and 9) in Δe4-22$^{+/+}$ (+/+) and Δe4-22$^{-/-}$ (-/-) mice, respectively. The phase locking of each single unit to locally recorded 7-11Hz oscillations was calculated for both genotypes using a mixed model ANOVA of genotype X test condition with Box-Cox transform and Bonferroni-correction for multiple comparisons. No significant genotype or genotype x test condition effects were identified. All data are expressed as means ± SEM.

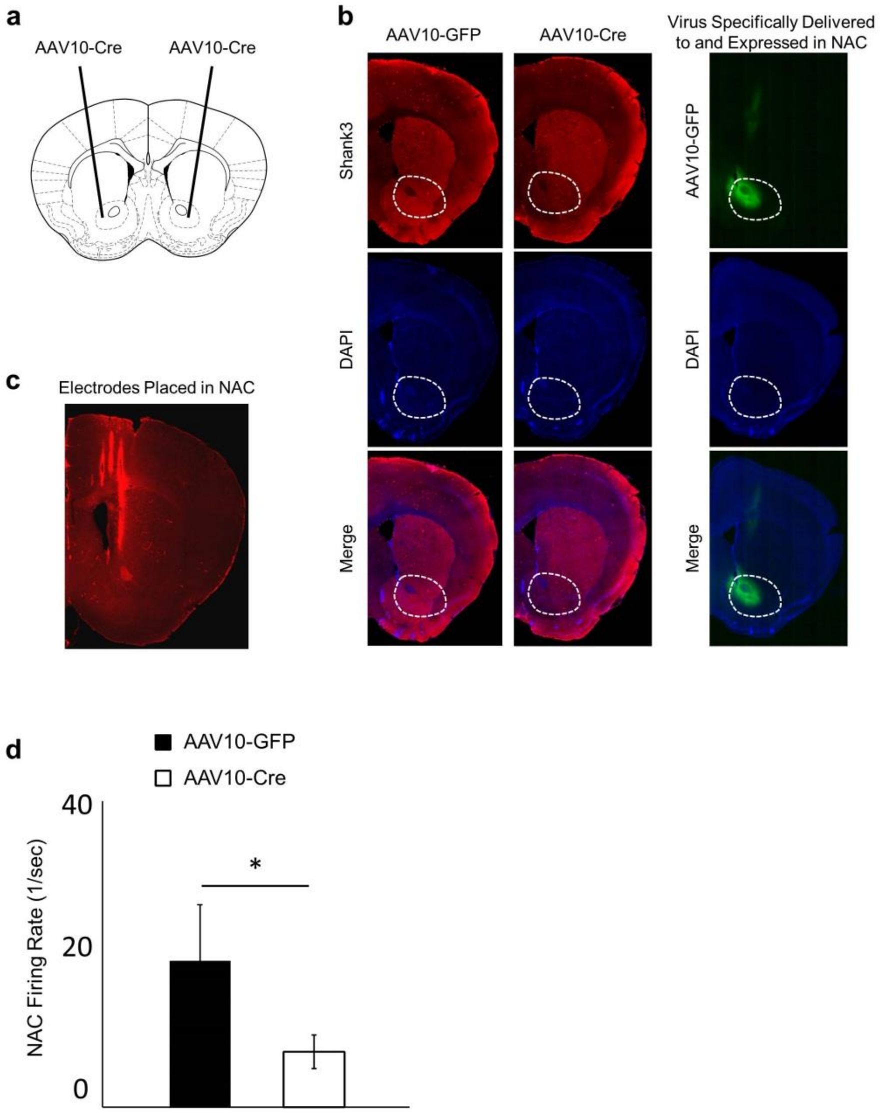

Supplementary Figure 4. NAC selective *Shank3* knockdown. (a) Schematic for NAC selective *Shank3* knockdown. **(b)** Images confirming Shank3 knockdown following infection with AAV10-Cre virus (left panels), and images showing specific viral expression in NAC (right panel). **(c)** Images showing correct placement of recording electrodes implanted in NAC. **(d)** Lower NAC firing rates were observed in the *Shank3* knockdown mice compared to controls. $*p = 0.03$ using Wilcoxon rank-sum test; N = 19 and 6 neurons recorded from 8 mice treated with AAV10-Cre, and 3 mice treated with AAV10-GFP, respectively). All data are expressed as means ± SEM.

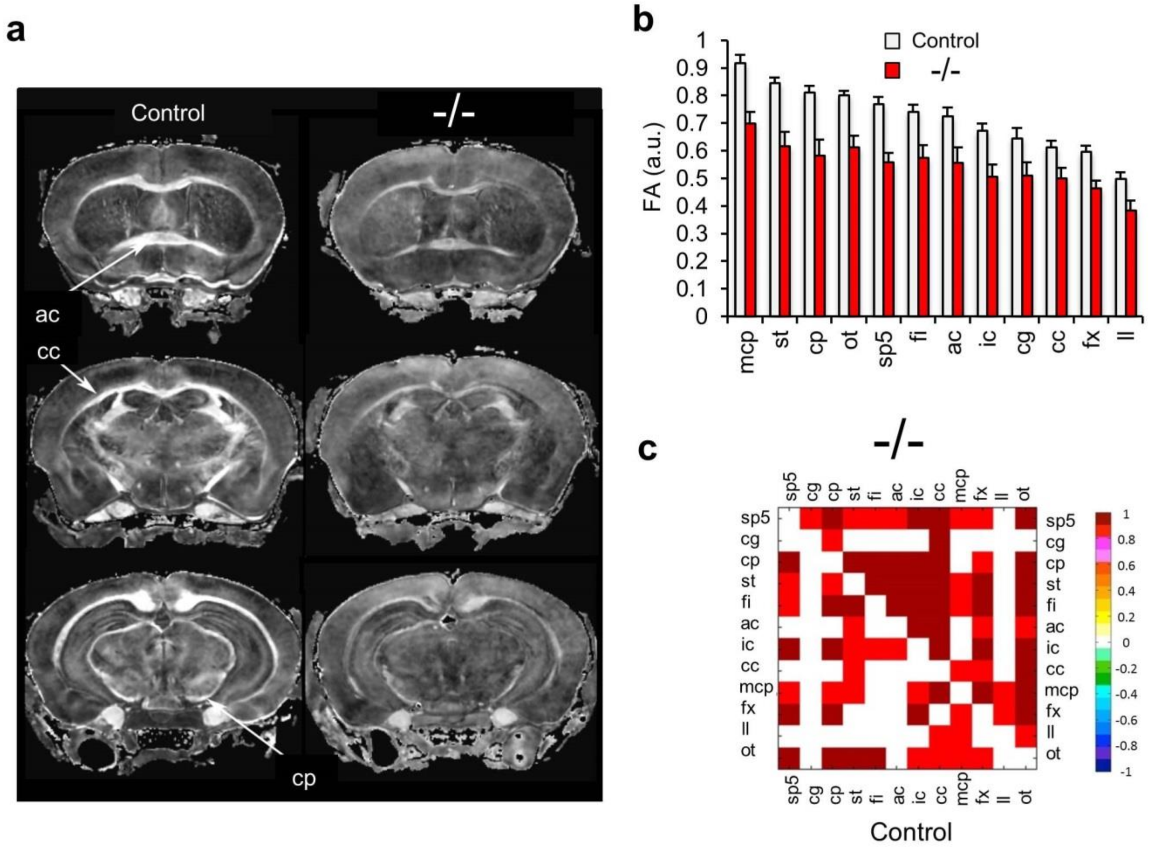

Supplementary Figure 5. Brain structural changes in Δe4-22$^{-/-}$ mice. (a) A qualitative evaluation of Δe4-22$^{-/-}$ (-/-) mice shows reduced contrast between white and gray matter areas. The coronal images at different levels through the brain suggest lower FA values for white matter tracts throughout the -/- brains. Shown are the anterior commissure (ac), corpus callosum (cc), and cerebral peduncles (cp). **(b)** Significant FA differences (averaging 23% reduction) were found between -/- mice and controls for white matter tracts including the middle cerebellar peduncle (mcp) (p=0.001), stria terminalis (st) (p=0.002), cerebral peduncle (cp) (p=0.004), optic tract (ot) (p=0.002), spinal trigeminal (sp5) (p=0.001), fimbria (fi) (p=0.009), anterior commissure (ac) (p=0.03), internal capsule (ic) (p=0.009), cingulum (cg) (p=0.046), corpus callosum (cc) (p=0.03), fornix (fx) (p=0.004), and lateral lemniscus (ll) (p=0.023) , two-tailed t-test, n=6 mice/genotype. **(c)** Correlations between changes in FA relative to the cortex were higher in -/- mice than controls. Major fiber tracts including the anterior commissure (ac), internal capsule (ic), and corpus callosum (cc) which co-varied significantly. Pearson's linear correlation, two tailed, p<0.05. All data are expressed as means ± SEM.

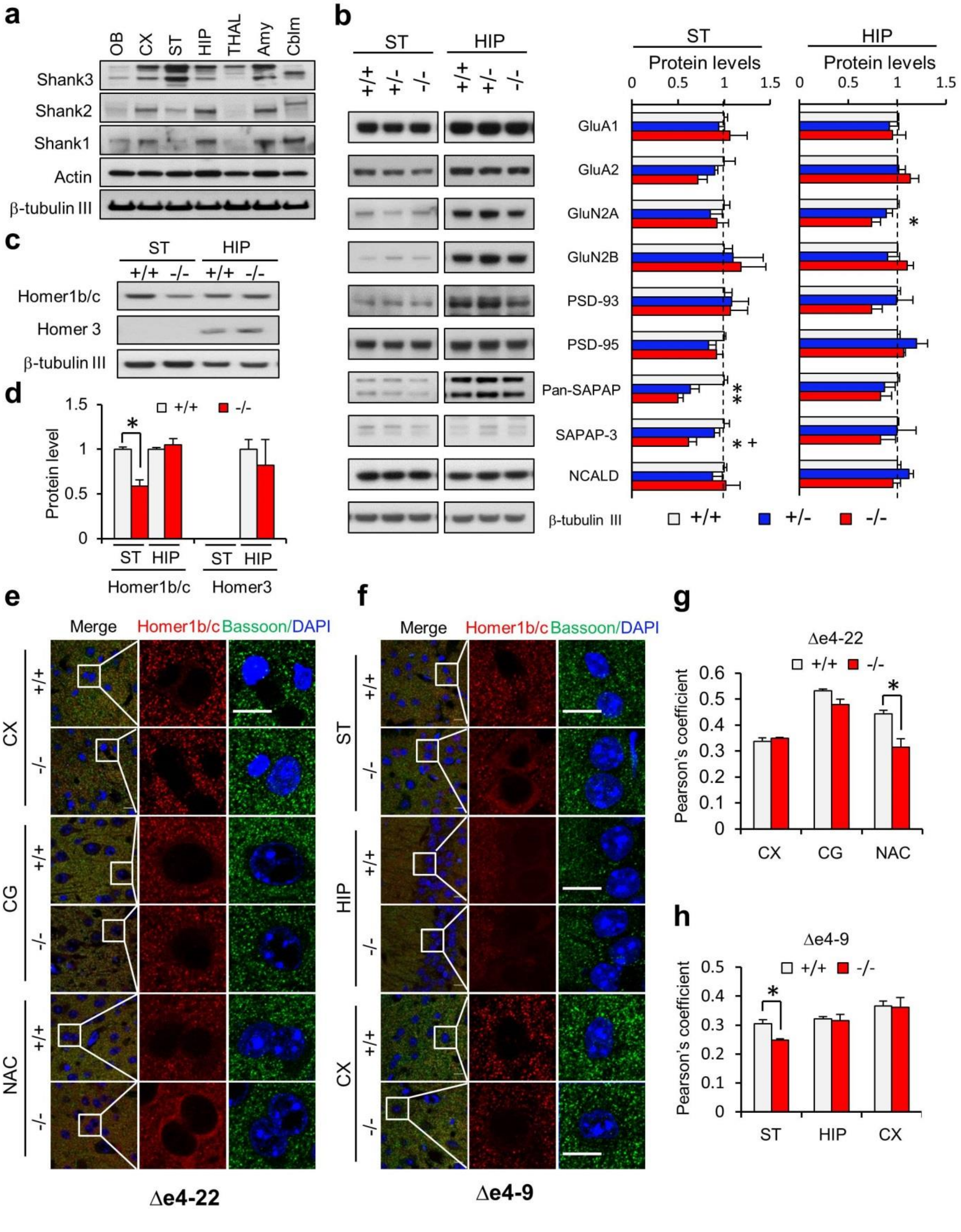

a
OB CX ST HIP THAL Amy Cblm
Shank3
Shank2
Shank1
Actin
β-tubulin III
b
ST HIP
+/+ +/- -/- +/+ +/- -/-
ST Protein levels
HIP Protein levels
0 0.5 1 1.5
GluA1
GluA2
GluN2A
GluN2B
PSD-93
PSD-95
Pan-SAPAP
SAPAP-3
NCALD
β-tubulin III
+/+ +/- -/-
c
ST HIP
+/+ -/- +/+ -/-
Homer1b/c
Homer 3
β-tubulin III
d
+/+ -/-
Protein level
Homer1b/c Homer3
e
Merge Homer1b/c Bassoon/DAPI
CX CG NAC
Δe4-22
f
ST HIP CX
Δe4-9
g
Δe4-22
Pearson's coefficient
CX CG NAC
h
Δe4-9
Pearson's coefficient
ST HIP CX

Supplementary Figure 6. Brain region-specific changes of PSD proteins in ∆e4-22 and ∆e4-9 mice. (a) The expression profile of Shank family proteins in different mouse brain regions by immunoblot analysis. In comparison to other regions, Shank3 is expressed at a higher level in striatum than Shank1 or Shank2. OB: olfactory bulb, CX: cortex, ST: striatum, HIP: hippocampus, THAL: thalamus, Amy: amygdala, Cblm: cerebellum. **(b)** Changes in PSD proteins in ST and HIP of ∆e4-22 mice. Pan-SAPAP, and SAPAP-3 in striatum and GluN2A in hippocampus were significantly decreased in ∆e4-22$^{-/-}$ (-/-) mice. *p<0.05, from ∆e4-22$^{+/+}$ (+/+); ^{+}p<0.05 from ∆e4-22$^{+/-}$ (+/-); one-way ANOVA with *post-hoc* Tukey test; n=7 for GluN2A, SAPAP-3, n=4 for pan-SAPAP, n ≥ 4 mice/genotype for all other proteins. **(c-d)** Mild decrease of Homer1b/c in the PSD in the ST, but not in the HIP of *Shank3*$^{\Delta e4\text{-}9\text{-}/\text{-}}$ mice. *p<0.001; t-test; n=5 mice/genotype. **(e)** Immunostaining of Homer1b/c (*red*) and a presynaptic marker Bassoon (*green*) were performed using brain slices from the neocortex (CX), cingulate cortex (CG), and nucleus accumbens (NAC) of -/- and +/+ mice. An accumulation of Homer1b/c in the cytoplasm was observed in the NAC, but not in the CG or CX of -/- mice. **(f)** Immunostaining of Homer1b/c (*red*) and Bassoon (*green*) in brain slices from ST, HIP, and CX of ∆e4-9$^{+/+}$ and ∆e4-9$^{-/-}$ mice. A mild accumulation of cytosolic Homer1b/c was observed in the ST, but not in the HIP (CA1 region) or CX. Scale bar: 10 µm. **(g)** Pearson's analysis revealed a reduced correlation between Homer1b/c and Bassoon in the NAC of ∆e4-22$^{-/-}$ mice. *p<0.01; t-test, n=5-6 slices/genotype. **(h)** Pearson's analysis revealed a mild reduction in Homer1b/c-Bassoon correlation in the ST but not in the HIP or CX of ∆e4-9 $^{-/-}$ mice. *p<0.002, t-test; n=8 slices/genotype. Scale bar: 10 µm. Experiments for western blots were repeated at least three times. All data are expressed as means ± SEM.

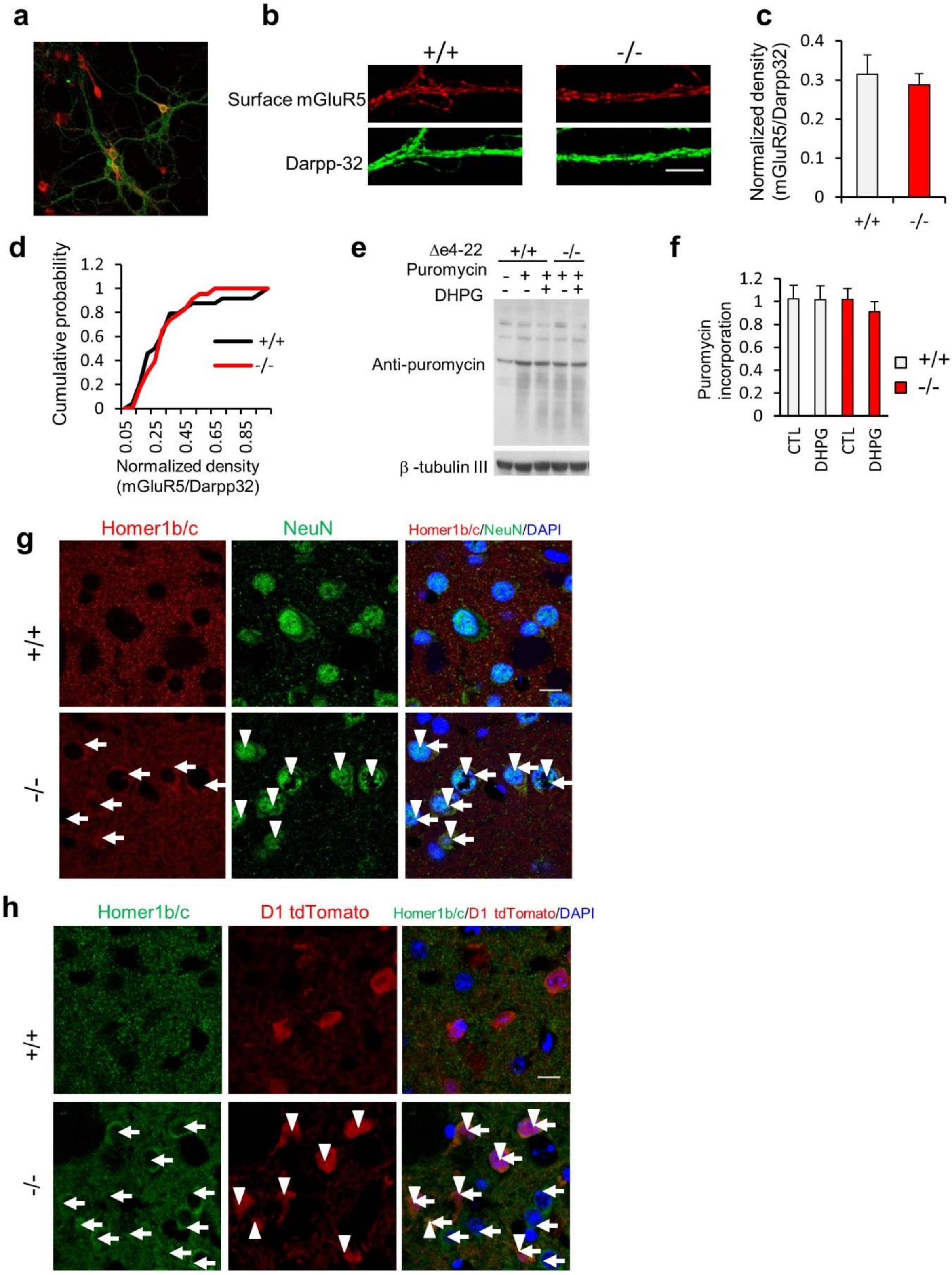

a
b
+/+
-/-
Surface mGluR5
Darpp-32
c
Normalized density (mGluR5/Darpp32)
0.4
0.3
0.2
0.1
0
+/+
-/-
d
Cumulative probability
1.2
1
0.8
0.6
0.4
0.2
0
+/+
-/-
0.05
0.25
0.45
0.65
0.85
Normalized density (mGluR5/Darpp32)
e
Δe4-22
+/+
-/-
Puromycin
DHPG
Anti-puromycin
β -tubulin III
f
Puromycin incorporation
1.2
1
0.8
0.6
0.4
0.2
0
CTL
DHPG
CTL
DHPG
+/+
-/-
g
Homer1b/c
NeuN
Homer1b/c/NeuN/DAPI
+/+
-/-
h
Homer1b/c
D1 tdTomato
Homer1b/c/D1 tdTomato/DAPI
+/+
-/-

Supplementary Figure 7. Surface mGluR5 and protein synthesis in striatum of Δe4-22$^{-/-}$ mice are not altered. **(a)** Immunostaining of surface mGluR5 protein (*red*) in corticostriatal co-cultures using an mGluR5 antibody that detects the extracellular domain of mGluR5. Darpp-32 (*green*) was used as a marker to identify MSNs. **(b)** Representative images of mGluR5 surface immunostaining in dendritic spines of Darpp-32 positive neurons of Δe4-22$^{+/+}$ (+/+) and Δe4-22$^{-/-}$ (-/-) mice. **(c-d)** The normalized density **(c)** and cumulative probability **(d)** of surface mGluR5 was not altered in -/- compared to +/+ mice. +/+, n=24; -/-, n=23. **(e-f)** The rate of protein synthesis was not altered in striatal slices from -/- mice. **(e)** Representative immunoblot images for puromycin incorporation in striatal brain slices. The slices were incubated with or without 100 μM DHPG for 5 min followed by incubation with or without puromycin for 45 min at 32ºC. **(f)** Quantification of puromycin intensity indicates that there was no difference in protein synthesis between genotypes. n=9 slices from 3 mice for each group. **(g)** Co-immunostaining of Homer1b/c (*red*) and NeuN (*green*) showed all NeuN positive neurons bear cytosolic Homer accumulation in -/- mice. Homer is indicated by the arrow, NeuN indicated by the arrow-head. Scale bar, 10 μm. **(h)** Immunostaining of Homer1b/c (*green*) in D1 tdTomato transgenic/Δe4-22$^{-/-}$ mice showed that cytosolic Homer accumulation is not restricted to D1 positive neurons. Homer is indicated by the arrow, D1 tdTomato by the arrow-head. Scale bar, 10 μm. Experiments for western blot were repeated two times. All data are expressed as means ± SEM.

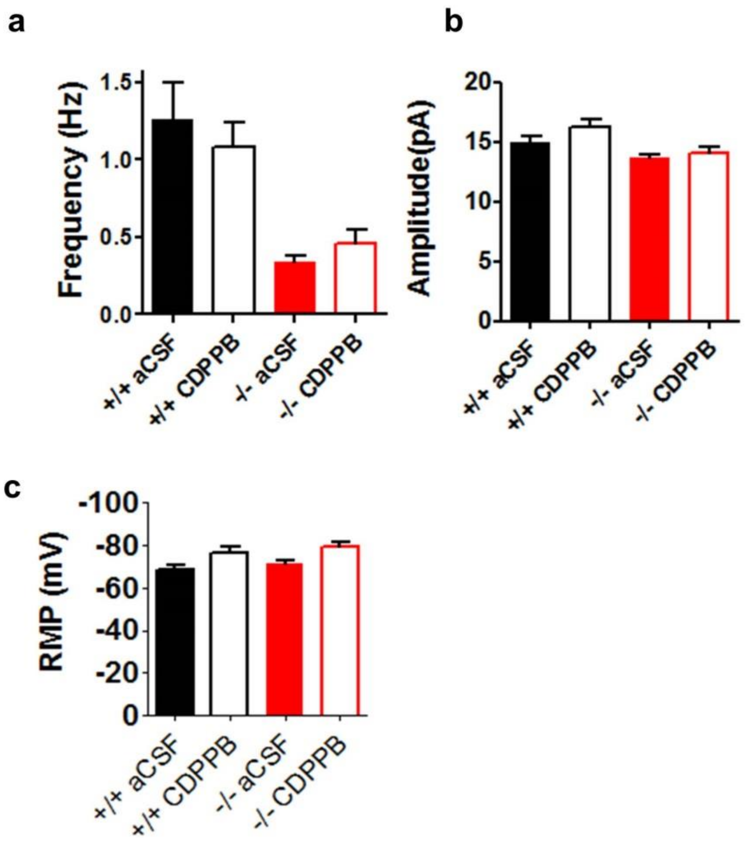

Supplementary Figure 8. (a-b) CDPPB does not change the frequency (a) and amplitude (b) of sEPSCs in MSNs of *Shank3* mice. Planned comparison, t-test; n=8 each genotype. **(c)** CDPPB does not alter resting membrane potential (RMP) in *Shank3* mice. ANOVA; n=12 each genotype. All data are expressed as means ± SEM.

Figure 1d

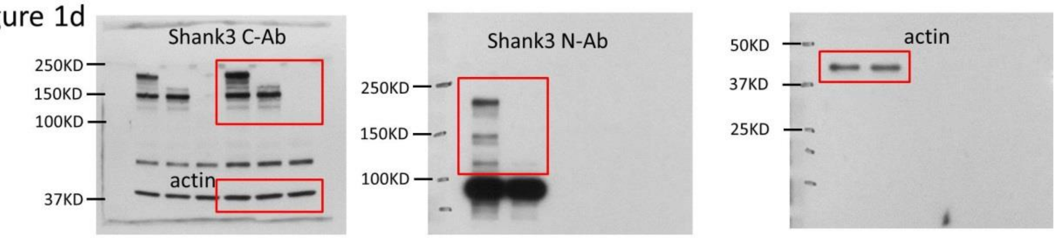

Figure 6a

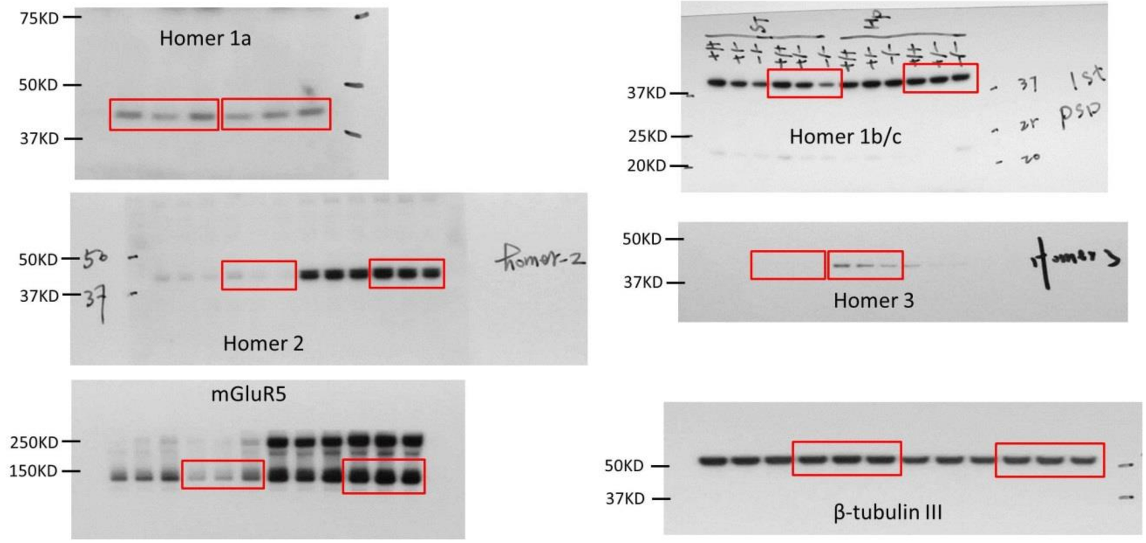

Figure 6e

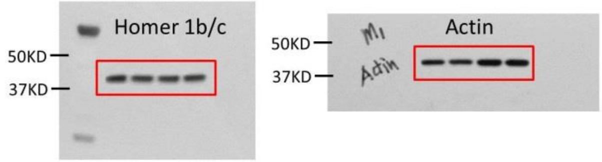

Figure 6g

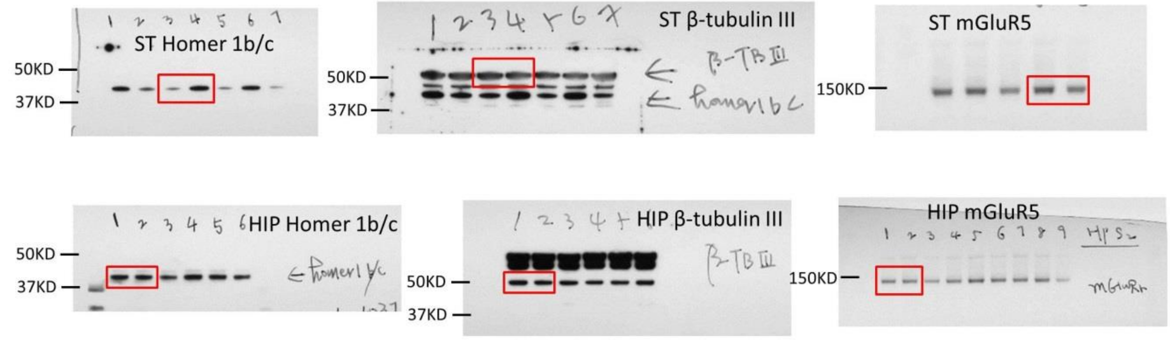

Figure 7f

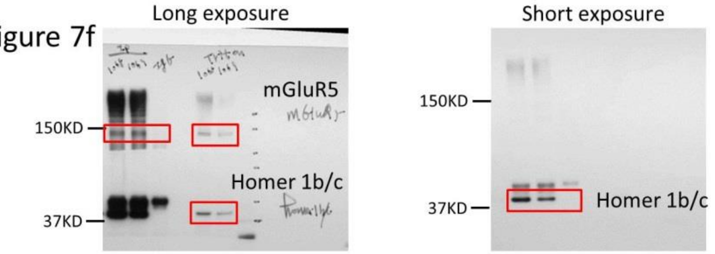

Figure 7g

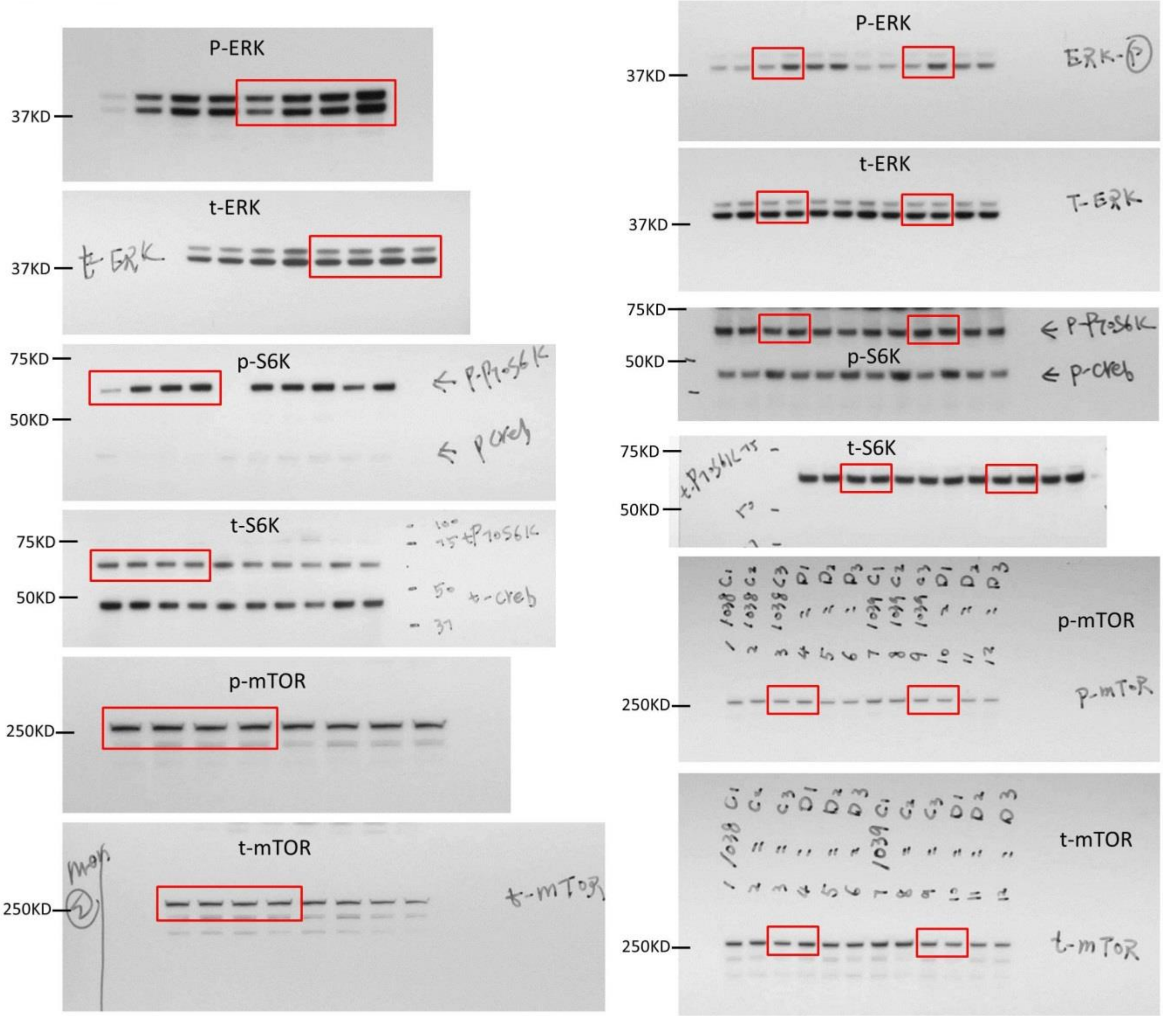

Figure 8i

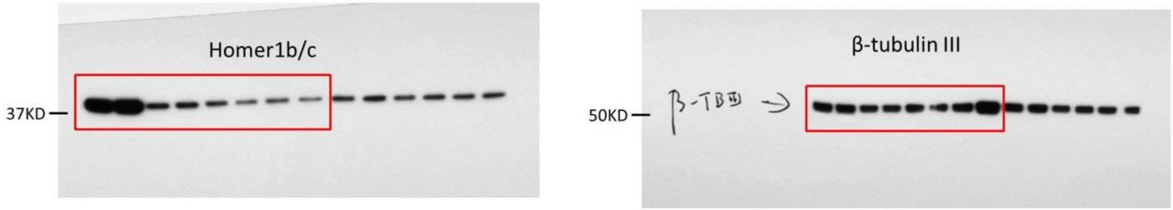

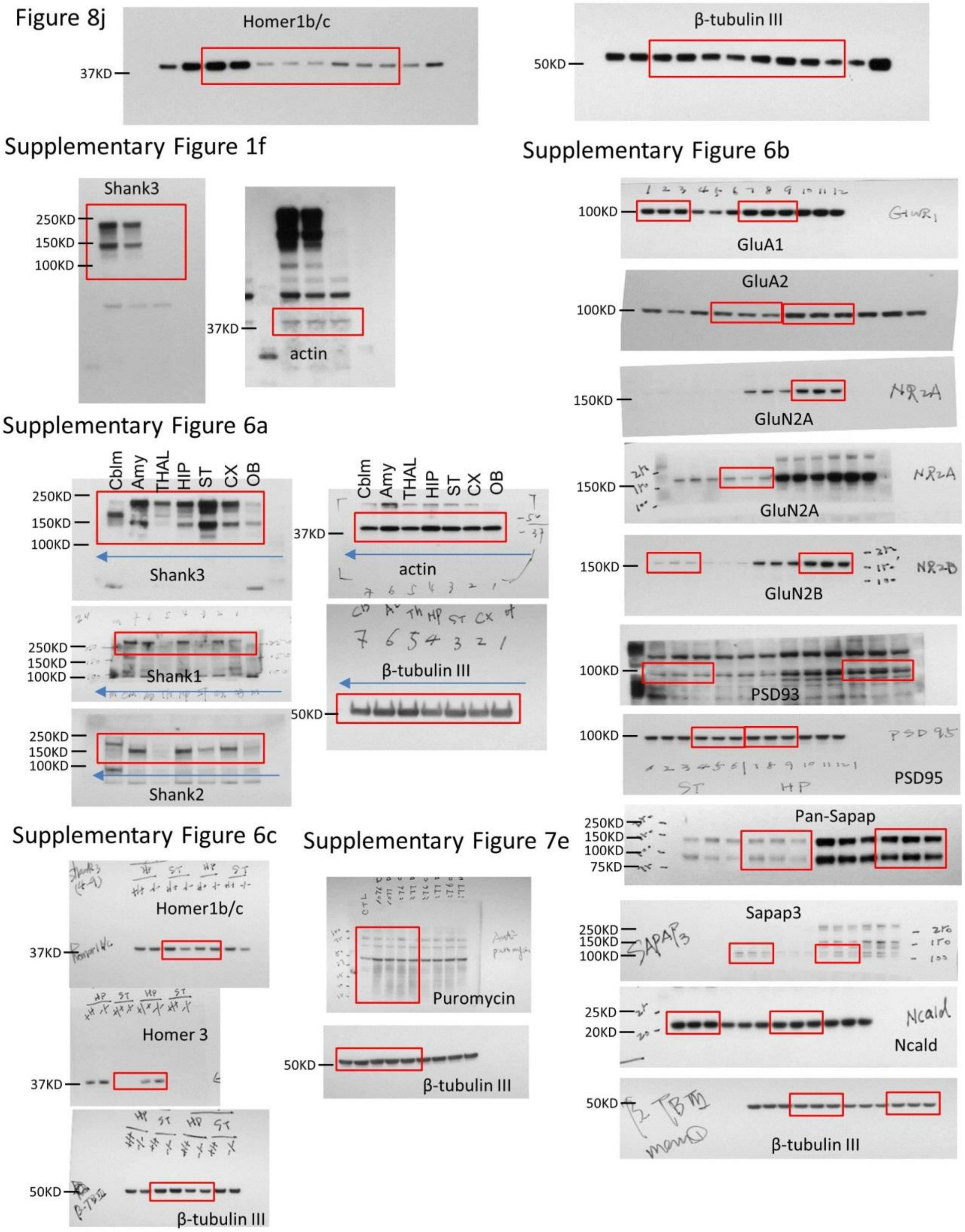

Supplementary Figure 9. Original full scans of Western blots related to respective figures as indicated.

Supplementary Table 1. The cohorts used and the order of behavioral testing of *Shank3* ∆e4-22 mice

Cohort 1	Cohort 2	Cohort 3	Cohort 4	Cohort 5	Cohort 6	Cohort 7	Cohort 8	Cohort 9	Cohort 10
N=9 per genotype	N=10-11 per genotype	N=10-11 per genotype	N=9-18 per genotype	N=11-13 per genotype	N=8-9 per genotype	N=11-13 per genotype	N=11-14 per genotype	N=10-12 per genotype per treatment	N=8-12 per genotype per treatment
Light-dark box	Spray test	Open field	Neonatal milestones	Light- dark box	Olfactory habituation-dishabit-uation	Instru-mental learning	Forced social interact-tion test	Grooming in the home cage (MPEP)	Grooming in the home cage (CDPPB)
Hole-board	Sociability	Novel object recognition memory (aborted)	Pup USVs	Novel object chambers to confirm escaping	Adult USVs (males only)			Open field (MPEP)	Open field (CDPPB)
Rotarod	Social Transmission of food preference (aborted)	Social hierarchy	Nest preference test	Olfaction preference	Social dyadic (males only)				Instrumental learning (CDPPB)
Grip strength	Social hierarchy	Fear conditioning		Morris water maze					
Prepulse inhibition	Fear conditioning			Visible platform water maze					
Spray test				Adult USVs (males only)					
Sociability				Social dyadic (males only)					

Supplementary Table 2: Results of general behavioral testing of *Shank3* ∆e4-22 mice

Test (number per genotype)	*Shank3*$^{\Delta e4\text{-}22+/+}$	*Shank3*$^{\Delta e4\text{-}22+/-}$	*Shank3*$^{\Delta e4\text{-}22-/-}$	*p*-value
Developmental Milestones (n=9-18)				
Ears open	100.0 %	94.4 %	66.7%	*p*<0.04[i]
Rooting reflex	77.8 %	66.7 %	58.3 %	ns[ii]
Cross-extensor reflex	88.9 %	88.9 %	75.0 %	ns[ii]
Negative geotaxis	77.8 %	55.6 %	58.3 %	ns[ii]
Adult paw position	55.6 %	27.8 %	8.3 %	*p*=0.06[iii]
Self-righting	88.9 %	61.1 %	75.0 %	ns[ii]
Milk present	66.7 %	72.2 %	66.7 %	ns[ii]
Olfactory Discrimination (n=8-13)				
Preference for urine over saline	0.3 ± 0.1	0.6 ± 0.1	0.4 ± 0.1	ns[ii]
Hole-board Exploration (n=7-8)				
Total nose-pokes	43.4 ± 4.5	36.4 ± 3.2	32.3 ± 3.9	ns[ii]
Neonatal Nest Preference (n=8-10)				
Latency to nest (1-way choice) (s)	24.2 ± 4.6	38.1 ± 6.3	23.4 ± 5.1	ns[ii]
Social Hierarchy (n=15-18)				
Male agonistic behaviors	7.0 ± 1.6	-------	5.0 ± 1.9	ns[ii]
Female agonistic behaviors	7.6 ± 1.7	-------	6.3 ± 1.3	ns[ii]
Sociability (n=9)				
Time (s) with stimuli in NS-NS	61.9 ± 4.5	69.2 ± 6.80	68.9 ± 13.5	ns[ii]
Time (s) with stimuli in NS-S1	76.6 ± 8.2	71.0 ± 12.2	71.4 ± 11.6	ns[ii]
Time (s) with stimuli in S1-S2	66.9 ± 9.6	70.9 ± 16.5	62.1 ± 11.8	ns[ii]
Contacts with stimuli in NS-NS	92.9 ± 4.6	109.6 ± 8.8	99.3 ± 19.5	ns[ii]
Contacts with stimuli in NS-S1	98.4 ± 14.1	89.5 ± 13.1	96.9 ± 21.1	ns[ii]
Contacts with stimuli in S1-S2	72.1 ± 10.2	97.1 ± 16.7	82.6 ± 15.2	ns[ii]
Social Dyadic (n=10-15)				
Bi-directional episodes	20.3 ± 2.7	22.0 ± 2.7	20.7 ± 2.0	ns[ii]
Non-reciprocated episodes	3.6± 0.8	3.9 ± 1.0	12.8 ± 2.4	*p*<0.001[iv]
Ultrasonic Vocalizations (n=10-13)				
Adult call amplitude (dB)	-39.4 ± 1.4	-39.9 ± 1.7	-45.1± 1.7	*p*<0.04[v]
Adult call frequency (kHz)	69.4 ± 0.8	70.1 ± 1.0	69.1 ± 2.7	ns[ii]
Grip Strength (n=9)				
Forepaws (grams of force)	60.9 ± 2.1	59.9 ± 2.0	63.3 ± 2.8	ns[ii]
Whole body (grams of force)	114.8 ± 1.6	117.2 ± 2.2	122.8 ±3.8	ns[ii]

Open Field Exploration (n=6-10)				
Vertical activity (beam-breaks)	488.5 ± 115.7	420.6 ± 58.5	248.1 ± 73.2	ns[ii]
Center time (s)	2398.6 ± 72.3	1730.0 ± 172.8	1641.4 ± 156.4	$p<0.01$[vi]
Prepulse Inhibition (n=9)				
Null activity (mAmp)	2.7 ± 0.7	1.89 ± 0.4	2.00 ± 0.7	ns[ii]
Startle reactivity (mAmp)	377.7 ± 32.5	239.39 ± 41.4	190.51 ± 41.2	$p<0.03$[vii]
% PPI at 4 dB	5.6 ± 3.5	19.10 ± 3.7	16.91 ± 4.1	ns[ii]
% PPI at 8 dB	18.3 ± 6.4	36.32 ± 6.3	31.55 ± 6.1	ns[ii]
% PPI at 12 dB	34.8 ± 6.1	53.45 ± 5.3	52.32 ± 5.5	ns[ii]
Visible Water Maze (n=11-13)				
Day 1 swim time (s)	28.2 ± 1.9	23.52 ± 2.3	31.38 ± 2.5	$p<0.03$[viii]
Day 2 swim time (s)	18.4 ± 2.1	16.27 ± 2.0	22.63 ± 2.2	$p<0.03$[viii]
Day 3 swim time (s)	9.6 ± 1.2	13.55 ± 1.5	13.99 ± 1.5	$p<0.03$[viii]
Conditioned Fear (n=13-17)				
% Freezing (conditioning pre-shock)	27.0 ± 2.6	-------	29.96 ± 2.5	ns[ii]
% Freezing (conditioning post-shock)	49.1 ± 4.7	-------	40.75 ± 7.0	ns[ii]
% Freezing in contextual fear	60.8 ± 2.5	-------	69.90 ± 3.2	$p<0.04$[ix]
% Freezing in cued fear conditioning	46.3 ± 4.2	-------	47.08 ± 6.1	ns[ii]

[i] Chi-Square analyses, ears open: $x^2_{(2,N=39)} = 6.69$, $p<0.035$

[ii] ns refers to $p > 0.05$

[iii] Chi-Square analyses, paw position: $x^2_{(2,N=39)} = 5.67$, $p<0.059$

[iv] ANOVA: $F_{(2,37)}=9.05$, $p<0.001$; *post-hoc* tests: *Shank3*$^{\Delta 4\text{-}22+/+}$ versus *Shank3*$^{\Delta 4\text{-}22-/-}$ mice ($p<0.001$), *Shank3*$^{\Delta 4\text{-}22+/-}$ versus *Shank3*$^{\Delta 4\text{-}22-/-}$ mice ($p<0.001$)

[v] ANOVA: $F_{(2,34)}=3.75$, $p<0.04$; *post-hoc* tests: *Shank3*$^{\Delta 4\text{-}22+/+}$ versus *Shank3*$^{\Delta 4\text{-}22-/-}$ mice ($p<0.03$), *Shank3*$^{\Delta 4\text{-}22+/-}$ versus *Shank3*$^{\Delta 4\text{-}22-/-}$ mice ($p<0.03$)

[vi] ANOVA: $F_{(2,23)}=5.71$, $p<0.01$; *post-hoc* tests: *Shank3*$^{\Delta 4\text{-}22+/+}$ versus *Shank3*$^{\Delta 4\text{-}22-/-}$ mice ($p<0.05$), *Shank3*$^{\Delta 4\text{-}22+/-}$ versus *Shank3*$^{\Delta 4\text{-}22-/-}$ mice ($p<0.05$)

[vii] ANOVA: $F_{(2,21)}=4.31$, $p<0.03$; *post-hoc* tests: *Shank3*$^{\Delta 4\text{-}22+/+}$ versus *Shank3*$^{\Delta 4\text{-}22-/-}$ mice ($p<0.03$), *Shank3*$^{\Delta 4\text{-}22+/-}$ versus *Shank3*$^{\Delta 4\text{-}22-/-}$ mice ($p=1.00$); *Shank3*$^{\Delta 4\text{-}22+/+}$ versus *Shank3*$^{\Delta 4\text{-}22+/-}$ mice ($p=0.13$)

[viii] RMANOVA: within subjects effect of day $F_{(2,64)}=51.07$, $p<0.001$; between subjects effect of genotype $F_{(2,32)}=4.41$, $p<0.02$; day x genotype interaction $F_{(4,64)}=1.66$, $p=0.17$; Bonferroni-corrected post-hoc comparisons of *Shank3*$^{\Delta 4\text{-}22-/-}$ mice versus *Shank3*$^{\Delta 4\text{-}22+/+}$ or $^{+/-}$ all exceeded $p=0.05$ for individual test days

[ix] Independent samples t-test: $T_{(28)} = -2.27$, $p<0.04$

Supplementary Table 3. Statistics for power, coherence, and local connectivity measures of multi-circuit *in vivo* recording of *Shank3* Δe4-22 mice

Statistics for power measures for Figure 3b[i]

		N	degrees of freedom	Genotype		Test	
		+/+ / -/-		F	*p*	F	*p*
NAC	2-7Hz	14/11	1,23	7.388	0.012	1.699	0.205
NAC	7-11Hz	14/11	1,23	12.169	0.002	4.963	0.036
NAC	15-30Hz	14/11	1,23	1.183	0.288	45.620	6.9E-07
NAC	30-55Hz	14/11	1,23	0.032	0.859	3.560	0.072
V_HIP	2-7Hz	13/11	1,22	1.508	0.232	38.021	0.000
V_HIP	7-11Hz	13/11	1,22	1.419	0.246	0.917	0.349
V_HIP	15-30Hz	13/11	1,22	0.970	0.335	16.117	0.001
V_HIP	30-55Hz	13/11	1,22	1.525	0.230	54.851	2.1E-07
CG_CX	2-7Hz	14/11	1,23	0.678	0.419	3.263	0.084
CG_CX	7-11Hz	14/11	1,23	0.269	0.609	50.835	2.9E-07
CG_CX	15-30Hz	14/11	1,23	0.028	0.869	55.684	1.4E-07
CG_CX	30-55Hz	14/11	1,23	0.009	0.925	26.725	3.1E-05
THAL	2-7Hz	14/11	1,23	1.421	0.245	8.702	0.007
THAL	7-11Hz	14/11	1,23	0.062	0.806	87.212	2.7E-09
THAL	15-30Hz	14/11	1,23	0.062	0.806	98.540	8.8E-10
THAL	30-55Hz	14/11	1,23	0.006	0.941	43.798	9.4E-07
PRL_CX	2-7Hz	14/11	1,23	0.539	0.470	2.311	0.142
PRL_CX	7-11Hz	14/11	1,23	0.010	0.921	28.175	2.2E-05
PRL_CX	15-30Hz	14/11	1,23	0.506	0.484	41.209	1.5E-06
PRL_CX	30-55Hz	14/11	1,23	0.763	0.391	62.195	5.5E-08

[i] Statistical table for power measures analyzed using a mixed model ANOVA. Comparisons that survived Bonferroni-correction for multiple comparisons are highlighted in pink (p<0.05 out of 40 comparisons).

Statistics for coherence measure for Figure 3c-d[i]

		N	degrees of freedom	Genotype		Test	
		+/+ / -/-		F	*p*	F	*p*
NAC x V_HIP	2-7Hz	13/11	1,22	0.056	0.816	2.683	0.116
NAC x V_HIP	7-11Hz	13/11	1,22	0.032	0.859	22.521	9.8E-05
NAC x V_HIP	15-30Hz	13/11	1,22	1.410	0.248	17.210	4.2E-04
NAC x V_HIP	30-55Hz	13/11	1,22	2.159	0.156	1.135	0.298
NAC x CG_CX	2-7Hz	14/11	1,23	1.602	0.218	15.228	0.001
NAC x CG_CX	7-11Hz	14/11	1,23	1.525	0.229	127.994	7.1E-11
NAC x CG_CX	15-30Hz	14/11	1,23	0.642	0.431	56.288	1.3E-07
NAC x CG_CX	30-55Hz	14/11	1,23	1.557	0.225	3.007	0.096
NAC x THAL	2-7Hz	14/11	1,23	11.803	0.002	29.322	1.7E-05
NAC x THAL	7-11Hz	14/11	1,23	4.324	0.049	64.372	4.1E-08
NAC x THAL	15-30Hz	14/11	1,23	0.088	0.769	17.238	3.9E-04
NAC x THAL	30-55Hz	14/11	1,23	0.183	0.673	0.024	0.877
NAC x PRL_CX	2-7Hz	14/11	1,23	0.017	0.898	11.086	0.003
NAC x PRL_CX	7-11Hz	14/11	1,23	0.120	0.732	38.690	2.4E-06
NAC x PRL_CX	15-30Hz	14/11	1,23	0.392	0.537	56.052	1.3E-07
NAC x PRL_CX	30-55Hz	14/11	1,23	0.889	0.356	1.679	0.208
V_HIP x CG_CX	2-7Hz	13/11	1,22	0.463	0.503	13.046	0.002
V_HIP x CG_CX	7-11Hz	13/11	1,22	0.359	0.555	25.590	4.6E-05
V_HIP x CG_CX	15-30Hz	13/11	1,22	0.001	0.973	3.577	0.072
V_HIP x CG_CX	30-55Hz	13/11	1,22	0.354	0.558	0.176	0.679
V_HIP x THAL	2-7Hz	13/11	1,22	0.016	0.900	0.217	0.646
V_HIP x THAL	7-11Hz	13/11	1,22	0.026	0.873	13.721	0.001
V_HIP x THAL	15-30Hz	13/11	1,22	1.284	0.269	0.895	0.354
V_HIP x THAL	30-55Hz	13/11	1,22	2.501	0.128	0.542	0.470
V_HIP x PRL_CX	2-7Hz	13/11	1,22	0.162	0.691	4.625	0.043
V_HIP x PRL_CX	7-11Hz	13/11	1,22	0.018	0.893	32.607	9.6E-06
V_HIP x PRL_CX	15-30Hz	13/11	1,22	1.614	0.217	11.062	0.003
V_HIP x PRL_CX	30-55Hz	13/11	1,22	7.368	0.013	0.372	0.548
CG_CX x THAL	2-7Hz	13/11	1,22	8.045	0.009	26.591	3.2E-05
CG_CX x THAL	7-11Hz	14/11	1,23	1.335	0.260	67.226	2.8E-08
CG_CX x THAL	15-30Hz	14/11	1,23	0.086	0.773	22.934	7.9E-05
CG_CX x THAL	30-55Hz	14/11	1,23	0.150	0.702	3.699	0.067
CG_CX x PRL_CX	2-7Hz	14/11	1,23	1.224	0.280	3.887	0.061
CG_CX x PRL_CX	7-11Hz	14/11	1,23	0.658	0.426	40.567	1.7E-06
CG_CX x PRL_CX	15-30Hz	14/11	1,23	0.171	0.683	47.690	4.9E-07
CG_CX x PRL_CX	30-55Hz	14/11	1,23	0.122	0.730	2.099	0.161
THAL x PRL_CX	2-7Hz	14/11	1,23	5.339	0.030	22.227	9.5E-05
THAL x PRL_CX	7-11Hz	14/11	1,23	0.502	0.486	45.350	7.2E-07
THAL x PRL_CX	15-30Hz	14/11	1,23	0.722	0.404	23.814	6.3E-05
THAL x PRL_CX	30-55Hz	14/11	1,23	3.967	0.058	2.036	0.167

[i] Statistical table for coherence measures analyzed using a mixed model ANOVA. Comparisons that survived Bonferroni-correction for multiple comparisons are highlighted in pink ($p<0.05$ out of 40 comparisons).

Statistics for coherence measure for Figure 3e[i]

		N	Degrees of freedom	Genotype × Test	
		+/+ / -/-		F	*p*
NAC x V_HIPP	7-11Hz	13/11	1,22	3.136	0.090
NAC x V_HIPP	15-30Hz	13/11	1,22	1.476	0.237
NAC x CG_CX	2-7Hz	14/11	1,23	6.792	0.016
NAC x CG_CX	7-11Hz	14/11	1,23	11.203	0.003
NAC x CG_CX	15-30Hz	14/11	1,23	0.000	0.992
NAC x THAL	2-7Hz	14/11	1,23	2.976	0.098
NAC x THAL	7-11Hz	14/11	1,23	12.206	0.002
NAC x THAL	15-30Hz	14/11	1,23	2.576	0.122
NAC x PRL_CX	7-11Hz	14/11	1,23	0.042	0.839
NAC x PRL_CX	15-30Hz	14/11	1,23	1.622	0.216
V_HIP x CG_CX	7-11Hz	13/11	1,22	1.840	0.189
V_HIP x THAL	7-11Hz	13/11	1,22	0.827	0.373
V_HIP x PRL_CX	7-11Hz	13/11	1,22	0.258	0.617
CG_CX x THAL	2-7Hz	14/11	1,23	2.966	0.098
CG_CX x THAL	7-11Hz	14/11	1,23	3.531	0.073
CG_CX x THAL	15-30Hz	14/11	1,23	0.223	0.641
CG_CX x PRL_CX	7-11Hz	14/11	1,23	0.130	0.722
CG_CX x PRL_CX	15-30Hz	14/11	1,23	0.303	0.588
THAL x PRL_CX	2-7Hz	14/11	1,23	0.819	0.375
THAL x PRL_CX	7-11Hz	14/11	1,23	0.006	0.938
THAL x PRL_CX	15-30Hz	14/11	1,23	0.133	0.718

[i] Statistical table for genotype by test condition interaction for coherence measures that displayed significant test-condition effects using mixed model ANOVA. Comparisons that survived FDR-correction for multiple comparisons are highlighted in pink.

Statistics for unit firing rates for Figure 3f[i]

	N	Degrees of freedom	Genotype		Test		Genotype × Test	
	+/+ / -/-		F	*p*	F	*p*	F	*p*
NAC	109/158	1,265	13.516	2.86E-04	16.022	8.13E-05	0.484	0.487
CG_CX	91/76	1,165	2.0037	0.159	47.142	1.28E-10	0.4812	0.489
THAL	21/33	1,52	3.121	0.083	38.570	8.94E-08	0.11	0.742

[i] Statistical table for genotype by test condition interaction for single unit firing rates analyzed using a mixed model ANOVA, with significant effects highlighted in pink.

Statistics for local connectivity measures for Figure 3g[i]

		N	Degrees of freedom	Genotype		Test		Genotype × Test	
		+/+ / -/-		F	*p*	F	*p*	F	*p*
NAC	15-30Hz	14/11	1,23	0.373	0.548	2.596	0.121	2.594	0.121
NAC	30-55Hz	14/11	1,23	1.194	0.286	35.808	4.2E-06	0.481	0.495
CG_CX	15-30Hz	14/11	1,23	0.403	0.532	0.175	6.8E-01	5.147	0.033
CG_CX	30-55Hz	14/11	1,23	0.459	0.505	4.079	0.055	0.004	0.949
THAL	15-30Hz	14/11	1,23	0.162	0.691	0.260	0.615	0.048	0.829
THAL	30-55Hz	14/11	1,23	0.618	0.440	6.430	1.8E-02	1.146	0.296

i Statistical table for genotype by test condition interaction for cross frequency phase coupling measures. A significant test effect was found for NAC 30-55Hz coupling, highlighted pink. No significant genotype or genotype x test effect was observed following FDR-correction for multiple comparisons.

Statistics for temporal offset for Figure 3h[i]

		N +/+ / -/-	*p*
NAC x CG_CX	2-7Hz	14/11	0.286
NAC x CG_CX	7-11Hz	14/11	0.180
NAC x CG_CX	15-30Hz	14/11	0.565
NAC x THAL	2-7Hz	14/11	0.366
NAC x THAL	7-11Hz	14/11	0.015
NAC x THAL	15-30Hz	14/11	0.603
CG_CX x THAL	2-7Hz	14/11	0.848
CG_CX x THAL	7-11Hz	14/11	0.106
CG_CX x THAL	15-30Hz	14/11	0.427

[i] Statistical table for temporal delays that displayed significant genotype effects using FDR-corrected rank sum test, highlighted in pink.

Statistics for local connectivity measures for Supplemental Figure 3[i]

	N	Degrees of freedom	Genotype		Test		Genotype × Test	
	+/+ / -/-		F	*p*	F	*p*	F	*p*
NAC	105/149	1,252	0.689	0.407	3.260	0.072	3.306	0.070
V_HIP	15/9	1,22	0.251	0.622	1.960	0.175	0.246	0.625
CG_CX	89/62	1,149	0.047	0.829	1.010	0.317	0.018	0.893
THAL	21/25	1,44	0.125	0.725	1.189	0.281	1.215	0.276
PRL_CX	51/26	1,75	2.529	0.116	3.566	0.063	0.217	0.643

[i] Statistical table for genotype by test condition interaction for single unit phase locking measures. No significant effects were observed following FDR-correction for multiple comparisons.

Supplementary Table 4. Statistics for coherence measures in C57B6/J mice (n=12).

Region	Frequency	*p*-value
'NAC x V_HIP'	2-7Hz	0.9697
'NAC x V_HIP'	7-11Hz	0.2334
'NAC x V_HIP'	15-30Hz	0.3804
'NAC x V_HIP'	30-50Hz	0.0342
'NAC x CG_CX'	2-7Hz	0.2334
'NAC x CG_CX'	7-11Hz	0.0005
'NAC x CG_CX'	15-30Hz	0.0210
'NAC x CG_CX'	30-50Hz	0.0010
'NAC x THAL'	2-7Hz	0.0034
'NAC x THAL'	7-11Hz	0.0005
'NAC x THAL'	15-30Hz	0.5693
'NAC x THAL'	30-50Hz	0.0005
'NAC x PRL_CX'	2-7Hz	0.4697
'NAC x PRL_CX'	7-11Hz	0.0005
'NAC x PRL_CX'	15-30Hz	0.1099
'NAC x PRL_CX'	30-50Hz	0.0005
'V_HIP x CG_CX'	2-7Hz	0.7334
'V_HIP x CG_CX'	7-11Hz	0.3804
'V_HIP x CG_CX'	15-30Hz	0.7910
'V_HIP x CG_CX'	30-50Hz	0.2661
'V_HIP x THAL'	2-7Hz	1.0000
'V_HIP x THAL'	7-11Hz	0.0522
'V_HIP x THAL'	15-30Hz	0.5186
'V_HIP x THAL'	30-50Hz	0.0024
'V_HIP x PRL_CX'	2-7Hz	0.8501
'V_HIP x PRL_CX'	7-11Hz	0.6221
'V_HIP x PRL_CX'	15-30Hz	0.5693
'V_HIP x PRL_CX'	30-50Hz	0.0923
'CG_CX x THAL'	2-7Hz	0.0068
'CG_CX x THAL'	7-11Hz	0.0049
'CG_CX x THAL'	15-30Hz	0.2661
'CG_CX x THAL'	30-50Hz	0.0005
'CG_CX x PRL_CX'	2-7Hz	0.5186
'CG_CX x PRL_CX'	7-11Hz	0.0005
'CG_CX x PRL_CX'	15-30Hz	0.0161
'CG_CX x PRL_CX'	30-50Hz	0.0049
'THAL x PRL_CX'	2-7Hz	0.0024
'THAL x PRL_CX'	7-11Hz	0.0049
'THAL x PRL_CX'	15-30Hz	0.4697
'THAL x PRL_CX'	30-50Hz	0.0005

Supplementary Table 5. Summary of regional volumetric changes in *Shank3* ∆e4-22 mice

	$Shank3^{\Delta e4\text{-}22\text{-}/\text{-}}$ (mean±SEM)	CTRL (mean±SEM)	p	CI[1]	CI[2]	t	Cohen d	Difference (%)
Globus Pallidus (GP)	0.54±0.02	0.46±0.01	0.002	0.04	0.13	3.87	3.87	17.79
Anterior Pretectal Nuclei (APT)	0.16±0.00	0.14±0.00	0.004	0.01	0.04	3.62	3.62	16.42
Deep Mesencephalic Nuclei (DpMe)	0.59±0.01	0.52±0.01	0.003	0.03	0.10	3.77	3.77	12.01
Interpeduncular Nucleus (IPed)	0.06±0.00	0.06±0.00	0.035	0.00	0.01	2.38	2.38	9.72
Substantia Nigra (SN)	0.29±0.01	0.26±0.01	0.034	0.00	0.05	2.40	2.40	9.56
Brainstem Rest (BS)	14.87±0.20	13.69±0.41	0.023	0.20	2.18	2.61	2.61	8.67
Superior Colliculus (SC)	2.14±0.04	1.97±0.03	0.009	0.05	0.28	3.10	3.10	8.35
Ventral Thalamic Nuclei (VT)	0.79±0.02	0.73±0.01	0.010	0.02	0.10	3.07	3.07	7.96
Fornix (fx)	0.08±0.00	0.07±0.00	0.016	0.00	0.01	2.79	2.79	7.56
Thalamus Rest (THAL)	3.54±0.05	3.31±0.04	0.006	0.08	0.38	3.34	3.34	6.95
Caudate Putamen (CPu)	5.08± 0.11	4.80±0.06	0.046	0.01	0.56	2.23	2.23	5.86
Optic Tract (ot)	0.15±0.00	0.16±0.00	0.044	-0.02	0.00	-2.25	-2.25	-7.75
Fimbria (fi)	0.45±0.01	0.50±0.01	0.006	-0.07	-0.02	-3.36	-3.36	-8.60
Stria Terminalis (st)	0.06±0.00	0.07± 0.00	0.015	-0.02	0.00	-2.85	-2.85	-14.76
Anterior Commissure (ac)	0.21±0.01	0.25±0.01	0.006	-0.07	-0.02	-3.32	-3.32	-17.62
Olfactory Areas (Olf)	5.50±0.13	6.74±0.26	0.001	-1.89	-0.60	-4.22	-4.22	-18.46
Aqueduct (Aq)	0.03±0.00	0.02± 0.00	0.062	0.00	0.02	2.06	2.06	49.13
Ventricular System (VS)	0.69±0.04	0.59±0.01	0.056	0.00	0.20	2.11	2.11	16.26
Inferior Colliculus (IC)	1.17±0.02	1.10±0.02	0.054	0.00	0.14	2.14	2.14	6.43
Hypothalamus (Hyp)	1.77±0.02	1.70±0.03	0.075	-0.01	0.15	1.95	1.95	4.25
Hippocampus (HIP)	5.52±0.10	5.75±0.06	0.074	-0.50	0.03	-1.96	-1.96	-4.09
Cerebral Peduncle (cp)	0.27±0.01	0.29±0.01	0.079	-0.04	0.00	-1.92	-1.92	-6.79
Amygdala (Amy)	2.42±0.08	2.61±0.06	0.060	-0.40	0.01	-2.07	-2.07	-7.46

Supplementary Table 6. ***Shank3***$^{\Delta e4\text{-}22}$ **homozygous mutant mice recapitulate major features of human *SHANK3* related disorders**

**Clinical features**	**Human[i] *SHANK3***	**ASDs**	**Mouse *Shank3***$^{\Delta e4\text{-}22\text{-}/\text{-}}$
Macrocephaly	20%	~30%	+increased brain size
Delayed speech	75%	variable	+aberrant USVs
ID/DD	>75	45%	+ instrumental learning/MWM
ASD	75%	100%	
Impaired communication			+ aberrant USVs
Impaired social interaction			+nest preference, social hierarchy
Inflexible behaviors			+hole board, reversal in MWM
Repetitive behaviors			+ self-grooming
Hair pulling	40%	ND[ii]	+ self-grooming
Self-injury behaviors	45%	<50%	+ skin lesions
Impulsivity	47%	~50%	+ escaping
Anxiety-like	ND*	42-56%	+ open field /light-dark box
Motor delay	75%	<75%	+ rotarod
Hypotonia	75%	25%	+ delayed neonatal paw position
Sensitivity to touch, unusual response to environmental stimuli	45%	50%	+ reactivity to handling
Seizure	25%	8-30%	Not observed
High pain threshold	77%	25%	Not tested
Aggressive behaviors	28%	<68%	Not observed

[i] Summarized from the following papers: [10, 11, 12, 13]

[ii] ND not determined

Supplementary Methods

Generation of *Shank3* Mice with Deletion of Exons 4-22 (Δe4-22)

The targeting constructs were prepared using a previously described recombineering method [14].The 129/SvEv BAC clone (bMQ457K21) covering the *Shank3* gene was first identified *in silico* using the Ensembl mouse genome browser (www.ensembl. org) and the clone was obtained from Geneservice Ltd, UK[15].*Shank3*$^{\Delta e4-22}$ mice were generated by a two-step targeting strategy using the Cre-*loxP* system. The first (5') construct inserted the *loxP1* and *loxP2* sites flanking exons 4-9 with a neomycin cassette. The second (3') construct inserted the *loxP3* at a 3' site 5 kb-downstream of exon 22 with a puromycin cassette **(Fig. 1a and Supplementary Fig. 1b)**. These constructs were sequentially electroporated into AB2.2 ES cells from the 129/SvEv strain as described[16]. DNA from ES cells was analyzed by mini-Southern blot hybridization using probes for the 5' and 3' targeting constructs. Successfully targeted cells were expanded and injected into C57BL/6J blastocysts for germ-line transmission as described[16]. *Shank3* mice with exons 4-22 floxed were bred with CMV-Cre mice (Jackson Labs, Stock No. 006054, Bar Harbor, ME) and the offspring with recombination between the *loxP1* and *loxP3* sites were identified by genotyping with PCR. Mice carrying the *Shank3* exons 4-22 (Δe4-22) deletion were backcrossed onto a C57BL/6J (Jackson Labs, Stock No. 000664) background for more than 5 generations for most of the molecular studies and for more than 8 generations for all behavioral experiments. A natural *Disc1* mutation in 129/SvEv mice was segregated from the *Shank3* targeted mutation during the backcrossing[3]. All experiments were conducted with protocols approved by the Institutional Animal Care and Use Committee at Duke University.

Preparation of Crude PSD and Cytosolic Fractions

Isolation of crude PSDs was performed using a previously described protocol[17] with some modifications. Protease and phosphatase inhibitors were used throughout, except in the final centrifugation step. Tissues from different regions of Δe4-22 brains were homogenized in HEPES-buffered sucrose (0.32 M sucrose, 4 mM HEPES, pH 7.4) and centrifuged at 800 × g for 10 min at 4°C. The cloudy supernatants were collected and transferred to a new set of tubes. After 12,000 × g centrifugation for 15 min at 4°C, the pellet (P2) and the supernatant (S2) were separated. The S2 fraction was centrifuged at 20,500 × g for 15 min at 4°C to obtain the purified

cytosolic fraction. The P2 fraction was lysed using water, then buffered with HEPES (pH 7.4) to 4 mM, and the sample was mixed by rotation at 4°C for 30 min, followed by centrifugation at 20,500 × g for 30 min to yield the P3 synaptosomal membrane (SPM) fraction. The SPM was resuspended in a buffer containing 50 mM HEPES (pH 7.4), 2 mM EDTA, and 0.5% Triton X-100.After 15 min of mixing by rotation at 4°C, the crude PSD-I fraction was obtained by centrifugation at 32,000 × g for 20 min at 4°C. The crude PSD pellet was dissolved in 1% SDS-PBS for further quantitative immunoblot analysis.

Quantitative Immunoblot Analysis

Equal amounts of proteins from whole cell lysates, cytosolic fractions, or crude PSD fractions were separated by SDS-PAGE. Proteins were transferred to PVDF membranes (Bio-Rad, Hercules, CA). After blocking the membrane at room temperature for 1 hr in TRIS-buffered saline (pH 7.4, TBS) with 5% non-fat milk, the blots were incubated with corresponding primary antibodies at 4°C overnight. The blots were washed in TBS containing 0.1% Tween-20 (TBST) and incubated with HRP-conjugated secondary antibodies for 60 min at room temperature. Following 3 washes in TBST, the blots were incubated with ECL reagent (GE Healthcare Life Sciences, Piscataway, NJ) and exposed to Kodak X-ray film (Rochester, NY).For quantification, the films were scanned by a UVP image system (Upland, CA), the gray values of proteins were analyzed by ImageJ software (NIH, Bethesda, MD; http://rsb.info.nih.gov/ij/), and normalized to that of corresponding internal controls, actin or β-tubulin III.

Antibodies

The PSD-95 (K28/43), Pan-SAPAP (N127/31), GluA2 (L21/32), and GluN2B (N59/36) antibodies were purchased from UC Davis/NIH NeuroMab. The ERK1/2 (9102), phospho-ERK1/2 (Thr202/Tyr204)(9101), S6K (9202), phospho-S6K (Thr389)(9206), mTOR (2972), and phospho-mTOR (Ser2448)(2971) antibodies were bought from Cell Signaling Technology (Danvers, MA). The actin (sc-1615), Homer1b/c (sc-20807), Homer1a (sc-8922), Homer2 (sc-8924), Homer3 (sc-271653), Shank2 (sc-23545), Shank3 (C-terminal) (sc-30193), and PSD-93 (Sc-12233) antibodies were obtained from Santa Cruz Biotechnology (Santa Cruz, CA). The GluN2A (07-632) and mGluR5(AB5675) antibodies were from Millipore (Billerica, MA). The mGluR5 (extracellular)(AGC-007) antibody was from Alomone Labs (Jerusalem, Israel). The

GluA1 (ab31232), SAPAP-3 (ab67224), and β-tubulin III (ab18207) antibodies were purchased from Abcam (Cambridge, MA). The Bassoon (SAP7F407) antibody was from Stressgen (Ann Arbor, MI). The Shank1 (HPA032129) antibody was from Sigma-Aldrich (St. Louis, MO). The puromycin (3RH11) antibody was from KeraFAST (Boston, MA). The rat DARPP-32(MAB4230) antibody was from R&D systems (Minneapolis, MN). The Ncald (66088) antibody was from Proteintech Group (Chicago, IL). The Shank3 N-terminal antibody was a generous gift from Dr. Paul Worley at Johns Hopkins University.

Co-immunoprecipitation

Co-immunoprecipitation of mGluR5 and Homer1b/c was performed using proteins from the synaptosomal fraction. Striata from $\Delta e4\text{-}22^{+/+}$ or $\Delta e4\text{-}22^{-/-}$ mice were homogenized in 4 mM HEPES buffer (pH 7.4) containing 0.32 M sucrose. The tissue homogenate was centrifuged at 960 × g for 10 min at 4°C; the supernatant was centrifuged at 20,500 × g at 4°C for 15 min. This high-speed pellet (P2) was washed and resuspended in 1× PBS (pH 7.4) containing 1% Triton X-100 (binding buffer) and mixed by rotation at 4°C for 20 min. After clearing by centrifuging at 20,500 × g at 4°C for 15 min, the protein concentration of the supernatant was measured and 100 µg of lysate was incubated with 2 µg of mGluR5 antibody overnight at 4°C. Immune complexes were then precipitated by incubation with 50 µl protein A/G agarose beads for 1 hr followed by centrifugation (100 × g for 1 min at 4°C). Precipitated proteins were washed 3 times with binding buffer and once with binding buffer without Triton X-100, prior to elution by boiling in 2× SDS-PAGE sample buffer. Samples were further analyzed by western blot using corresponding antibodies.

Immunocytochemistry and Confocal Microscopy

Eight-week-old mice were transcardially perfused with 4% paraformaldehyde in 1× PBS (pH 7.4). The brains were dissected and post-fixed in the same fixative overnight. The brains were then cryoprotected by submerging them in 30% sucrose for 48 hr. After embedding into O.C.T. medium on dry ice, brains were sectioned on a cryostat at 40 µm thickness. Slices with brain regions of interest were permeabilized and blocked in a solution containing 1× PBS (pH 7.4), 2% BSA, and 0.2% Triton X-100 for 60 min. Primary antibodies with appropriate dilutions were added and incubated overnight at 4ºC, followed by 3× 10 min washes in 1×PBS (pH 7.4). Alexa

488 or Alexa 568 conjugated secondary antibodies were incubated for 1 hr at room temperature. Three further washes (15 min each) were performed before cover-slips were mounted onto glass slides using gel mount (EMS, Hatfield PA). DAPI was used to stain DNA to mark nuclei. Confocal images were obtained using a 100× objective (numerical aperture, 1.4) with sequential acquisition settings of 1024×1024 pixels. Each image was a z-series projection of 3-4 images, each single image was taken at 0.5 µm depth intervals and averaged 4 times. Morphometric analysis and quantification were performed using ImageJ software (NIH, Bethesda, MD) by experimenters who were blinded to the genotypes. Co-localization (Pearson's correlation coefficient) of proteins was analyzed using ImageJ with the JACoP plugin.

Corticostriatal Co-cultured Neurons and mGluR5 Surface Immunostaining

Corticostriatal co-cultures were prepared from P1 littermate $\Delta e4\text{-}22^{+/+}$ and $\Delta e4\text{-}22^{-/-}$ mice according to a method described previously[18]. Briefly, following decapitation, striatal and cortical tissues were dissected and digested with trypsin. After inactivation of trypsin, tissues were briefly centrifuged and then dissociated in Neurobasal/B27 medium. Cortical and striatal cells were plated at a 1:1 ratio at a density of 100,000 cells/well in 12-well plates coated with 1 mg/ml poly-D-lysine. On DIV 13–14, cultures were fixed with 4% paraformaldehyde for 10 min at room temperature. Cultures were then rinsed with 1×PBS (pH 7.4) and blocked in 1×PBS (pH 7.4) with 2% BSA buffer for 60 min at room temperature. Cultures were stained with anti-mGluR5 (N-terminus) rabbit polyclonal antibodies (1:200) overnight at 4°C, followed by incubation with Alexa 568 conjugated goat anti-rabbit IgG (1:200) for 12 hr to saturate the mGluR5 antibodies. Cultures were then rinsed with 1×PBS (pH 7.4), permeabilized, and blocked in a buffer containing 1×PBS (pH 7.4), 0.2% Triton X-100, and 2% BSA for 60 min. Next, cultures were incubated with rat anti-DARPP-32 (1:100), a marker used to identify MSNs, and rabbit anti-mGluR5 (N-terminus) antibody (1:200) for 2 hr at room temperature. Cultures were then rinsed and incubated with the appropriate secondary antibodies (Alexa 488-conjugated goat anti-rat IgG, 1:500; Alexa 405-conjugated goat anti-rabbit IgG, 1:500; **Life Technologies,** Grand Island, NY) for 2 hr at room temperature. Three further washes (15 min each) were performed before coverslips were mounted onto glass slides using gel mount. Images were collected with a 63× objective on an LSM 510 confocal microscope (Zeiss). All mGluR5 images were collected under identical acquisition conditions and sub-saturating conditions. Dendrites were selected if

they were clearly part of a cell with high intensity DARPP-32 staining and did not have other overlying dendrites. For quantitative analysis, the average mGluR5 immunostaining intensity and the ratio between surface (red) and intracellular (blue) mGluR5 were measured on DARPP-32-positive (green) dendritic and soma regions with Image J software.

Golgi Staining

Golgi staining was performed using 8-week-old mice as described [3]. The FD Rapid Golgi Stain kit (FD NeuroTechnologies, Boston, MA) was employed; freshly dissected brains were immersed in solutions A and B for 2 weeks at room temperature, then transferred into solution C for at least 48 hr at 4°C. The brains were sliced using a cryostat (Leica CM3050 S) at a thickness of 100 µm. A 63× objective (Leica DMI 6000B) was used to obtain a bright-field image series of sections (1024×1024 pixels, 0.09 µm/pixel) at 0.5 µm intervals. Neurons from two regions of interest were captured: pyramidal neurons in the hippocampal CA1 region and MSNs in the striatum. All imaging and analysis were performed in a blinded manner. For quantification, 30-50 µm dendritic segments were identified and protrusions were measured manually.

Electron Microscopy

Electron Microscopy was performed using 8-week-old mice as described [3]. Anesthetized animals were perfused with a mixture of 2% glutaraldehyde and 2% depolymerized paraformaldehyde in 0.1 M PBS. Brains were removed and postfixed overnight in the same fixative at 4°C, then transferred to PBS. Fifty µm-thick sections were cut on a Vibratome. Sections containing regions of interest were treated with osmium tetroxide and uranyl acetate, then dehydrated, infiltrated with Spurr's resin, flat-embedded between sheets of ACLAR plastic sandwiched between glass coverslips, and polymerized for 48 hr at 60°C. Chips of tissue from regions of interest in CA1 hippocampus and striatum were glued to plastic blocks. Thin (~80 nm-thick) sections were cut on an ultramicrotome, collected on copper mesh grids, poststained with uranyl acetate and Sato's lead, and examined with a Philips Tecnai 12 TEM operated at 80 KV. For each grid we photographed 20 synapses with a Gatan 1024 x 1024 12-bit cooled CCD. Synapses were selected that exhibited clear membrane appositions, suggesting that they were cut approximately normal to the plane of the synaptic membrane. All data collection and analysis were performed in a blinded manner. Measurement was performed off-line, using ImageJ

software. For each synapse, we measured the length of the synaptic apposition, and the estimated thickness of the "dark" (membrane-facing) and "light" (cytoplasm-facing) parts of the postsynaptic density. Data were compiled and analyzed using Kaleidagraph software.

Olfactory Testing

The ability of mice to discriminate urine from pooled adult estrus females relative to 0.9% NaCl was examined. Thirty µL of urine or saline were applied to filter paper (Whatman 3MM; Sigma-Aldrich, St Louis, MO), placed into Tissue-Tek® cassettes, and positioned at opposite ends to a rat cage. Mice were habituated to an empty cage for 5 min prior to introduction of the first stimulus. Each stimulus presentation lasted 5 min and the stimuli were presented in a randomized order. The time spent sniffing each stimulus was recorded by a blind observer with a stopwatch. Preference ratios for the urinary olfactant were calculated and expressed as the time spent with the urinary minus time spent with the saline cue, divided by the total time spent with both cues. Olfactory habituation-dishabituation was tested as described[19] with 2 min presentations of olfactory stimuli on cotton-tipped swabs in triplicate in this order: water, maple extract (diluted 1:100 in water), banana extract (diluted 1:100 in water), soiled bedding from C57BL/6J female mice, and finally soiled bedding from C3H/HeJ female mice.

Neonatal Developmental Milestones

The developmental milestones of neonates were assessed on postnatal day 4. Individual pups were removed from the litter and placed on a heating-pad maintained at 23-25°C. All assessments were completed within 75-90 s. First, pups were observed for overall appearance and whether the ears were detached from (open) or attached to (closed) the head. For the rooting reflex test, the pup's face was bilaterally stimulated with the experimenter's forefingers. If the pup crawled forward and pushed its head in a rooting fashion during the stimulation, the rooting reflex was considered evident. In the cross-extensor reflex test, one of the pup's hind-limb feet was pinched. If the pup's opposite hind-limb extended in response to the pinch, the cross-extensor reflex was scored evident. For the negative geotaxis test, the pup was placed on a 45° high-friction incline with its head facing downwards. If the pup moved its body so that its head was facing upwards within 20 s, negative geotaxis was considered present (most control pups will turn on the inclined plane within 10 s). For the paw position test, the pup had to stand with

all four paws flat on the floor that were in line with the appropriate shoulder/pelvic region. In the self- righting test, pups were placed on their back. If the pup turned over on its belly within 15 s, self-righting was scored as present. The last test determined whether milk was present in the pup's stomach. After assessments, the pup was returned to their dam in the home cage.

Social Hierarchy

Thirty-three mice were housed for 6 days in triads with non-familiar animals of the same sex, age, and genotype. All animals were given unique dye markings on their backs 48-72 hr before testing. Cages were filmed from the top with high-speed low-light video cameras interfaced to Noldus Media Recorder once every hr for 10 min. Videos were scored by coders blinded to the sex or genotype of the animals. Mice were scored for behavioral interactions that consisted of non-aggressive social approaches, including sniffing or nosing the tail and anogenital areas, and agonistic behaviors that included climb-grooming along the back of the partner mouse, chasing, mounting, bites or fighting behaviors. Dominance was scored by tabulating the total agonistic behaviors initiated by each mouse within the cage as described[20]. Animals with the most agonistic responses were classified as dominant, mice with fewer agonistic initiations were scored as subordinate, and the mouse with the least or no agonistic initiations was considered submissive. Hierarchical organization within the cage was classified as present and stable or unstable and delayed. A present and stable hierarchy consisted of those cages on day 1 that had a hierarchy with a dominant animal, whose hierarchy was maintained throughout testing to day 6. Cages with unstable hierarchies consisted of those where dominance changed across the 6 days. A delayed hierarchy was one where no dominant animal was observed until day 6 of testing. As no sex differences were found in the overall expression of agonistic behaviors and dominance hierarchies were as likely to be formed in female as in male cages, the data were collapsed across sex within each genotype.

Sociability Test

Mice were examined for sociability as described with a few modifications[21]. Testing was conducted in a white acrylic apparatus (i.e., no walls dividing the chamber) with two stainless-steel wire-mesh cages (10 cm diameter x 11 cm high; Rolodex, Oak Brook, IL) each stabilized with a 28 g lead sinker (Cabella, Oshkosh, NE) to prevent the cage from being moved or tipped

during testing; a clear plexiglass sheet was placed over the chamber to prevent escape. One week prior to testing, 10-week-old C3H/HeJ female mice (Jackson Labs) were handled for 5 min and were trained to sit inside the wire-mesh cages in the test arena for 20 min a day for 5 consecutive days. These animals were used subsequently as the social stimuli during testing. Testing was divided into three phases. During each test phase, the cages were placed in the center of the two outer thirds of the chamber. Test phase 1 began when a Δe4-22 mouse was placed into the center of the chamber and given free exploration of the apparatus with empty wire-mesh cages. At the end of 10 min, the Δe4-22 target mouse was removed and one of the cages was replaced with an identical cage containing the novel partner C3H mouse. Test phase 2 (social affiliation) began with reintroduction of the target mouse into the center of the chamber. At the end of 10 min, the Δe4-22 target was removed; the empty wire-mesh cage was removed and replaced with a new cage containing a novel C3H partner. Test phase 3 (social preference) was terminated after 10 min. All tests were filmed and the digital videos were analyzed subsequently using EthoVision software (Noldus) that included the frequency and duration of contacts with each cage. Preference scores were calculated, where time spent with one stimulus (non-social stimulus 1, social stimulus 1, or novel social stimulus 2) was subtracted from the time spent with the other stimulus (non-social stimulus 2, non-social stimulus, and familiar social stimulus 1, respectively) and divided by the total time spent exploring both stimuli. Positive scores indicated a preference for the novel social stimulus relative to the non-social or familiar social stimulus, whereas negative scores reflected preference for the non-social or familiar stimulus; and scores approximating “0” indicated no preference.

Grip-Strength Test

Strength of the front and rear paws was assessed with a mouse grip-strength meter (San Diego Instruments, San Diego, CA) and was reported as units of g-force. Each mouse was given 3 trials using the front paws only and 3 trials using all 4 paws.

Prepulse Inhibition

Prepulse inhibition (PPI) was examined as described using the SRL-Lab startle response system and software (San Diego Instruments, San Diego, CA)[26]. Testing consisted of three types: null trials with only a white-noise background (64 dB) and no auditory stimulus; pulse-alone

trials with the 40 ms 120 dB white-noise stimulus; and prepulse-pulse trials, where the startle stimulus was preceded by 100 ms with a 20 ms prepulse stimulus that was 4, 8, or 12 dB above the white-noise background. Mice were placed into plexiglass holders and acclimated to the apparatus for 5 min before testing. Each test consisted of 74 trials with 30 pulse-alone trials, 8 null trials, and 36 prepulse-pulse trials. Trial order was the same for all animals, with 10 pulse-alone trials followed by combinations of the prepulse-pulse and null trials, and terminating with 10 pulse-alone trials. Responses were measured as the maximum startle response (mAmp platform displacement) following presentation of the pulse or startle stimulus or during the null trials. PPI was calculated as the ratio of the startle responses on prepulse trials to startle-only trials, subtracted from 1 and expressed as a percentage [1 - (prepulse-pulse trials/pulse-alone trials)*100].

Morris Water Maze and Visible Platform Testing

Spatial learning and memory, and plasticity were examined in the Morris water maze as described[3]. All training and testing were conducted under ~125 lux illumination in a 120 cm diameter stainless-steel pool filled with water, made opaque with white non-toxic poster paint (Crayola LLC, Easton, PA) and maintained at 24°C. The pool was divided into four quadrants; northeast (NE), northwest (NW), southeast (SE) and southwest (SW). Before testing, mice were handled for 10 min and then acclimated to standing in water for 1 min over 5 consecutive days. Next, mice were trained to sit on the hidden platform (1 cm below the water's surface and 20 cm from the rim of the pool) in the NE quadrant for 20 s and then allowed to swim freely for 60 s before being returned to the platform for 15s. On the following day, water-maze testing began with testing divided into 2 phases: acquisition (days 1-6) with the hidden platform in the NE quadrant and reversal (days 7-12) with the platform in the SW quadrant. Each day the mice received 4 trials in pairs that were separated by 60 min. Release points were randomized across test-trials and test-days. On days 2, 4, 6, 8, 10, and 12, a single probe trial was given 1 hr after the 4 test-trials. For probe trials, the platform was removed from the water and the mice were released from the southern-most point on days 2, 4, and 6, and from the northern-most point on days 8, 10, and 12. In addition to acquisition and reversal training, the same cohort of mice was used for visible platform testing conducted over 3 consecutive days again with 4 trials a day. Here, the mice were released from the northern-most point and given 60 s to swim to the visible

platform (a 5 x 5 cm patterned flag was suspended 24 cm above the platform). The platform location was changed on each trial to a new, randomized location. Performance on all tests was scored by Ethovision XT 7 (Noldus) using a high-resolution camera suspended 180 cm above the center of the pool. Tracking profiles were generated by Ethovision software and were used to measure swim time. Except for probe trials (60 s in duration), all trials ended when the animal reached the platform or after 60 s of swimming.

Fear Conditioning

Animals were tested for contextual and cued fear conditioning as described[22]. Mice were conditioned and tested in Med-Associates fear conditioning chambers under ~100 lux illumination. On day 1, mice were placed in the chamber for 2 min, after which a 72-dB 12-kHz tone (conditioned stimulus, CS) was presented for 30 s, which terminated simultaneously with a 2 s 0.4-mA scrambled foot-shock (unconditioned stimulus, UCS). Mice were removed from the conditioning chamber to the home cage 30 s later. For context testing on day 2, animals were returned to the chamber in which they had been conditioned for 5 min in the absence of the CS and UCS. For cued testing on day 3, the dimensions, texture and shape of the conditioning chamber were modified. Mice were introduced into the chamber for 2 min, after which the CS was presented for 3 min. For all tests, behavior was videotaped and scored in an automated fashion by FreezeScan (Cleversys, Reston VA) for freezing.

Image Segmentation and Analysis for Magnetic Resonance Histology (MRH)

Brain images for each animal were segmented into 40 major regions using a segmentation pipeline implemented in Perl[23, 24, 25], advanced normalization tools[26], and a manually segmented DTI atlas of the mouse brain based on images acquired in a separate study[25].

An existing cohort of C57BL/6J specimens was used as controls to assess volumetric differences. A two-tailed t-test was used to identify differences between genotypes for volume regional values, for each of the 40 regions, normalized to the brain volume. A $p<0.05$ was considered significant. Only 6 of these 7 C57BL/6J animals and 6 △e4-22$^{-/-}$ mice were used for the DTI parametric analysis due to insufficient SNR values in 2 specimens. DTI parametric analysis was not performed directly because of a systematic bias in SNR values between the

genotypes, instead correlational analysis using Pearson coefficients was performed within each genotype, and these results were then compared semi-quantitatively between genotypes.

Voxel-based statistics were performed after generating a minimum deformation template, to which all images were registered. Registration was performed using ANTs[26]. Deformation based morphometry was performed using SurfStat[27] on the smoothed (3 voxels) log Jacobians of the deformation fields generated during registration, and voxel wise statistics on the fractional anisotropy images mapped into the space of the template.

Preparation of Striatal Slices for Whole-cell Patch-Clamp Recording

After acute isoflurane anesthesia, adult mice (2-5 mos) from the 3 genotypes were decapitated and their brains were removed and placed in ice-cold solution bubbled with 95% O_2 - 5% CO_2 containing the following in mM: 194 sucrose, 30 NaCl, 2.5 KCl, 1 $MgCl_2$, 26 $NaHCO_3$, 1.2 NaH_2PO_4, 10 *D*-glucose. After 5 min, 250 µm coronal slices were cut using a Vibratome. During the recovery period slices were placed in 35°C oxygenated ACSF solution containing the following in mM: 124 NaCl, 2.5 KCl, 2 $CaCl_2$, 1 $MgCl_2$, 26 $NaHCO_3$, 1.2 NaH_2PO_4, 10 *D*-glucose. All recordings were made under continuous perfusion of ACSF at 29.5 °C with a 2-3ml/min flow rate. Pipettes (2.5–5 MΩ) for voltage-clamp experiments contained the following in mM: 120 cesium methane sulfonate, 5 NaCl, 10 tetraethylammonium chloride, 10 HEPES, 4 lidocaine *N*-ethyl bromide, 1.1 EGTA, 4 magnesium ATP, and 0.3 sodium GTP, pH adjusted to 7.2 with CsOH and osmolarity set to 298 mOsm with sucrose. The internal solution for current-clamp experiments contained in mM: 150 potassium gluconate, 2 $MgCl_2$, 1.1 EGTA, 10 HEPES, 3 sodium ATP, and 0.2 sodium GTP, with pH adjusted to 7.2 with KOH and osmolarity set to ~ 300 mOsm with sucrose.

LFP Oscillatory Power and Cross-area Coherence

Signals recorded from all implanted microwires were used for analysis. Using Matlab (The MathWorks, Inc., Natick, MA), a sliding window Fourier transform with Hamming window was applied to the LFP signal using a 1 second window with a 1 second step. Frequencies were analyzed with a resolution of 0.5 Hz. Power was averaged across each recording period of interest. LFP coherence was calculated from LFP pairs using magnitude-squared coherence

$$C_{AB}(f) = \frac{|Psd_{AB}(f)|^2}{Psd_{AA}(f)Psd_{BB}(f)}$$

where coherence is a function of the power spectral densities of A and B, and their cross-spectral densities (The MathWorks, Inc., Natick, MA). These calculated coherence values were then averaged across all wires recorded from a given brain area pair. This final coherence value was used for analysis.

LFP Phase Analysis

Our analytical methods for calculating single unit phase locking, LFP directionality analysis, and cross-frequency phase coupling have been previously described[28, 29]. Briefly, LFP data was filtered using Butterworth bandpass filters designed to isolate LFP oscillations within a 2 Hz window using a 1 Hz step. The instantaneous phase of the filtered LFPs was then determined using the Hilbert transform, and the instantaneous phase offset between two areas $(\phi_{Area1} - \phi_{Area2})_t$ was calculated for each time point. The deviation from circular uniformity for the phase offset time series was then calculated using the mean resultant length (MRL). Next, we introduced temporal offsets [-250 ms 250 ms] between the two LFP's and recalculated the MRL at each temporal offset. The temporal offset for optimal phase coupling was determined for each frequency band as the offset at which the highest mean resultant length was observed. Cross frequency phase coupling using the modulation index has been previously described[30, 31, 32]. The Modulation index was calculated as the average modulation value observed across all of the microwires implanted in each brain area. Phase Locking was calculated based on the MRL of sum of LFP phases at which unit firing occurred[28]

NAC Selective *Shank3* Knockdown

At 10-12 weeks of age, 11 female mice with the *Shank3* e4-22 segment floxed were anesthetized with isoflurane (1.5%), and placed in a stereotaxic device. Injections of 0.5uL of either AAV10-Cre or AAV10-GFP were given bilaterally into the NAC core. The injection was done at a 10º angle, with the coordinates of AP: +1.25, ML: +1.79, DV: -4.57. Virus was administered at a rate of 0.1uL per minute, and needles were kept in place for an additional 10

minutes before slow withdraw. Animals were sutured and treated with post-operative analgesics (carprofen, 5mg/kg). Four weeks after the initial viral surgery, mice were anesthetized again with isoflurane (1.5%), and placed in a stereotaxic device. Electrodes were implanted in NAC as described above. Two weeks after surgical recovery, unit activity was recording from NAC while mice were subjected to the first half of the forced interaction test (e.g. baseline).

Statistical Analyses of *in vivo* Data

Long range functional connectivity analysis was performed using two approaches. In the first approach we identified a test-activated network in C57B/6J mice and used this network to constrain our statistical analysis of the test-genotype effects in the mutants and their littermate controls. In the second approach, we used the mutants and their littermate controls to discover the test related network, and used this network to constrain our analysis of the test-genotype effects.

First, a cohort of twelve 12 week-old C57BL/6J mice was implanted with recording electrodes and their circuit responses were measured during the forced social interaction test. Significant test-condition (empty outside area, vs. C3H mouse condition) effects were determined using a Wilcoxon sign-rank for the 40 coherence measures (see Supplementary Table 5). We then limited our genotype x test-condition analysis in the *Shank3* mutants and their Δe4-22$^{+/+}$ littermates to the “test-related” network identified in the C57BL/6J mice (e.g. coherence measures that showed significant test effects) with a FDR-correction for multiple comparisons. Notably, since the social network was initially defined in a group of normal animals, this approach had the potential to overlook test-related network changes that occurred exclusively in the mutant animals. Second, connectivity data from the Shank3 mutants and their Δe4-22$^{+/+}$ littermates was analyzed using a mixed model ANOVA of the genotype by test-condition with a Box-Cox transformation, followed by Bonferroni-corrections for multiple comparisons. The “test-related” network for this approach was defined as the compilation of the coherence measurements that demonstrated significant test-condition effects following the Bonferroni-correction. We then tested for genotype by test-condition effects selectively across this network using a FDR-correction. The disadvantage of this second approach is that it does not fully correct for multiple comparisons, biasing the analysis towards false discoveries. Nevertheless, both of our statistical approaches yielded the same genotype x test-condition related changes (i.e.,

cortical-striatal-thalamic 7-11 Hz circuit changes). The latter approach is presented in the main text, and the results of all statistical tests used for both approaches are presented in the supplement. Coherence data were depicted using Circos[33]. In all cases, a $p<0.05$ was considered significant. For comparisons of test-related power within genotype, *post-hoc* testing was performed using a Wilcoxon sign-rank test.

Supplementary References

1. Bozdagi O, *et al.* Haploinsufficiency of the autism-associated Shank3 gene leads to deficits in synaptic function, social interaction, and social communication. *Mol Autism* **1**, 15 (2010).

2. Peca J, *et al.* Shank3 mutant mice display autistic-like behaviours and striatal dysfunction. *Nature* **472**, 437-442 (2011).

3. Wang X, *et al.* Synaptic dysfunction and abnormal behaviors in mice lacking major isoforms of Shank3. *Hum Mol Genet* **20**, 3093-3108 (2011).

4. Schmeisser MJ, *et al.* Autistic-like behaviours and hyperactivity in mice lacking ProSAP1/Shank2. *Nature* **486**, 256-260 (2012).

5. Kouser M, *et al.* Loss of predominant Shank3 isoforms results in hippocampus-dependent impairments in behavior and synaptic transmission. *J Neurosci* **33**, 18448-18468 (2013).

6. Lee J, *et al.* Shank3-mutant mice lacking exon 9 show altered excitation/inhibition balance, enhanced rearing, and spatial memory deficit. *Frontiers in cellular neuroscience* **9**, 94 (2015).

7. Duffney LJ, *et al.* Autism-like Deficits in Shank3-Deficient Mice Are Rescued by Targeting Actin Regulators. *Cell reports* **11**, 1400-1413 (2015).

8. Zhou Y, *et al.* Mice with Shank3 Mutations Associated with ASD and Schizophrenia Display Both Shared and Distinct Defects. *Neuron* **89**, 147-162 (2016).

9. Speed HE, *et al.* Autism-Associated Insertion Mutation (InsG) of Shank3 Exon 21 Causes Impaired Synaptic Transmission and Behavioral Deficits. *J Neurosci* **35**, 9648-9665 (2015).

10. Sarasua SM, *et al.* Association between deletion size and important phenotypes expands the genomic region of interest in Phelan-McDermid syndrome (22q13 deletion syndrome). *J Med Genet* **48**, 761-766 (2011).

11. Soorya L*, et al.* Prospective investigation of autism and genotype-phenotype correlations in 22q13 deletion syndrome and SHANK3 deficiency. *Molecular autism* **4**, 18 (2013).

12. Phelan K. 22q13.3 Deletion Syndrome. In: *Genetest* (ed^(eds Roberta A Pagon E-i-c, Thomas C Bird, Cynthia R Dolan, and Karen Stephens.). Internet (2007).

13. Bonaglia MC*, et al.* Molecular Mechanisms Generating and Stabilizing Terminal 22q13 Deletions in 44 Subjects with Phelan/McDermid Syndrome. *PLoS genetics* **7**, e1002173 (2011).

14. Liu P, Jenkins NA, Copeland NG. A highly efficient recombineering-based method for generating conditional knockout mutations. *Genome Res* **13**, 476-484 (2003).

15. Adams DJ*, et al.* A genome-wide, end-sequenced 129Sv BAC library resource for targeting vector construction. *Genomics* **86**, 753-758 (2005).

16. Jiang YH*, et al.* Mutation of the Angelman ubiquitin ligase in mice causes increased cytoplasmic p53 and deficits of contextual learning and long-term potentiation. *Neuron* **21**, 799-811 (1998).

17. Carlin RK, Grab DJ, Cohen RS, Siekevitz P. Isolation and characterization of postsynaptic densities from various brain regions: enrichment of different types of postsynaptic densities. *J Cell Biol* **86**, 831-845 (1980).

18. Chen M, Wan Y, Ade K, Ting J, Feng G, Calakos N. Sapap3 deletion anomalously activates short-term endocannabinoid-mediated synaptic plasticity. *The Journal of neuroscience : the official journal of the Society for Neuroscience* **31**, 9563-9573 (2011).

19. Yang M, Crawley JN. Simple behavioral assessment of mouse olfaction. *Current protocols in neuroscience / editorial board, Jacqueline N Crawley [et al]* **Chapter 8**, Unit 8 24 (2009).

20. Rodriguiz RM, Chu R, Caron MG, Wetsel WC. Aberrant responses in social interaction of dopamine transporter knockout mice. *Behavioural brain research* **148**, 185-198 (2004).

21. Moy SS*, et al.* Sociability and preference for social novelty in five inbred strains: an approach to assess autistic-like behavior in mice. *Genes Brain Behav* **3**, 287-302 (2004).

22. Wetsel WC*, et al.* Disruption of the expression of the proprotein convertase PC7 reduces BDNF production and affects learning and memory in mice. *Proc Natl Acad Sci U S A* **110**, 17362-17367 (2013).

23. Badea CT, Bucholz E, Hedlund LW, Rockman HA, Johnson GA. Imaging methods for morphological and functional phenotyping of the rodent heart. *Toxicol Pathol* **34**, 111-117 (2006).

24. Jiang Y, Johnson GA. Microscopic diffusion tensor imaging of the mouse brain. *Neuroimage* **50**, 465-471 (2010).

25. Jiang Y, Johnson GA. Microscopic diffusion tensor atlas of the mouse brain. *Neuroimage* **56**, 1235-1243 (2011).

26. Avants BB*, et al.* The optimal template effect in hippocampus studies of diseased populations. *Neuroimage* **49**, 2457-2466 (2010).

27. Chung MK, Worsley KJ, Nacewicz BM, Dalton KM, Davidson RJ. General multivariate linear modeling of surface shapes using SurfStat. *Neuroimage* **53**, 491-505 (2010).

28. Kumar S, Hultman R, Hughes D, Michel N, Katz BM, Dzirasa K. Prefrontal cortex reactivity underlies trait vulnerability to chronic social defeat stress. *Nature communications* **5**, 4537 (2014).

29. Dzirasa K, Kumar S, Sachs BD, Caron MG, Nicolelis MA. Cortical-amygdalar circuit dysfunction in a genetic mouse model of serotonin deficiency. *J Neurosci* **33**, 4505-4513 (2013).

30. Canolty RT*, et al.* High gamma power is phase-locked to theta oscillations in human neocortex. *Science* **313**, 1626-1628 (2006).

31. Dzirasa K*, et al.* Lithium ameliorates nucleus accumbens phase signaling dysfunction in a genetic mouse model of mania. *J Neurosci* **30**, 16314 –16323 (2010).

32. Dzirasa K*, et al.* Hyperdopaminergia and NMDA receptor hypofunction disrupt neural phase signaling. *J Neurosci* **29**, 8215-8224 (2009).

33. Krzywinski M*, et al.* Circos: an information aesthetic for comparative genomics. *Genome Res* **19**, 1639-1645 (2009).
